# MFAP2 enhances cisplatin resistance in gastric cancer cells by regulating autophagy

**DOI:** 10.7717/peerj.15441

**Published:** 2023-06-07

**Authors:** Meng Li, Hong-Yi Zhang, Rong-Gui Zhang

**Affiliations:** 1 Department of Gastroenterology, General Hospital of the Chinese People’s Liberation Army, Beijing, China; 2 Department of Stomatology, Beijing Electric Power Hospital, Capital Medical University, Beijing, China

**Keywords:** MFAP2, Gastric cancer, Autophagy, CDDP-resistance

## Abstract

**Background:**

Cisplatin (CDDP) is of importance in cancer treatment and widely used in advanced gastric cancer (GC). However, its clinical usage is limited due to its resistance, and the regulatory mechanism of CDDP resistance in GC has not yet been fully elucidated. In this study, we first conducted a comprehensive study to investigate the role of MFAP2 through bioinformatics analysis.

**Methods:**

The Gene Expression Omnibus (GEO) and The Cancer Genome Atlas (TCGA) databases were applied to downloadgene expression data and clinicopathologic data, and the differentially expressed genes (DEGs) were further analyzed. Then, Gene Ontology (GO), Kyoto Encyclopedia of Genes and Genomes (KEGG) enrichment analysis and survival analysis were conducted. Furthermore, according to the clinicopathological characteristics of TCGA, clinical correlation analysis was conducted, and a receiver operating characteristic curve (ROC) was plotted.

**Results:**

We revealed that *FAP*, *INHBA* and *MFAP2* were good diagnostic factors of GC. However, the mechanism of MFAP2 in GC remains elusive, especially in the aspect of chemotherapy resistance. We developed the CDDP-resistant cell line, and found that MFAP2 was upregulated in CDDP-resistant cells, and MFAP2-knockdown improved CDDP sensitivity. Finally, we found that MFAP2 enhanced CDDP resistance by inducing autophagy in drug-resistant cell lines.

**Conclusions:**

The above results suggested that MFAP2 could affect the chemotherapy resistance by altering the level of autophagy in GC patients as a potential therapeutic target.

## Introduction

Gastric cancer (GC) has been one of the most common and deadly malignancies in the world since the 20th century (Luo et al., 2017; Sung et al., 2021). In spite of the continuous improvement in the diagnosis and medical treatments of GC, surgical treatment remains its standard treatment strategy (Sitarz et al., 2018). However, surgical treatment is still clinically limited in GC patients, because most of them are diagnosed at an advanced stage (Marghalani et al., 2020). In fact, chemotherapy is still an essential mean for the treatment of GC (Wagner et al., 2017). Nevertheless, chemotherapy for GC is prone to drug resistance, leading to chemotherapy failure or unsatisfactory efficacy (Marin et al., 2016). Therefore, exploring the mechanism of primary or acquired resistance to anti-GC drugs will be helpful to meliorate the efficacy and survival rate of GC.

Recently, studies have reported that autophagy plays a crucial part in tumor genesis and development, and a key regulatory role in tumor drug resistance (Xiu, Guo & Jing, 2021; Yao et al., 2020). Autophagy is considered as a highly conservative pathway in eukaryotic cell based on regulating autophagy-related genes (Cao et al., 2019). A double-membrane structure containing some proteins and organelles is usually formed during autophagy, which is called autophagosome. The autophagosomes derive from the rough surface endoplasmic, and fuse with lysosomes to construct the autophagy-lysosome, which can degrade the contents (Levy, Towers & Thorburn, 2017). Autophagy plays a significant role in the whole life process of cells in order to satisfy the needs of cellular metabolism and renewal of some organelles (Zhan, Li & Wei, 2018). As a essential survival mechanism, autophagy helps normal cells adjust and adapt to adverse environment, while inhibit tumorigenesis in tumor cells. However, in most cases, it contributes to tumorigenesis by helping tumors evade immune surveillance, hence causing tumor growth (White, 2015). In terms of cancer therapy, studies indicated that chemotherapy drugs can stimulate cancer cell apoptosis, while autophagy can avoid cancer cell apoptosis, thus causing the body to develop drug resistance (Shi et al., 2016; Xie, Liu & Li, 2020). In other words, cancer cells can also enhance their own drug resistance by inducing autophagy. For example, when 5-FU and cisplatin (CDDP) were added, chemotherapy-sensitive cells underwent apoptosis, while chemotherapy-resistant cells underwent autophagy (Hwang et al., 2020; Lund et al., 2017; Shi et al., 2016).

This research aimed to identify the role of MFAP2 in GC. Here, we first screened out the differentially expressed genes (DEGs), and further determined *FAP*, *INHBA* and *MFAP2* as significant targets in the pathogenesis of GC through enrichment analysis, survival analysis, as well as receiver operating characteristic (ROC) analysis. Finally, we also demonstrated that MFAP2 enhanced CDDP resistance of GC cells by inducing autophagy.

## Materials and methods

### Bioinformatics analysis

The Gene Expression Omnibus (GEO) database (https://www.ncbi.nlm.nih.gov/geo/) was employed to acquire the matrices about GC. The several screening criteria were set as follows: (1) samples with normal controls and GC patients; (2) the number of samples ≥10. Finally, 4 expression matrices were selected for this study, namely GSE54129, GSE65801 (Li et al., 2015), GSE79973 (Jin et al., 2017) and GSE103236 (Necula et al., 2020). By applying The Cancer Genome Atlas (TCGA) database (https://portal.gdc.cancer.gov/), we downloaded the stomach gene expression data (TCGA-STAD) and clinical data (Shao et al., 2021).

When conducting the DEGs analysis, we firstly conversed the ID from the GEO database based on the platform information corresponding to the chip, and then the limma package was used for correction (Ji et al., 2021). The data were grouped into two categories (normal *vs* tumor), and the DEGs were further identified with the standard of —Fold Change—>2, *P* <0.05. For next generation sequencing data of the TCGA database, one gene annotation GTF file was used for ID transformation, and edgeR package was applied to analyze the normal and tumor groups. The DEGs in the TCGA database were screened out based on the same standard as above. The up- and down-regulated genes of the four chips in the GEO database were intersected respectively, and we took the intersection of DEGs in the TCGA and GEO databases. The intersecting DEGs were the final differential genes of GC.

R package was applied for Gene Ontology (GO) and Kyoto Encyclopedia of Genes and Genomes (KEGG) enrichment analysis of the above DEGs (Zhou, Lu & Xiong, 2021). Differential expression boxplots of the DEGs were drawn to verify the correctness of differential expression. Furthermore, survival analysis was carried out according to the survival information from the TCGA database, and Kaplan–Meier curves were drawn (Wang et al., 2021). For genes with significant difference in survival, clinical correlation analysis was further conducted according to the clinical data from the TCGA database. The diagnostic value of the genes was analyzed by a ROC curve, and the area under the curve (AUC) of 3 and 5 years were calculated, respectively (Cheng et al., 2019).

### Cell culture

Procell Life Science&Technology Co., Ltd. (Wuhan, China) sold us human GC cell line AGS. CDDP was purchased from Qilu Pharmaceutical Co., Ltd. (Jinan, China) and dissolved with normal saline. Autophagy inducer rapamycin (Rap) was acquired from Selleck (Shanghai, China). The AGS was selected as drug-sensitive cell line. AGS/CDDP cell line was developed by gradually increasing the drug concentration (0.5, 1.0, 1.5, 2.0, 2.5 and 3.0 µg/mL CDDP, respectively) over a period of 12 months. Before the experiment, the AGS/CDDP were cultured in CDDP-free culture medium for a duration of two weeks (Peng et al., 2020).

### MTT assay

AGS or AGS/CDDP was seeded into 96-well plates for 24 h at 1 ×10^3^ cells per well, and then received various treatments for 48 h. The medium including 0.5 mg/ml MTT reagent was put into the 96-well plates for 4 h. Finally, DMSO was applied to make formazan completely dissolved. The absorbance at 490 nm was recorded by Synergy Neo2 Hybrid Multi-Mode Reader (BioTek, El Segundo, CA, USA) (Lin et al., 2021).

### Western blot

Total protein from the experimental cells was extracted with RIPA lysis buffer and quantified by bicinchoninic acid (BCA) assay. The sodium dodecyl sulfate-polyacrylamide gel electrophoresis (SDS-PAGE) was applied to separate the proteins, and then the separated proteins were transferred to a polyvinylidene fluoride (PVDF) membrane (Millipore Corp., Burlington, MA, USA) (Wang et al., 2019). Then the membrane was blocked with 5% skim milk and incubated with the primary antibody MFAP2 (A10230, 1:2000; ABclone, Wuhan, China), *β*-actin (ab8227, 1:10000; Abcam, Cambridge, UK), LC3II (ab192890, 1:2000; Abcam), cleaved-caspase-3 (ab32042, 1:500; Abcam), cleaved-PARP (ab32064, 1:1000; Abcam), cleaved-caspase-9 (ab32539, 1:1000; Abcam), Beclin 1 (ab207612, 1:1000; Abcam), ATG5 (ab108327, 1:1000; Abcam) and P62 (ab56416, 1:1000; Abcam) at 4 °C overnight and the second antibody (ab7090 or ab47827; Abcam), respectively. Finally, ChemiDoc XRS+ System (Bio-rad, Hercules, CA, USA) was applied to capture chemiluminescence photos.

### Apoptosis assay

The apoptosis analysis was performed through Annexin V-fluorescein isothiocyanate (FITC) / propidium iodide (Annexin V-FITC/PI) staining (Zheng et al., 2017). MultiSciences sold us Annexin V-FITC/PI apoptosis kit (catalog number: 70-AP101-100). AGS or AGS/CDDP was seeded into 6-well plates, and dissociated into single cells by EDTA-free trypsin digestion. After being washed, the cells were re-suspended in 400 µl binding buffer and incubated with 5 µl annexin V-FITC and 10 µl PI for 20 min in the dark. After filtration, utilizing flow cytometer (CytoFLEX; Beckman Coulter, Brea, CA, USA), the apoptosis rate was determined with CytExpert software.

### Cell transfection

AGS or AGS/CDDP was seeded into 6-well plates at 1 ×10^5^ cell/mL and grown to the confluence of 80%. Then, the MFAP2 over-expression plasmid (OE-MFAP2, 4 *μ*g) or MFAP2 siRNA (siMFAP2, 100 pmol) was transfected employing Lipofectamine 2000 reagent (Invitrogen, Waltham, MA, USA) under the guidance of standard protocol. Finally, cells were incubated overnight.

### Statistical analysis

The experimental data were presented graphically utilizing GraphPad Prism 6.0 software and statistically analyzed using SPSS software (version 16.0). All experiments in this study were repeated 3 times independently. Data were indicated as mean ± SD. Comparisons were made between groups employing the Student’s *t*-test, and the analysis of variance (ANOVA) was applied among multiple groups. ^∗^*P* <0.05, ^∗∗^*P* <0.01 and ^∗∗∗^*P* <0.001 were statistically significant.

## Results

In this study, a comprehensive study was carried out to verify the role of MFAP2 through bioinformatics analysis and *in vitro* cell experiment. First of all, DEGs was analyzed in the GEO and TCGA databases. Subsequently, GO enrichment analysis, KEGG enrichment analysis and survival analysis were performed. Furthermore, clinical correlation analysis, survival analysis and ROC analysis were conducted based on the clinicopathological characteristics from TCGA. Finally, we developed the CDDP-resistant cell line (AGS/CDDP), and investigated the effect of MFAP2 on autophagy and CDDP resistance through MTT assay, western blot, and apoptosis assay.

### Database analysis

The GPL type and case information of the GEO datasets were shown in Table 1. There are 235 samples (163 GC samples and 72 non-GC samples), and they are mainly Chinese samples. The clinical characteristics from the TCGA dataset were list in Table 2, including 378 GC cases.

**Table 1:** Information of GEO series datasets.

ID	Type	Country	Case	
			GC	non-GC
GSE54129	GPL570	China	111	21
GSE65801	GPL14550	China	32	32
GSE79973	GPL570	China	10	10
GSE103236	GPL4133	Romania	10	9

**Table 2:** The clinicopathological features of patients in TCGA.

Clinicopathological parameters		Cases (*n* = 378)
Age	<=65	172
	>65	206
Gender	FEMALE	141
	MALE	237
Grade	G1	8
	G2	127
	G3	243
Stage	Stage I	48
	Stage II	120
	Stage III	169
	Stage IV	41
T	T1	17
	T2	76
	T3	180
	T4	105
M	M0	351
	M1	27
N	N0	120
	N1	100
	N2	79
	N3	79

### Differential expression analysis

**Figure 1:**
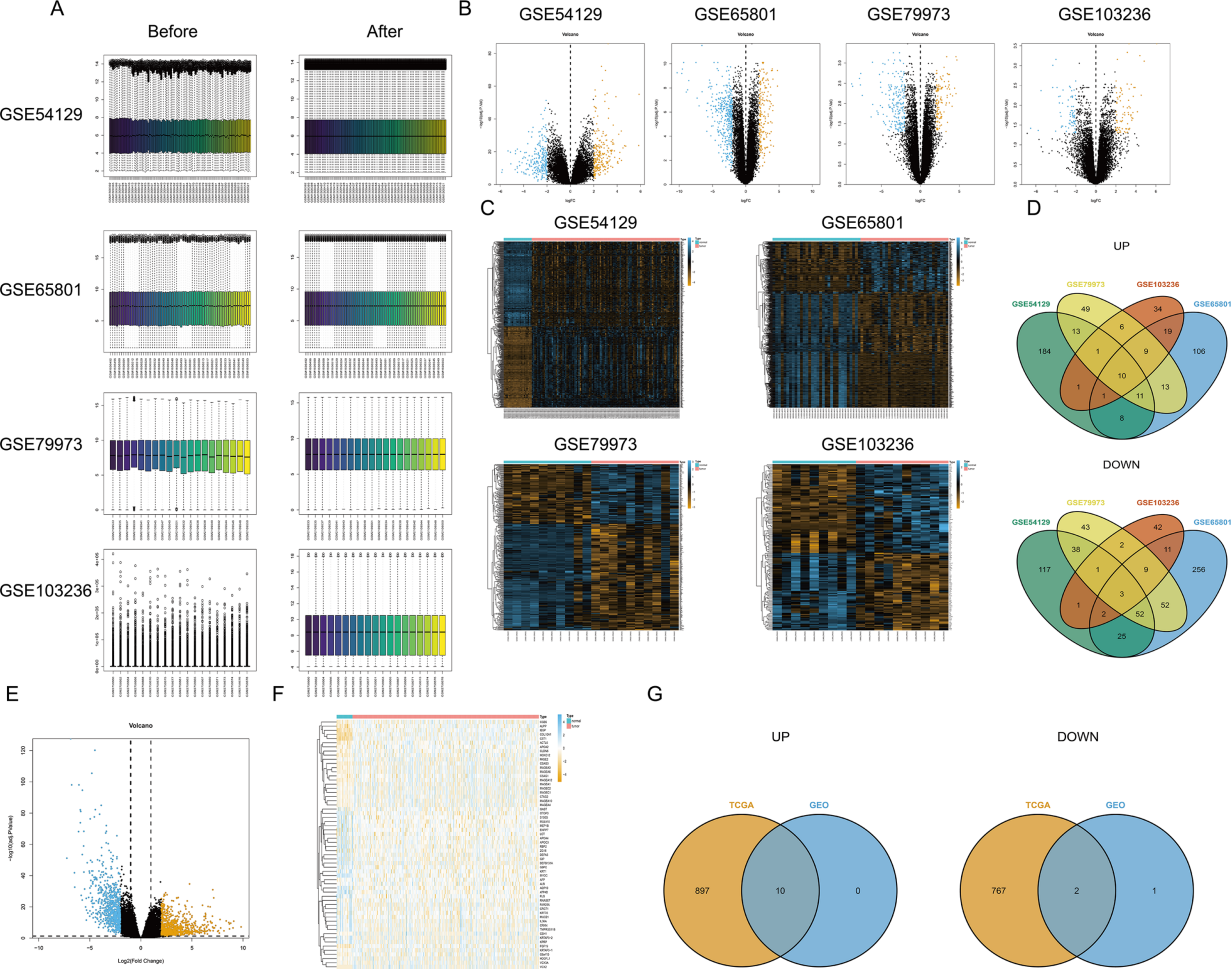
Differential expression analysis. (A) Data normalization; (B) volcano map of DEGs (four GEO datasets); (C) heat map of DEGs (four GEO datasets); (D) intersection of DEGs obtained from four GEO datasets; (E) volcano map of DEGs (TCGA database); (F) heat map of DEGs (TCGA database); (G) the intersection of DEGs based on the TCGA database and the GEO database.

First, the limma package was applied to make the four chips normalized (Fig. 1A). The right graphs were normalized. Then, the DEGs were screened out in GC and non-GC groups according to the above standard. Volcano maps (Fig. 1B) and heat maps (Fig. 1C) were drawn for the results of the four chips. The intersection of up- or down-regulated genes obtained from the GEO database was taken respectively, and there were 10 up-regulated genes (BGN, INHBA, CTHRC1, SULF1, THBS2, FAP, MFAP2, SFRP4, SPP1, and COL10A1) and three down-regulated genes (MAL, GIF, and ATP4B) (Fig. 1D). Based on TCGA-STAD, the DEGs were screened out by edgeR package, and the volcano map (Fig. 1E) and heat map (Fig. 1F) were plotted. Finally, the intersection of DEGs in the TCGA database and the GEO database was taken, and a total of 10 up-regulated genes (BGN, INHBA, CTHRC1, SULF1, THBS2, FAP, MFAP2, SFRP4, SPP1, and COL10A1) and two down-regulated genes (MAL and ATP4B) were acquired (Fig. 1G).

### Functional pathway enrichment analysis

**Figure 2:**
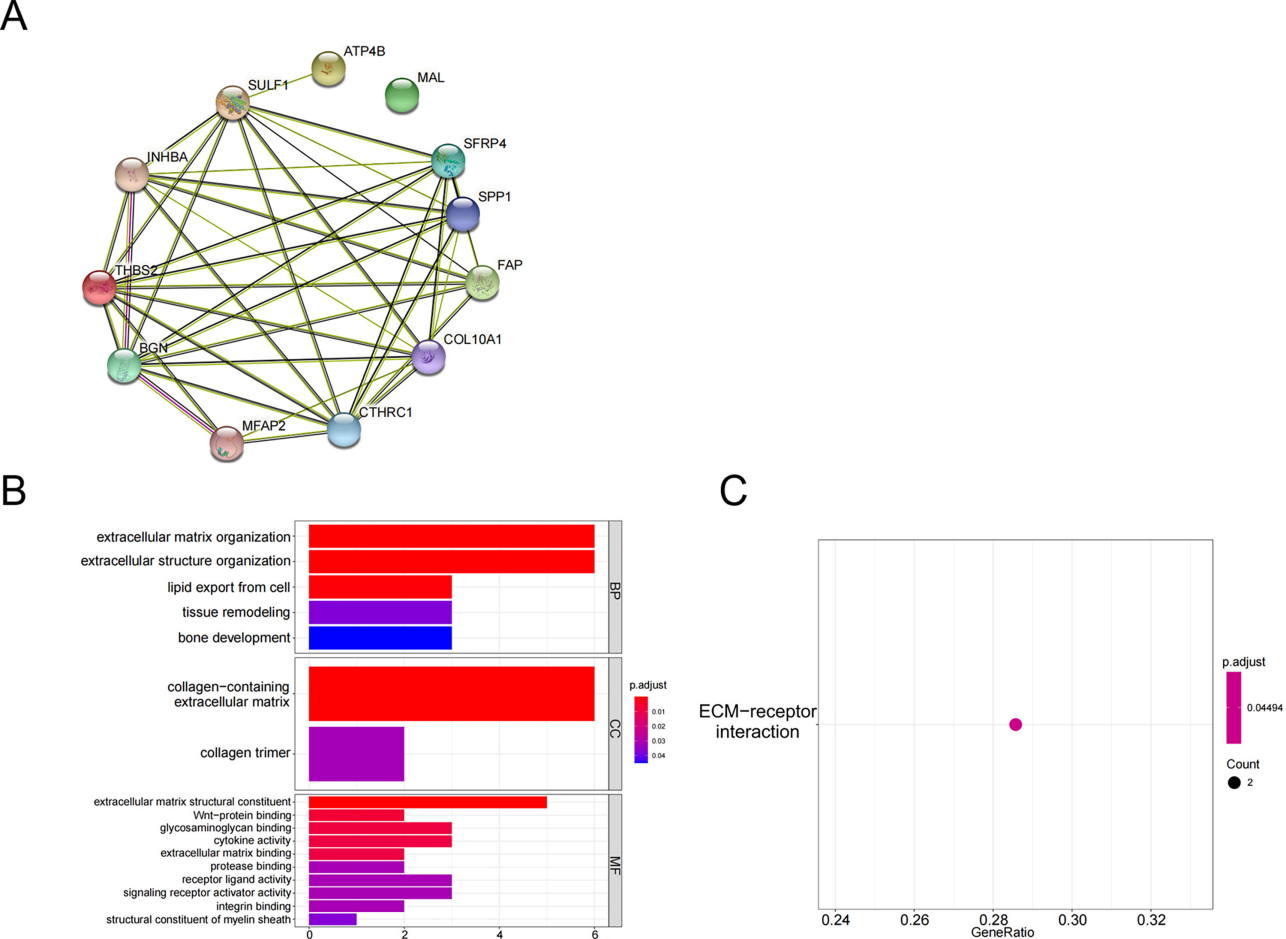
Functional pathways analysis. (A) PPI network construction; (B) GO analysis; (C) KEGG analysis.

The above 10 up-regulated genes and two down-regulated genes were used to establish a protein-protein interaction (PPI) network (Fig. 2A). GO enrichment analysis (Fig. 2B, Table 3) and KEGG enrichment analysis (Fig. 2C) were performed (*P* <0.05 and *P*.adjust <0.05). These genes were enriched in the GO pathways of extracellular matrix organization, extracellular structure organization, collagen-containing extracellular matrix, extracellular matrix structural constituent, and wnt-protein binding. KEGG enrichment analysis showed that THBS2 and SPP1 were enriched in the pathway of extracellular matrix (ECM)-receptor interaction.

**Table 3:** Genes in GO pathways.

**GO pathways description**	**Genes**
extracellular matrix organization	BGN/SULF1/FAP/MFAP2/SPP1/COL10A1
extracellular structure organization	BGN/SULF1/FAP/MFAP2/SPP1/COL10A1
lipid export from cell	INHBA/SPP1/MIF
tissue remodeling	BGN/CTHRC1/SPP1
bone development	BGN/SULF1/SFRP4
collagen-containing extracellular matrix	BGN/CTHRC1/SULF1/THBS2/MFAP2/COL10A1
collagen trimer	CTHRC1/COL10A1
extracellular matrix structural constituent	BGN/CTHRC1/THBS2/MFAP2/COL10A1
Wnt-protein binding	CTHRC1/SFRP4
glycosaminoglycan binding	BGN/SULF1/THBS2
cytokine activity	INHBA/SPP1/MIF
extracellular matrix binding	BGN/SPP1
protease binding	FAP/MIF
receptor ligand activity	INHBA/SPP1/MIF
signaling receptor activator activity	INHBA/SPP1/MIF
integrin binding	FAP/SPP1
structural constituent of myelin sheath	MAL
sodium:potassium-exchanging ATPase activity	ATP4B
potassium transmembrane transporter activity, phosphorylative mechanism	ATP4B
activin receptor binding	INHBA
sodium transmembrane transporter activity, phosphorylative mechanism	ATP4B
arylsulfatase activity	SULF1
intramolecular oxidoreductase activity, transposing C=C bonds	MIF
sulfuric ester hydrolase activity	SULF1
proton-exporting ATPase activity, phosphorylative mechanism	ATP4B
peptidase activator activity involved in apoptotic process	MAL
extracellular matrix structural constituent conferring compression resistance	BGN
transmembrane receptor protein serine/threonine kinase binding	INHBA
ATPase activator activity	ATP4B

### Boxplot analysis of gene expression

**Figure 3:**
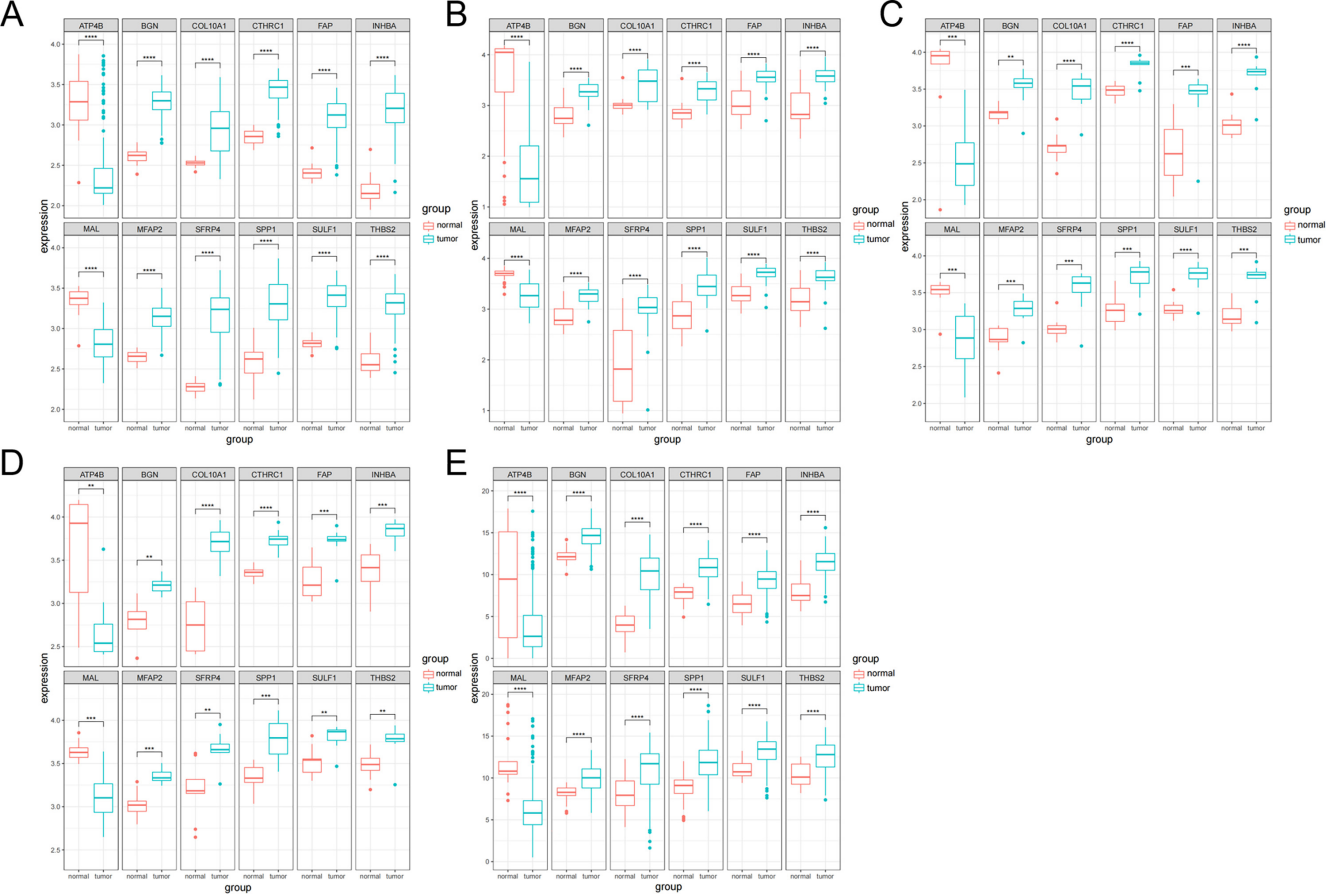
Boxplot analysis of differential expression. (A) GSE54129; (B) GSE65801; (C) GSE79973; (D) GSE103236; (E) TCGA database.

The expression analysis of the 12 genes was performed. The boxplots were plotted in different datasets: GSE54129 (Fig. 3A), GSE65801 (Fig. 3B), GSE79973 (Fig. 3C), GSE103236 (Fig. 3D) and TCGA (Fig. 3E). It showed that *ATP4B* and *MAL* were downregulated in GC, while *BGN*, *COL10A1*, *CTHRC1*, *FAP*, *INHBA*, *MFAP2*, *SFRP4*, *SPP1*, *SULF1* and *THBS2* were upregulated in GC.

### Survival analysis

**Figure 4:**
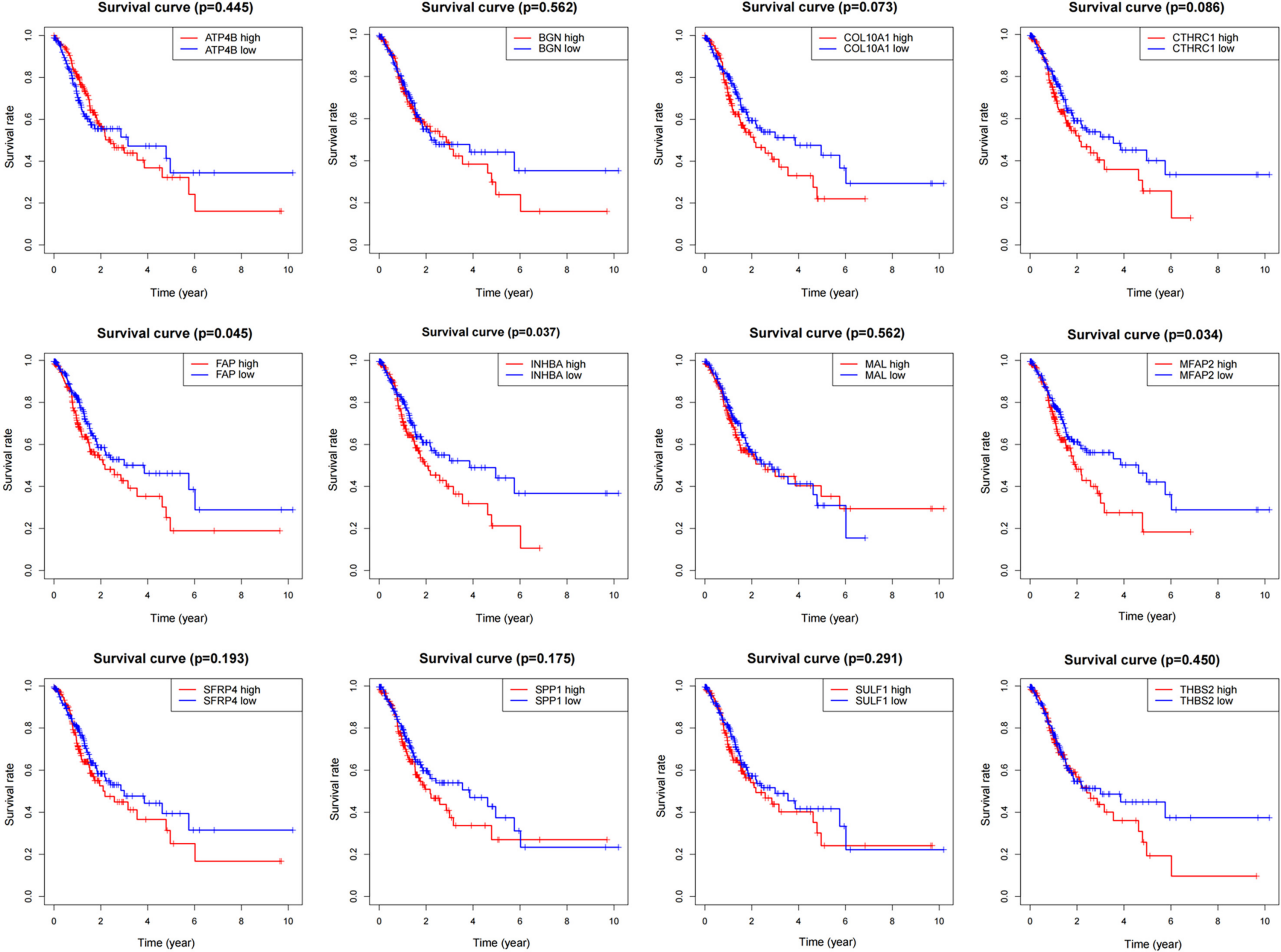
Survival analysis of DEGs.

To determine the influence of the above 12 genes on prognosis, survival analysis was performed based on the TCGA data (Fig. 4). According to the results, *FAP*, *INHBA* and *MFAP2* had significant effects on survival (*P* = 0.045, 0.037, 0.034), so they were used for subsequent analysis.

### Clinical correlation analysis

**Figure 5:**
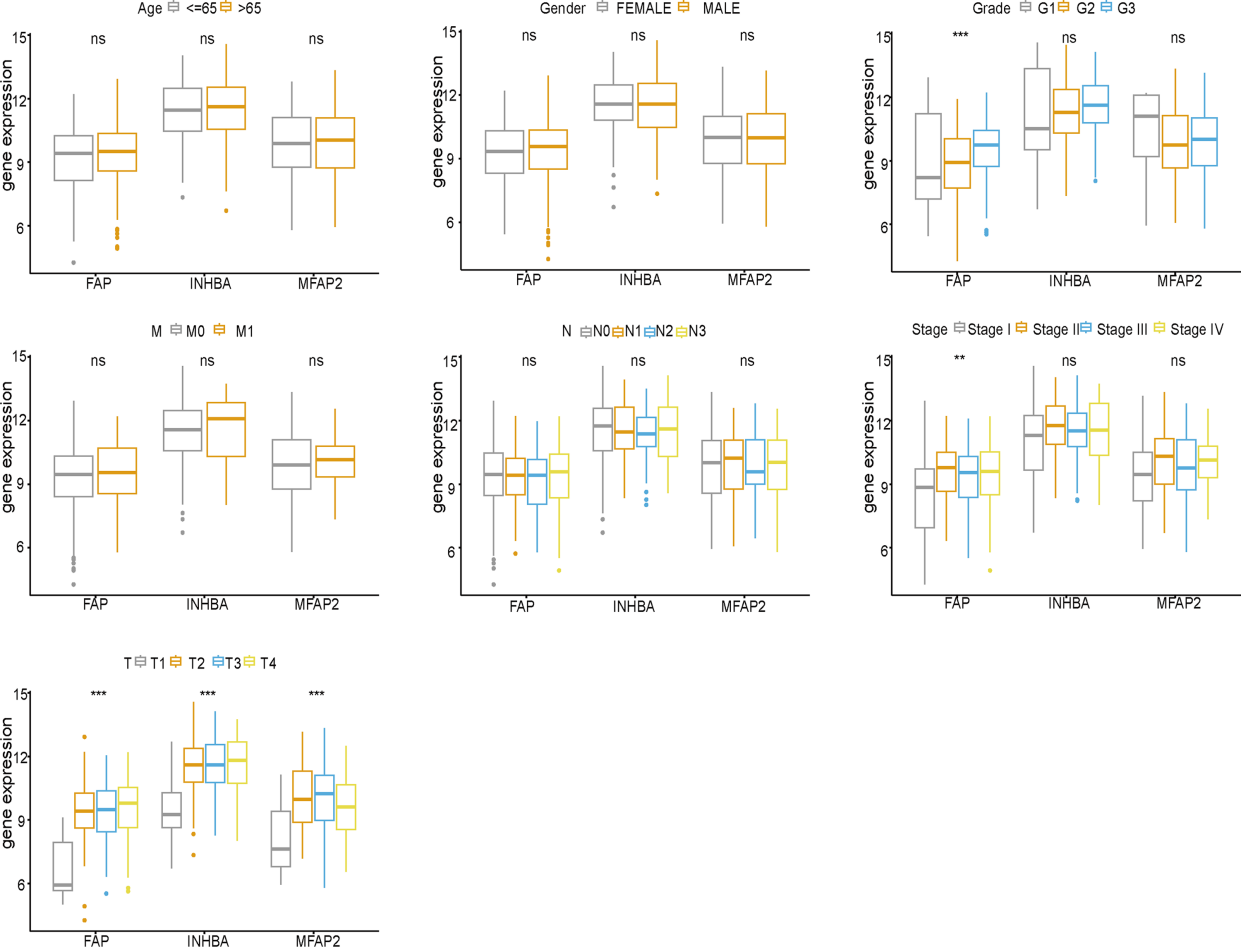
Clinical correlation analysis of *FAP*, *INHBA* and *MFAP2*.

*FAP*, *INHBA* and *MFAP2* were subjected to clinical correlation analysis according to the TCGA clinical data (Fig. 5). The clinical information of samples was shown in Table 2. According to the correlation analysis results, *FAP* was correlated with Grade, Stage and T stage, and both *INHBA* and *MFAP2* were related to T stage.

### ROC analysis

**Figure 6:**
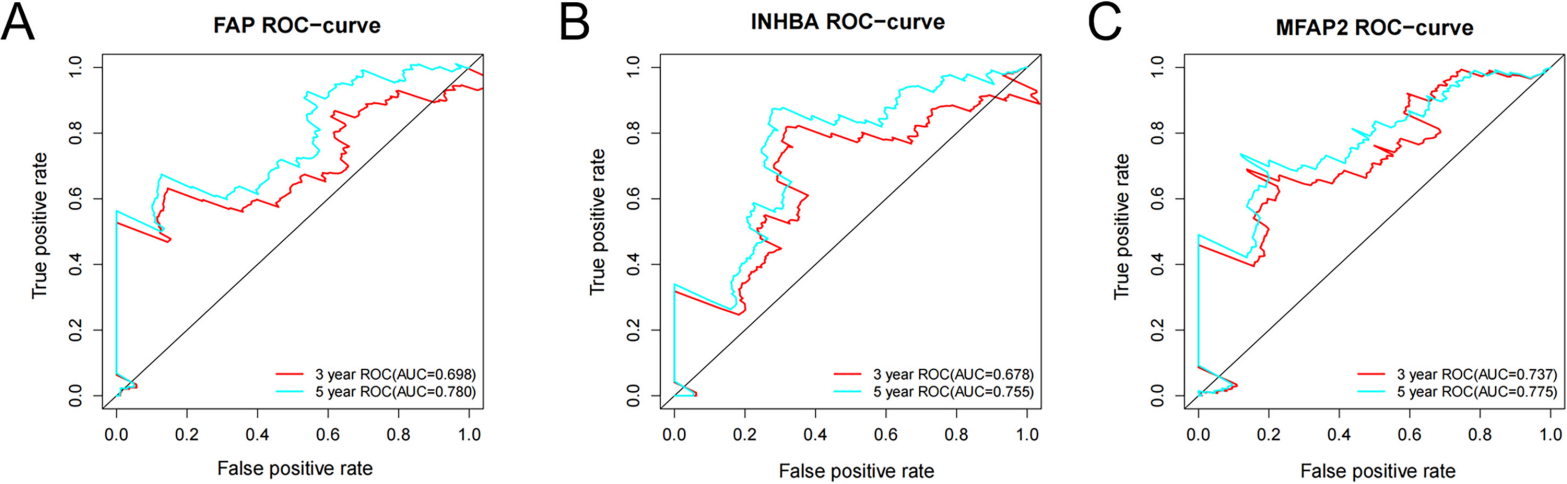
ROC analysis of *FAP*, *INHBA* and *MFAP2*. (A) ROC analysis of *FAP*; (B) ROC analysis of *INHBA*; (C) ROC analysis of *MFAP2*.

ROC analysis was conducted on the above three genes based on the survival time and survival status from the TCGA. The ROC-curves of 3-year and 5-year survival prediction were plotted respectively. According to the outcomes, the accuracy of 5-year survival prediction was more than 0.75, and *FAP* (Fig. 6A), *INHBA* (Fig. 6B) and *MFAP2* (Fig. 6C) can be good diagnostic factors of GC. Among the three genes, the AUC of *MFAP2* in the ROC-curves of 3-year and 5-year survival prediction were all more than 0.7. In this study, *MFAP2* was selected for subsequent research.

### MFAP2 was upregulated in AGS/CDDP cells

**Figure 7:**
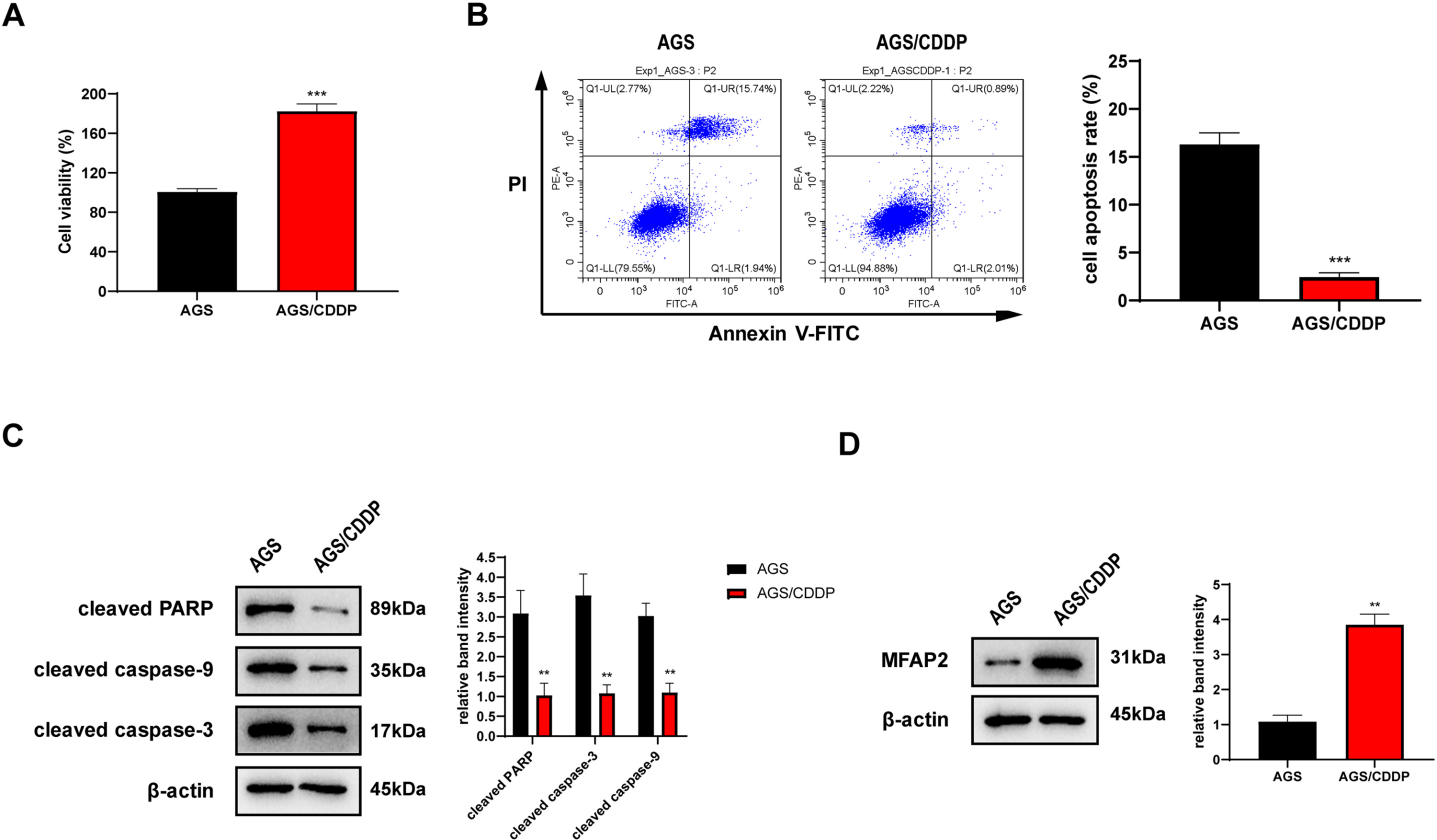
Cell differences between AGS and AGS/CDDP. (A) The cell viability of AGS and AGS/CDDP detected by MTT (*P* < 0.0001); (B) Apoptosis rate measured by Annexin V-FITC/PI assay (*P* < 0.0001); (C) cleaved PARP, cleaved caspase-9, and cleaved caspase-3 displayed by western blot (*P* = 0.0055, 0.0011, 0.0018); (D) MFAP2 detected by western blot (*P* = 0.0023).

AGS/CDDP was established by concentration gradient induction. 3 µg/ml CDDP was supplied into the medium of AGS and AGS/CDDP cells. MTT assay showed that the cell viability of AGS/CDDP was significantly higher than AGS (Fig. 7A). Annexin V-FITC/PI staining suggested that the cell apoptosis rate of AGS/CDDP was significantly reduced (Fig. 7B). Pro-apoptotic protein cleaved PARP, cleaved caspase-3, and cleaved caspase-9 were downregulated in AGS/CDDP (Fig. 7C). The results of MTT and cell apoptosis analysis showed that drug-resistant cell line AGS/CDDP was obtained. Furthermore, western blot suggested that MFAP2 was upregulated in AGS/CDDP cells in contrast with AGS cells (Fig. 7D).

### Effect of MFAP2 on CDDP sensitivity of AGS

**Figure 8:**
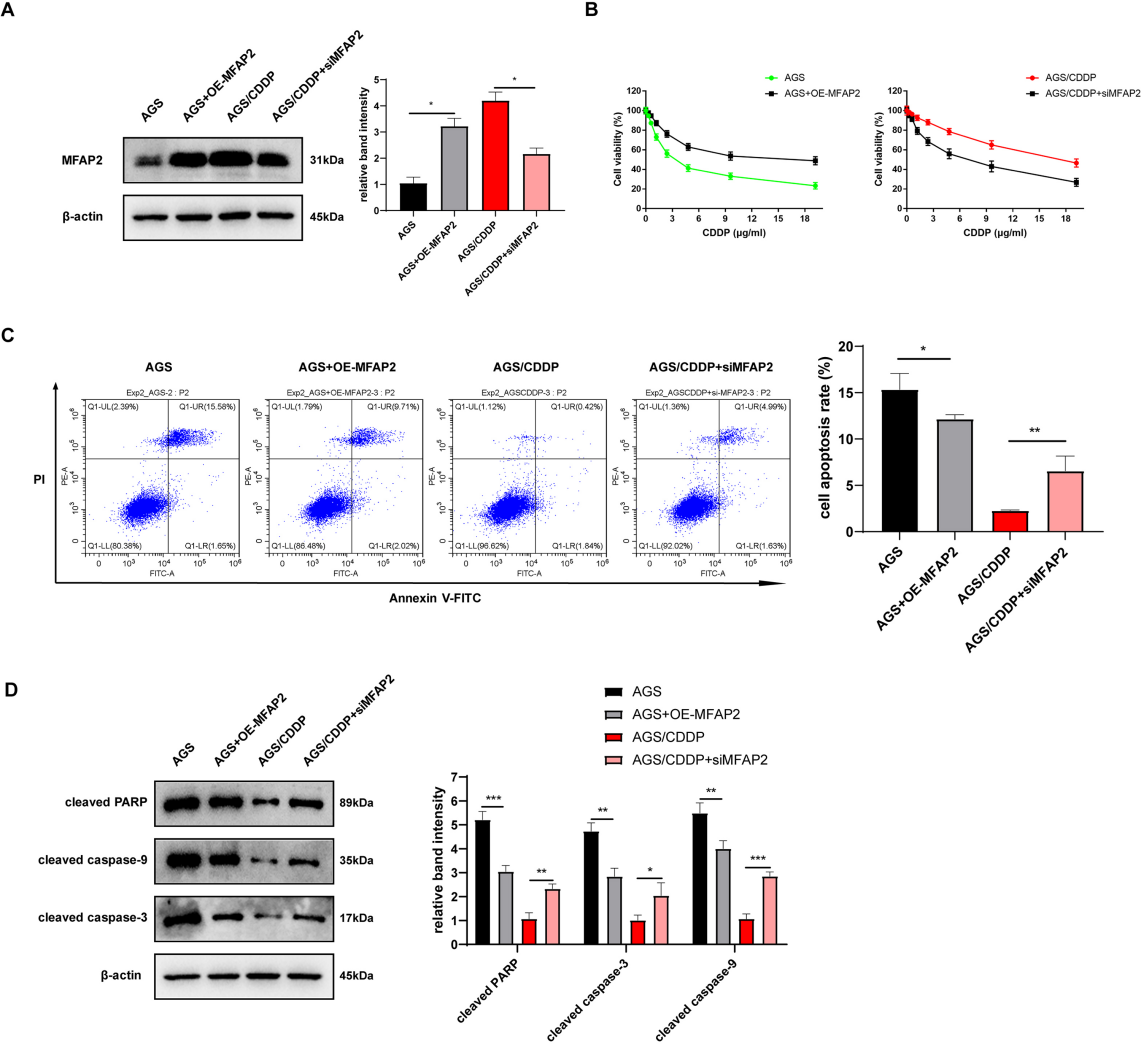
Effect of MFAP2 on CDDP sensitivity of AGS. (A) The expression of MFAP2 (compared with AGS, *P* = 0.0113; compared with AGS/CDDP, *P* = 0.0136); (B) the cell viability at different concentrations of CDDP (IC50); (C) cells were cultured at 3 µg/mL CDDP for 24 h, and Annexin V-FITC/PI staining assay was applied to observe apoptosis rate (compared with AGS, *P* = 0.0375; compared with AGS/CDDP, *P* = 0.0098); (D) cells were cultured at 3 µg/mL CDDP for 48 h, and the content of pro-apoptotic proteins (cleaved PARP, cleaved caspase-3, and cleaved caspase-9) was detected (compared with AGS, *P* = 0.0009, 0.0025, 0.0089; compared with AGS/CDDP, *P* = 0.0023, 0.035, 0.0003).

To further explore the action of MFAP2 on CDDP sensitivity, we transfected MFAP2 overexpressed plasmid (OE-MFAP2) and siMFAP2 in AGS and AGS/CDDP cells, respectively. Western blot results showed that MFAP2 was upregulated in the AGS+OE-MFAP2 in contrast with the AGS, and apparently downregulated in the AGS/CDDP+siMFAP2 compared to the AGS/CDDP (Fig. 8A). In comparison with the AGS, the IC50 was increased (17.79 *vs* 3.04 µg/mL, Fig. 8B), the cell apoptosis was reduced (Fig. 8C), and the sensitivity to CDDP were decreased in the AGS+OE-MFAP2 group. Additionally, in contrast with the AGS/CDDP, the AGS/CDDP+siMFAP2 group showed a lower IC50 (5.47 *vs* 18.21 µg/mL, Fig. 8B), a higher level of cell apoptosis (Fig. 8C), and an increased sensitivity to CDDP. Western blot was applied to detect the expression of pro-apoptotic protein, the result of which was consistent with that of Annexin V-FITC/PI staining (Fig. 8D). In this section, we confirmed that MFAP2 can regulate the CDDP sensitivity of AGS.

### Effect of MFAP2 on the autophagy in AGS

**Figure 9:**
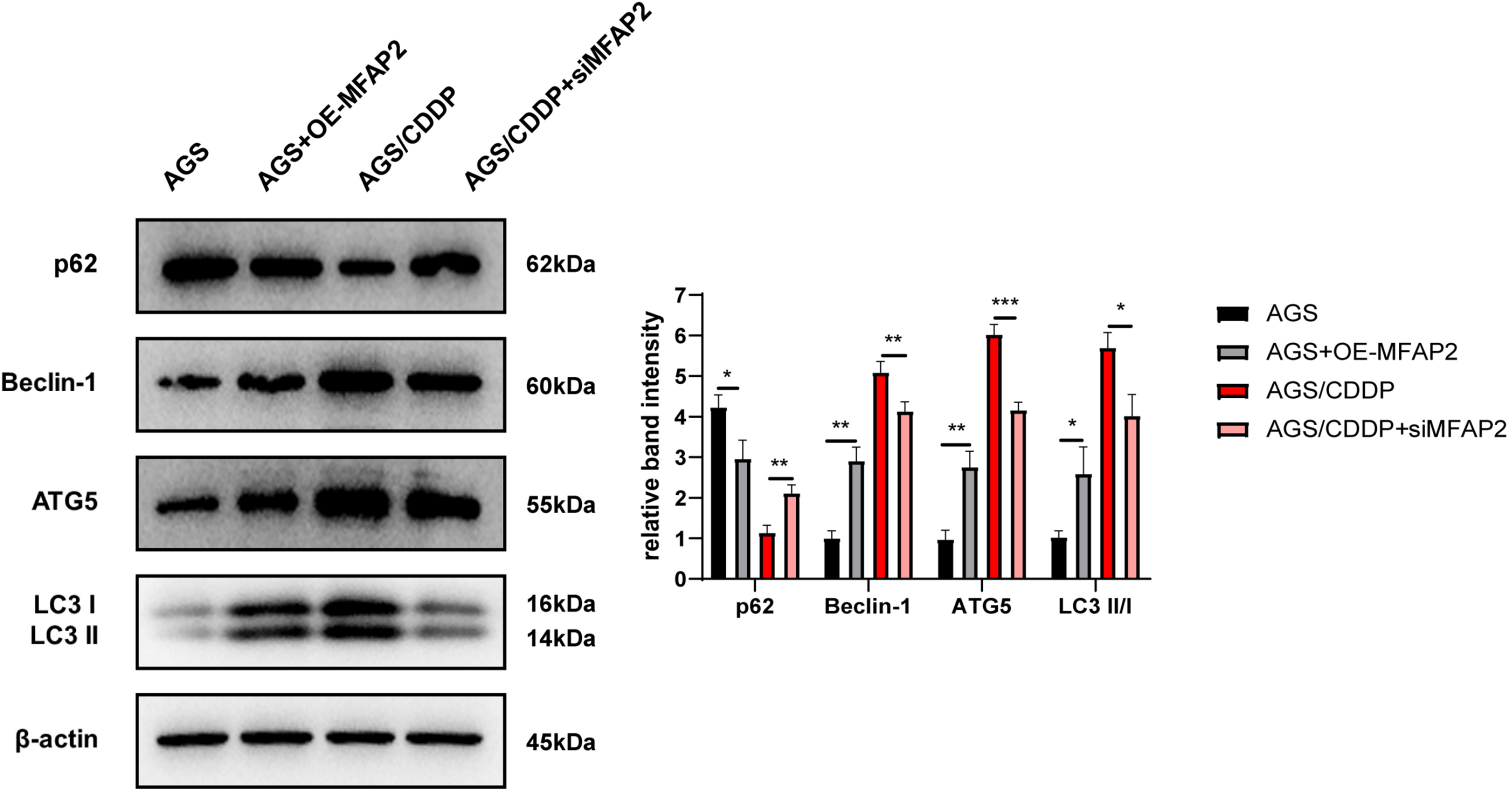
The expression of p62, Beclin-1, ATG5 and LC3 I/II. Compared with AGS, *P* = 0.016, 0. 0011, 0.0023, 0.016; compared with AGS/CDDP, *P* = 0.0034, 0.0092, 0.0005, 0.0106.

To explore the mechanism of MFAP2 regulating CDDP sensitivity, the effect of MFAP2 on cell autophagy was further studied. After cells were cultured at 3 µg/mL CDDP for 48 h, the expression of autophagy-related proteins was determined (Fig. 9). The results of western blot displayed that compared with the AGS group, p62 in the AGS+OE-MFAP2 group was downregulated, while Beclin-1, ATG5 and LC3 II were upregulated, and autophagy was enhanced. Compared to the AGS/CDDP, the expression of p62 in the AGS/CDDP+siMFAP2 was upregulated, while Beclin-1, ATG5 and LC3 II expression were downregulated, suggesting that autophagy was decreased. We preliminarily confirmed that MFAP2 plays an important role on autophagy on the basis of these results.

### MFAP2 enhanced CDDP resistance by promoting autophagy

**Figure 10:**
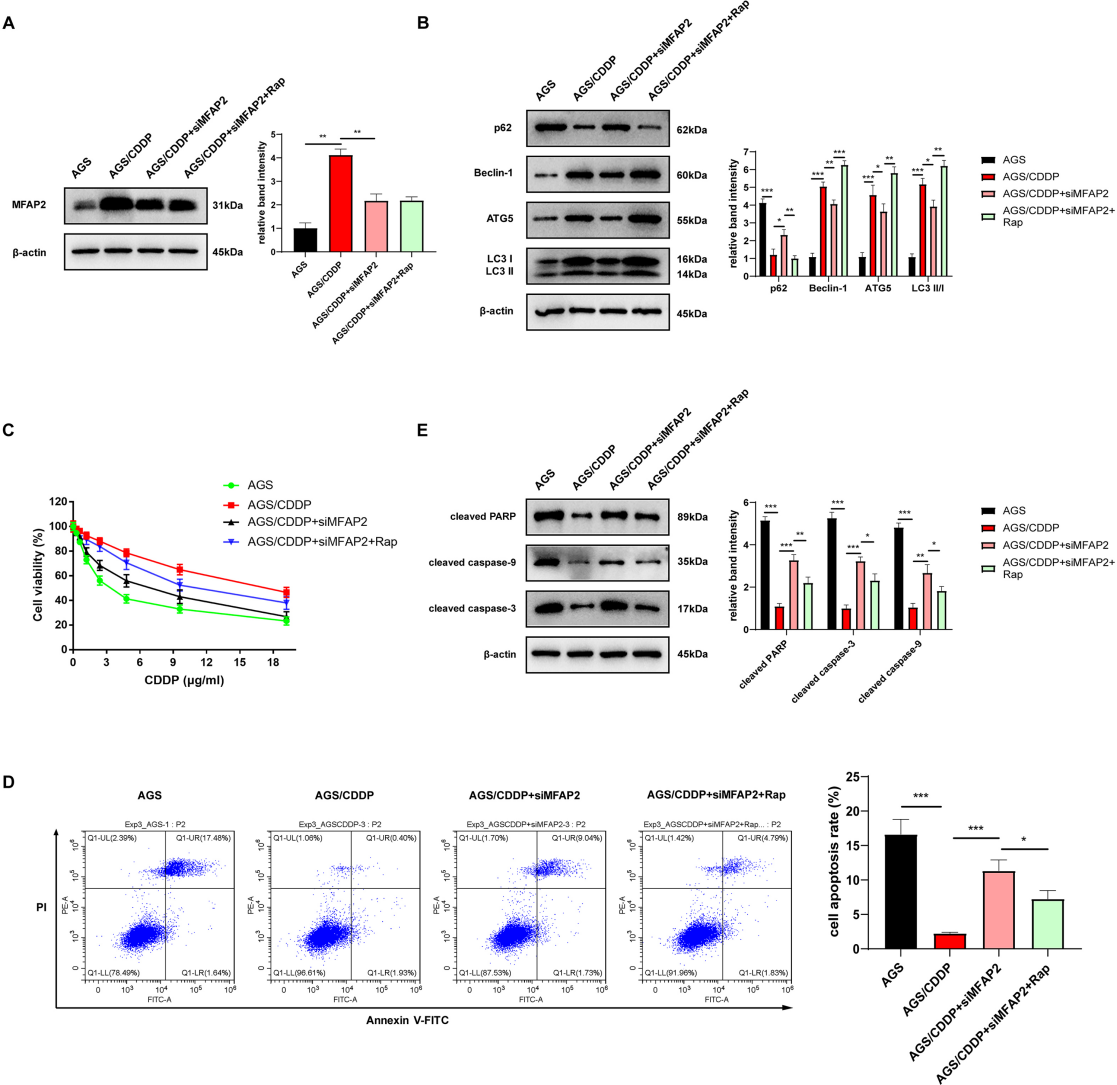
MFAP2 promoted autophagy and enhanced CDDP resistance of AGS. (A) The expression of MFAP2 (compared with AGS, *P* = 0.0047; compared with AGS/CDDP, *P* = 0.0036); (B) the protein content of p62, Beclin-1, ATG5 and LC3 I/II (compared with AGS, *P* = 0.0001, 0.00002, 0.0005, 0.00004; compared with AGS/CDDP, *P* = 0.011, 0.007, 0.0442, 0.011; compared with AGS/CDDP+siMFAP2, *P* = 0.0021, 0.0003, 0.0023, 0.0011); (C) the cell viability at different concentrations of CDDP (IC50); (D) the cell apoptosis after treatment with 3 µg/ml CDDP (compared with AGS, *P* = 0.0003; compared to AGS/CDDP, *P* = 0.0005; compared to AGS/CDDP+siMFAP2, *P* = 0.0227); (E) the protein content of cleaved PARP, cleaved caspase-3, and cleaved caspase-9 in the cells treated with 3 µg/ml CDDP (compared with AGS, *P* < 0.0001; compared to AGS/CDDP, *P* = 0.0002, 0.0001, 0.0031; compared with AGS/CDDP+siMFAP2, *P* = 0.0081, 0.012, 0.029).

To further demonstrate the enhancement of MFAP2 on CDDP resistance by promoting autophagy, the cells were grouped as follows: AGS, AGS/CDDP, AGS/CDDP+siMFAP2, and AGS/CDDP+siMFAP2+Rap (100 nM, 24h). Western blot results suggested that the protein content of MFAP2 was the highest in the AGS/CDDP, and MFAP2 was significantly downregulated in the AGS/CDDP+siMFAP2 and AGS/CDDP+siMFAP2+Rap groups after the transfection with siMFAP2 (Fig. 10A). Compared with the AGS group, the level of autophagy (Fig. 10B) and the CDDP resistance (Fig. 10C–E) were increased in the AGS/CDDP group. After MFAP2 expression was down-regulated, autophagy in the AGS/CDDP+siMFAP2 was weakened (Fig. 10B), and the sensitivity to CDDP was enhanced (Figs. 10C–10E). Further, autophagy was enhanced in the AGS/CDDP+siMFAP2+Rap group (Fig. 10B), and the resistance to CDDP was increased (Figs. 10C–10E) compared with the AGS/CDDP+siMFAP2 group. Overall, we further identified that MFAP2 enhanced CDDP resistance by promoting autophagy.

## Discussion

GC is a common malignant tumor all over the world. Despite substantial progress on the medical diagnosis and treatment of GC, its prognosis is still very poor (Rugge et al., 2020). Therefore, searching for a novel effective therapeutic target may help meliorate the therapeutic effect of GC patients.

The development of bioinformatics is intimately connected to the medical treatment of various diseases, and much more meaningful therapeutic targets were explored by researchers by analyzing and predicting the characteristics of diseases through big data, and the progress of medicine was promoted as well. In this study, through DEGs analysis, functional enrichment analysis, survival analysis, clinical correlation analysis, ROC analysis and etc., it’s confirmed that *FAP*, *INHBA* and *MFAP2* might have good diagnostic values for GC. Consistent with our researches, MFAP2 has also been found by previous studies to play an essential part in GC, and its importance has been demonstrated in the progression of GC (Yao et al., 2020). However, the role of MFAP2 on drug resistance is still unknown, and it has also received great attention.

Drug resistance of GC cells, notably chemotherapeutic drugs resistance, is one of the biggest limiting factors for targeted anti-cancer therapy. Multi-drug resistance, commonly happened in the treatment of GC, reduces the effectiveness or even causes the ineffectiveness of therapies (Wang & Yuan, 2016). CDDP is a commonly used anticancer drug, and its best-characterized mode of action contains the DNA-damage response and mitochondrial apoptosis. However, CDDP-stimulated lesions bring about DNA damage which can be recognized by various repair means (including nucleotide excision repair and mismatch repair system). Moreover, certain drug transporters and ATPases would mediate CDDP resistance to some degree by increasing CDDP export (Galluzzi et al., 2012). In the current research, we investigated the autophagy-related mechanisms in drug-resistant tumors. Studies have stated that the induction of autophagy contribute to cancer cell progression, and help achieve drug tolerance in anticancer therapy (Jiang et al., 2019). It was found that DNA damage or misfolded proteins induced by CDDP upregulates the level of autophagy (Basu & Krishnamurthy, 2010; Kouroku et al., 2007). Therefore, autophagy is of crucial importance to the treatment of drug-resistant tumors.

In an effort to explore the internal mechanism of drug resistance, we firstly constructed a drug-resistant cell line, namely AGS/CDDP, by gradually increasing the concentration of CDDP. We spotted that the protein content of MFAP2 in AGS/CDDP cells tended to increase, which was consistent with Sun’s study (Sun et al., 2020). For the sake of the function exploration of MFAP2 in drug resistance, we investigated the effect of MFAP2 on CDDP sensitivity, through upregulating or downregulating MFAP2 in AGS or AGS/CDDP cells, respectively. We found that when upregulating the level of MFAP2 in AGS cells, the IC50 was increased, while the level of cell apoptosis and the CDDP sensitivity was decreased. However, reducing the expression of MFAP2 in AGS/CDDP cells reversed the above results. The relationship between autophagy and drug-resistant tumors was mentioned above. Therefore, we also investigated the action of autophagy on AGS/CDDP, and found that autophagy was strengthened in AGS/CDDP, while MFAP2-knockdown caused a weakened autophagy by measuring the protein content of autophagy markers (p62, Beclin-1, ATG5 and LC3 I/II). Finally, we reconfirmed that MFAP2 enhanced CDDP resistance of AGS by promoting autophagy, based on treatment with autophagy inducer Rap.

This research demonstrated that MFAP2 enhanced CDDP resistance by inducing autophagy in GC cells. However, there are also some drawbacks. For example, we have not been able to discuss the expression of MFAP2 in drug-resistant patients. In addition, the other two genes we have screened can be further researched, which is what we are focusing on. Finally, the missing data *in vivo* is an important defect in this article. We will take an in-depth study in the future.

## Conclusion

MFAP2 has been confirmed in this study for the first time as a potentially important prognostic marker in GC, and it was revealed that MFAP2 upregulation could promote autophagy in AGS/CDDP. These results demonstrate that autophagy is important for drug-resistant of cancer cells, and MFAP2 induces autophagy to enhance the resistance to CDDP, which offer fresh insights for the chemotherapy treatment of GC.

##  Supplemental Information

10.7717/peerj.15441/supp-1 Supplemental Information 1 Raw data

## Abbreviations

GC: gastric cancer
MFAP2: Microfibril Associated Protein 2
DEGs: Differential Expressed Genes
GO: Gene ontology
KEGG: Kyoto Encyclopedia of Genes and Genomes
ROC: Receiver Operating Characteristic Curve
FAP: Fibroblast Activation Protein
INHBA: Inhibin Subunit Beta A

## Additional Information and Declarations

### Data Availability

The following information was supplied regarding data availability: The gene expressions are available at GEO: GSE54129, GSE65801, GSE79973 and GSE103236. The data from The Cancer Genome Atlas (TCGA) database is available at figshare: Zhang, Rong-Gui (2023): The specific data used from TCGA.zip. figshare. Dataset. https://doi.org/10.6084/m9.figshare.22820897.v2.
