# Supplementary figures and images for: MFAP2 enhances cisplatin resistance in gastric cancer cells by regulating autophagy (part 2 of 2)
Source: PeerJ. 2023 Jun 7;11:e15441. doi: 10.7717/peerj.15441 (PMC10257393; doi:10.7717/peerj.15441)

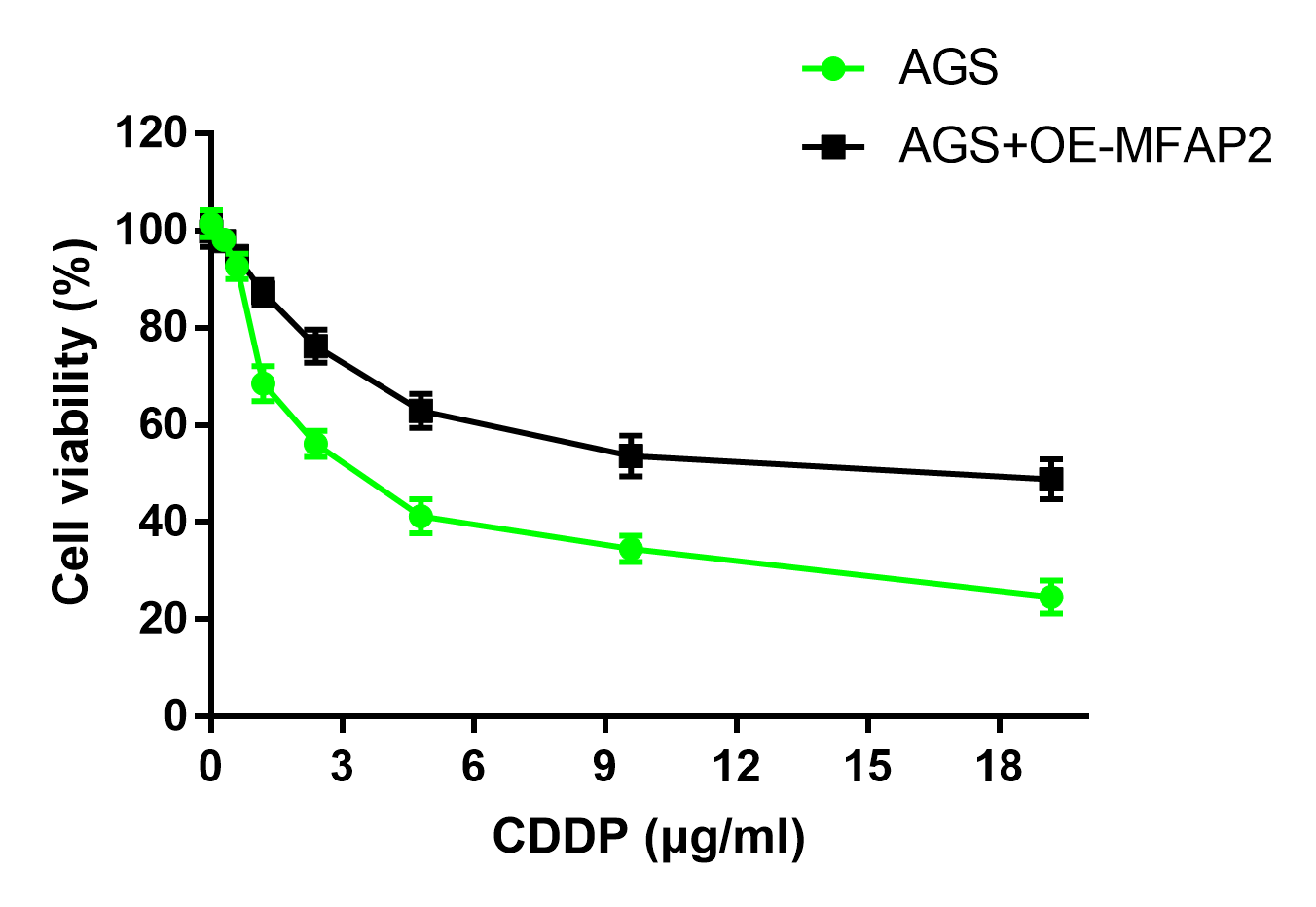

Supplement: Supplemental Information 1 [file peerj-11-15441-s001.zip › Raw data submitted/statistical analysis/Fig. 8B-1.png]

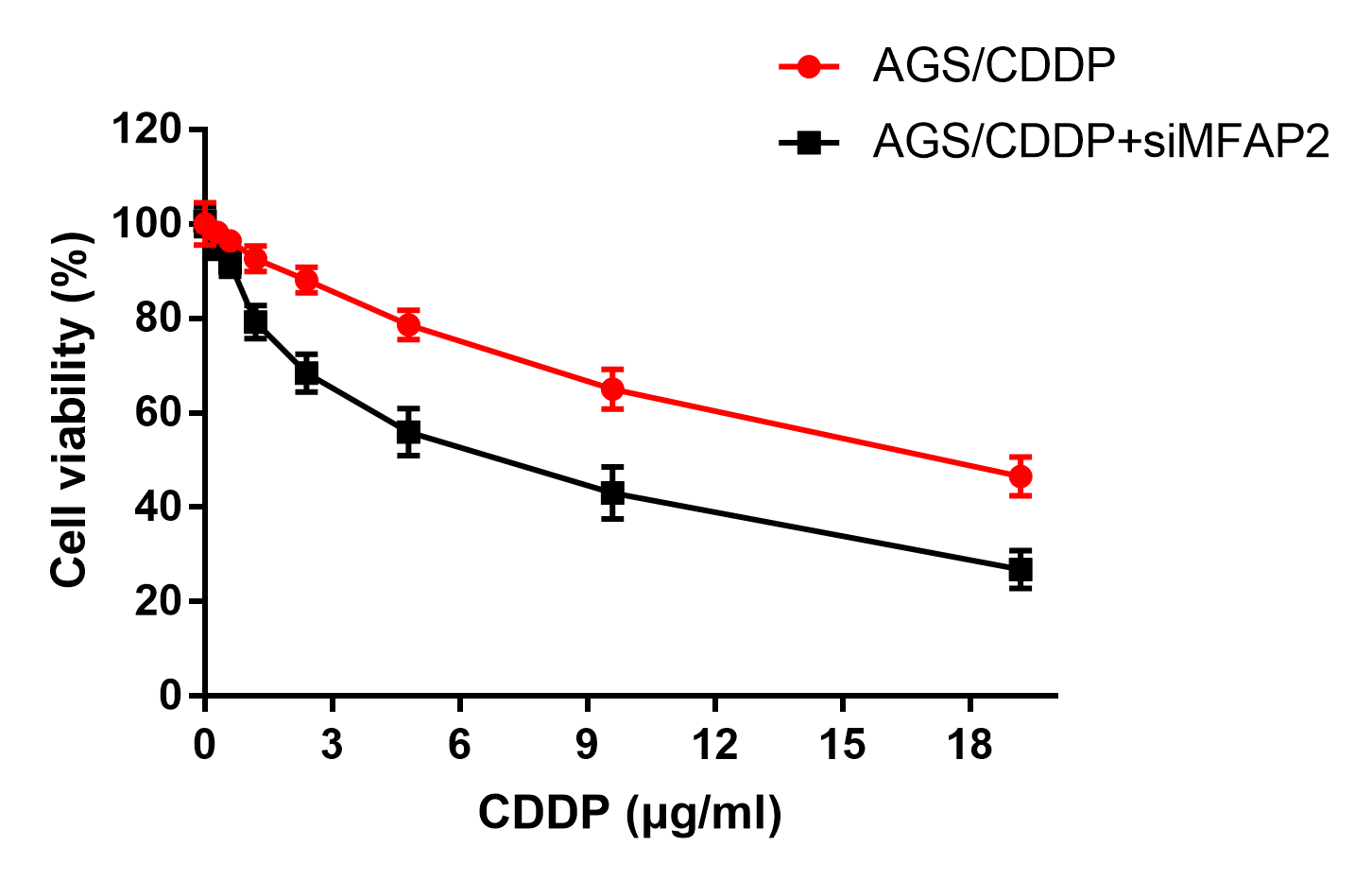

Supplement: Supplemental Information 1 [file peerj-11-15441-s001.zip › Raw data submitted/statistical analysis/Fig. 8B-2.png]

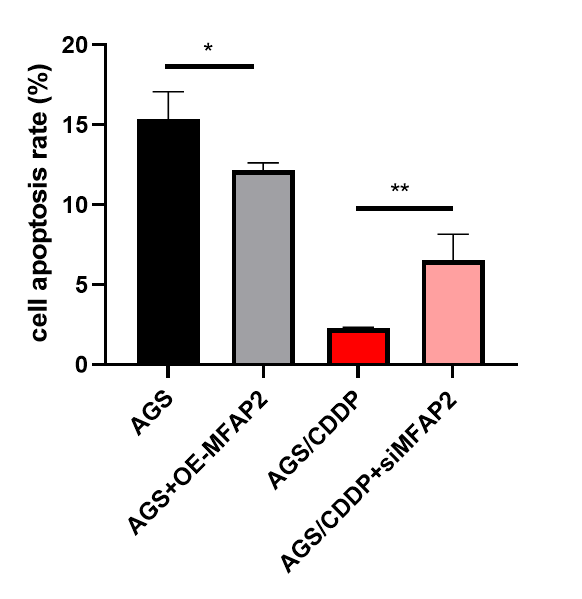

Supplement: Supplemental Information 1 [file peerj-11-15441-s001.zip › Raw data submitted/statistical analysis/Fig. 8C.png]

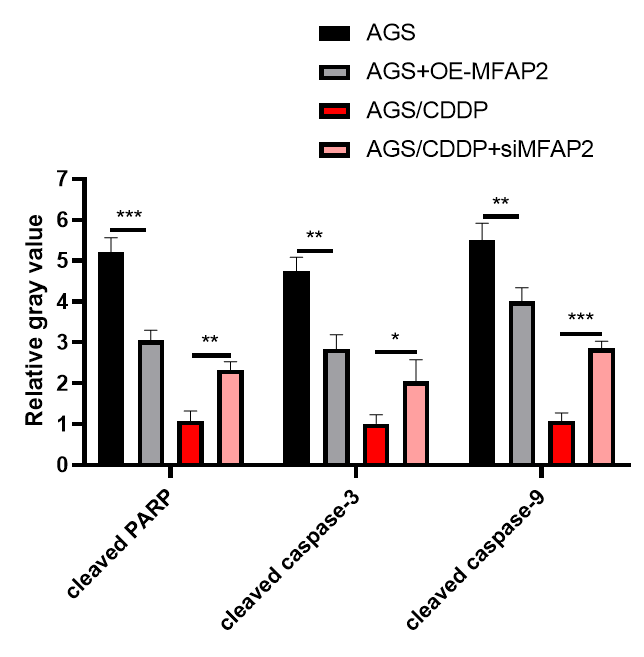

Supplement: Supplemental Information 1 [file peerj-11-15441-s001.zip › Raw data submitted/statistical analysis/Fig. 8D.png]

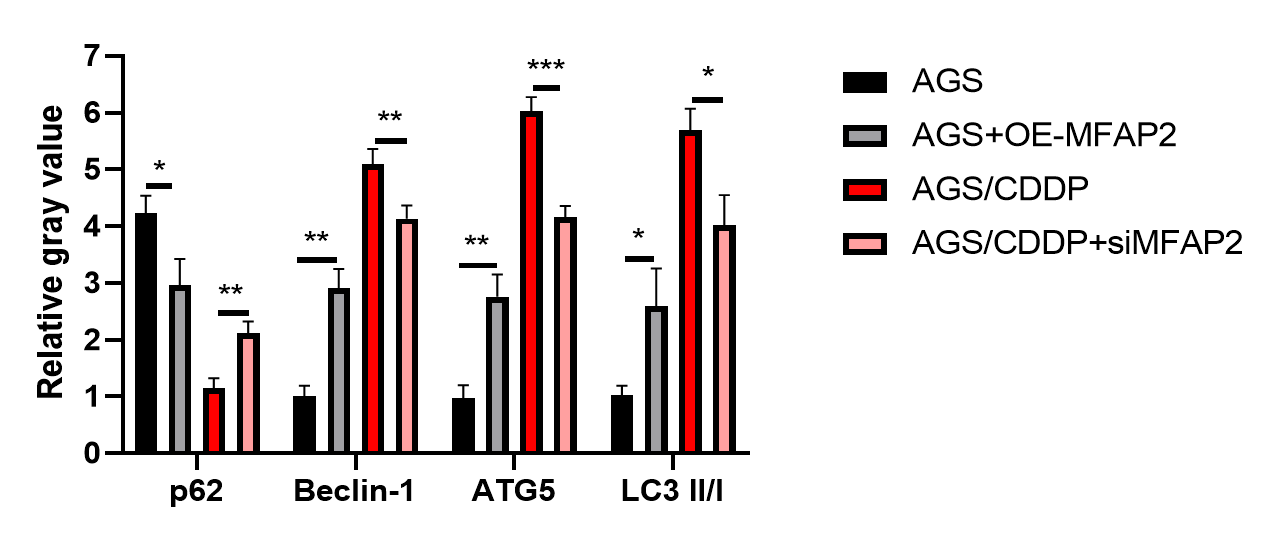

Supplement: Supplemental Information 1 [file peerj-11-15441-s001.zip › Raw data submitted/statistical analysis/Fig. 9.png]

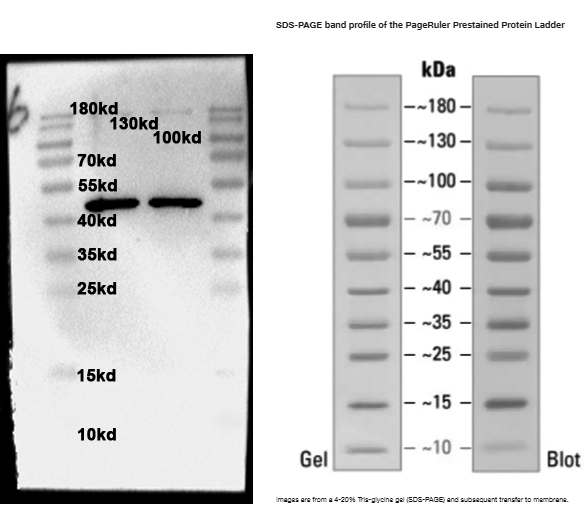

Supplement: Supplemental Information 1 [file peerj-11-15441-s001.zip › Raw data submitted/western blots/(10 to 180 kDa) Thermo Scientific (Cat.26616).tif]

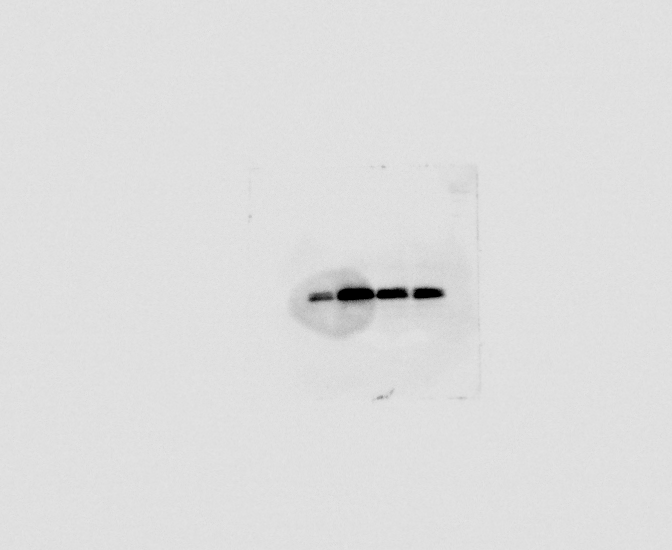

Supplement: Supplemental Information 1 [file peerj-11-15441-s001.zip › Raw data submitted/western blots/Fig. 10A/MFAP2 (1).tif]

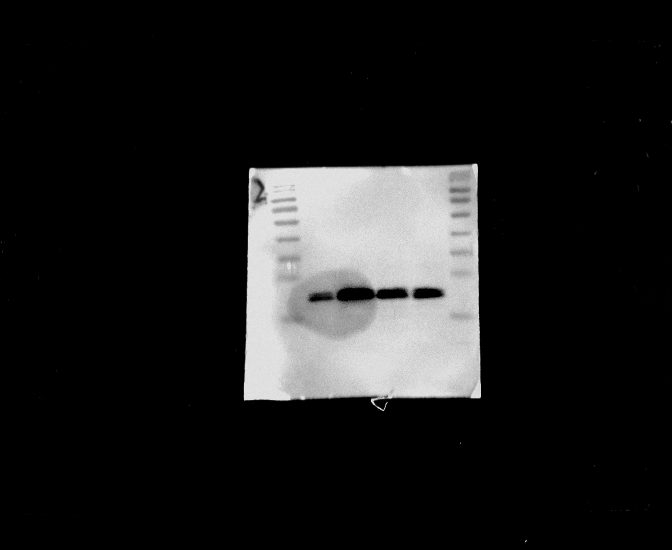

Supplement: Supplemental Information 1 [file peerj-11-15441-s001.zip › Raw data submitted/western blots/Fig. 10A/MFAP2 (2).tif]

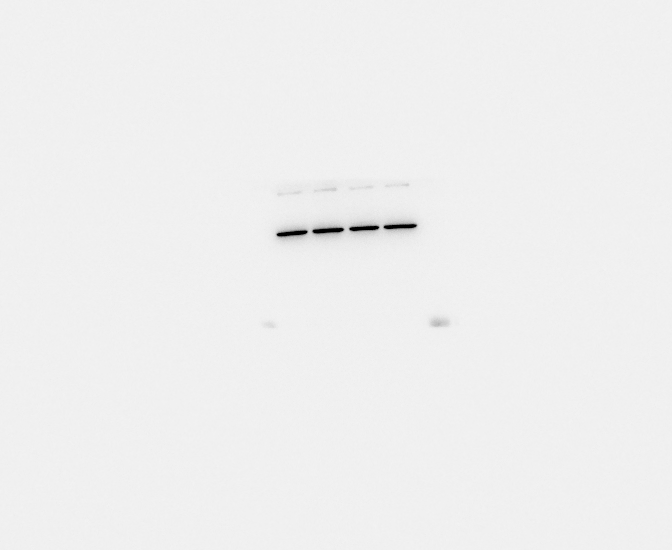

Supplement: Supplemental Information 1 [file peerj-11-15441-s001.zip › Raw data submitted/western blots/Fig. 10A/β-ACTIN (1).tif]

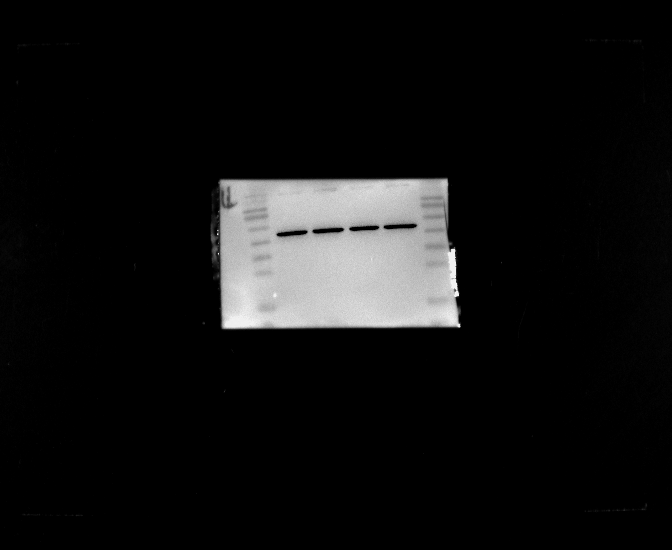

Supplement: Supplemental Information 1 [file peerj-11-15441-s001.zip › Raw data submitted/western blots/Fig. 10A/β-ACTIN (2).tif]

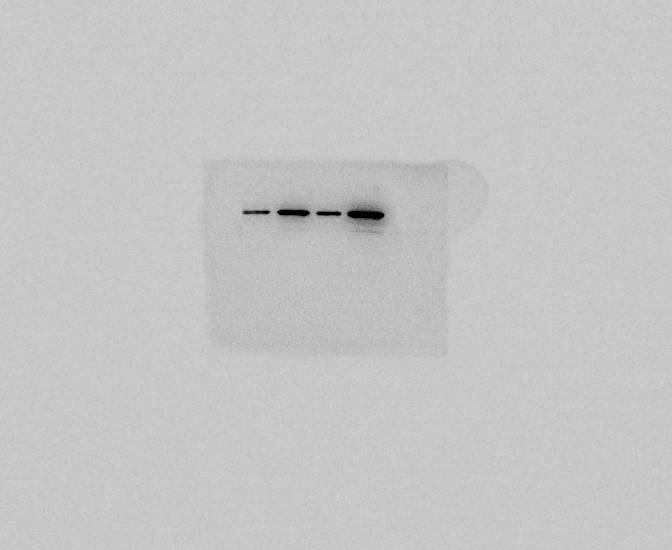

Supplement: Supplemental Information 1 [file peerj-11-15441-s001.zip › Raw data submitted/western blots/Fig. 10B/ATG5 (1).tif]

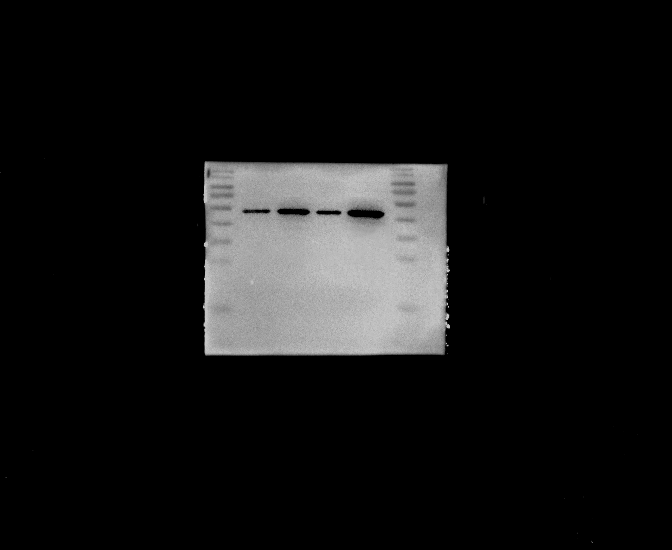

Supplement: Supplemental Information 1 [file peerj-11-15441-s001.zip › Raw data submitted/western blots/Fig. 10B/ATG5 (2).tif]

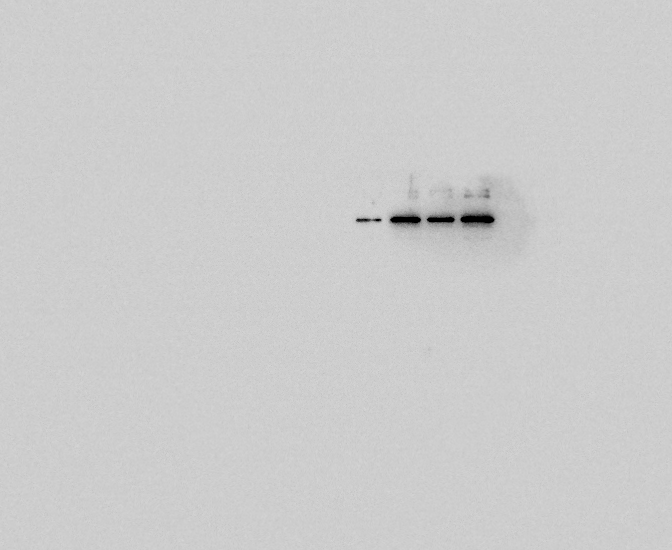

Supplement: Supplemental Information 1 [file peerj-11-15441-s001.zip › Raw data submitted/western blots/Fig. 10B/Beclin-1 (1).tif]

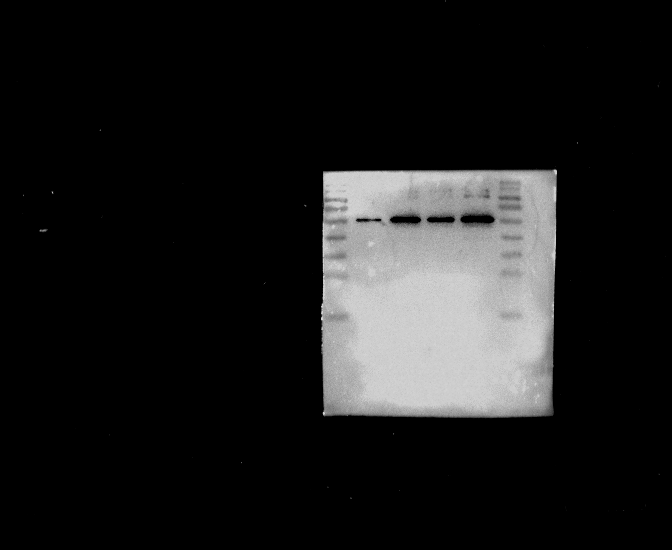

Supplement: Supplemental Information 1 [file peerj-11-15441-s001.zip › Raw data submitted/western blots/Fig. 10B/Beclin-1 (2).tif]

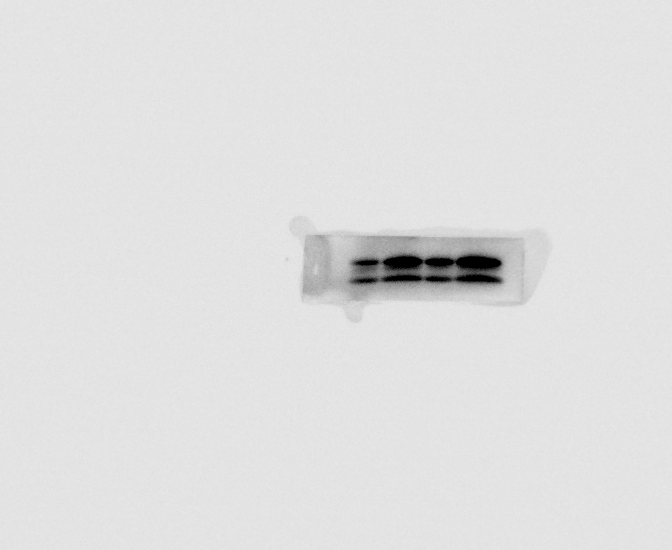

Supplement: Supplemental Information 1 [file peerj-11-15441-s001.zip › Raw data submitted/western blots/Fig. 10B/LC3 (1).tif]

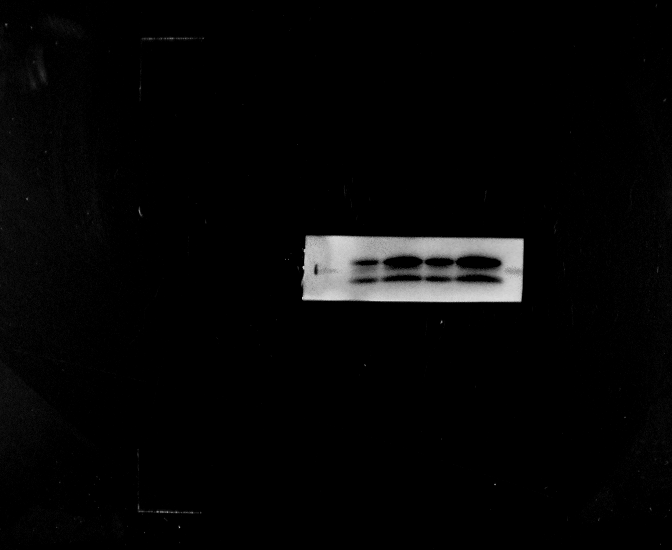

Supplement: Supplemental Information 1 [file peerj-11-15441-s001.zip › Raw data submitted/western blots/Fig. 10B/LC3 (2).tif]

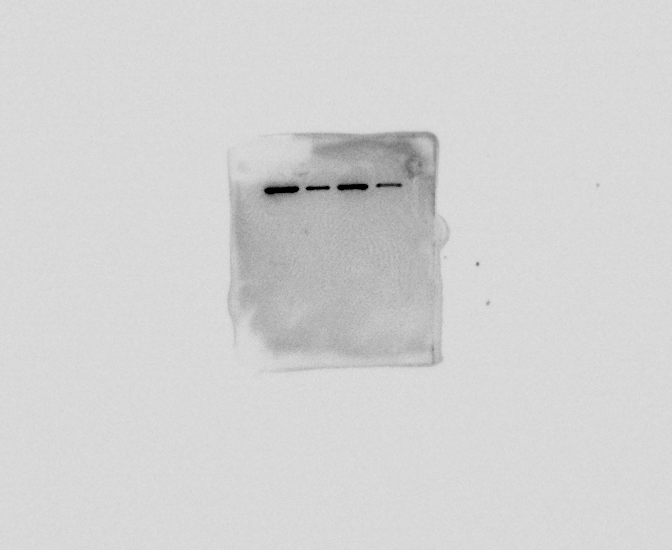

Supplement: Supplemental Information 1 [file peerj-11-15441-s001.zip › Raw data submitted/western blots/Fig. 10B/P62 (1).tif]

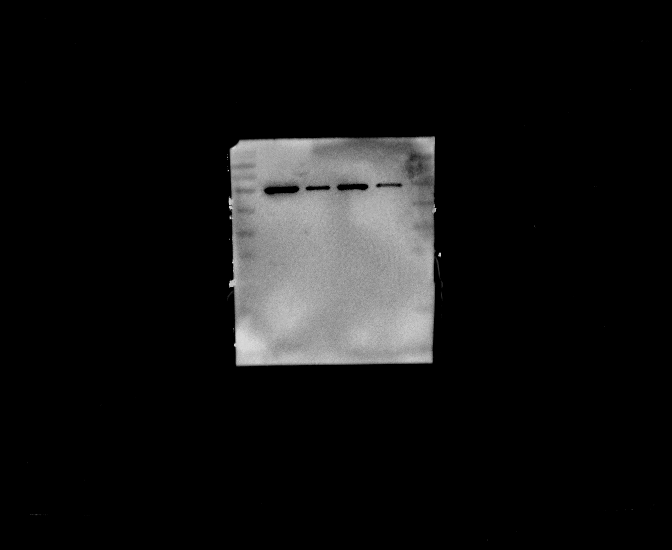

Supplement: Supplemental Information 1 [file peerj-11-15441-s001.zip › Raw data submitted/western blots/Fig. 10B/P62 (2).tif]

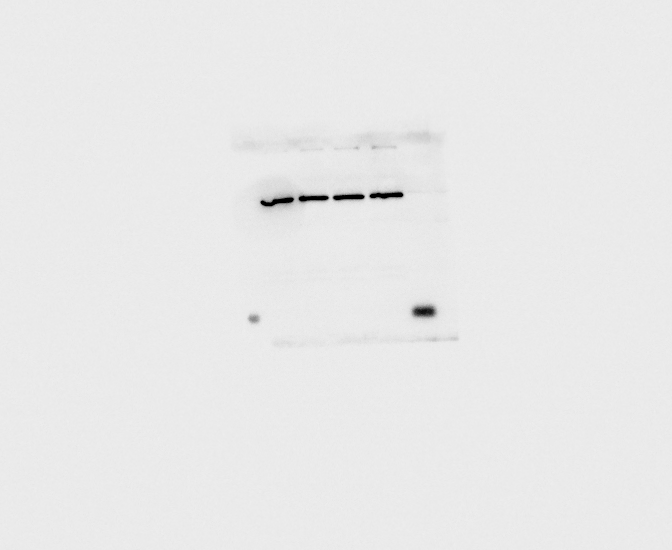

Supplement: Supplemental Information 1 [file peerj-11-15441-s001.zip › Raw data submitted/western blots/Fig. 10B/β-ACTIN (1).tif]

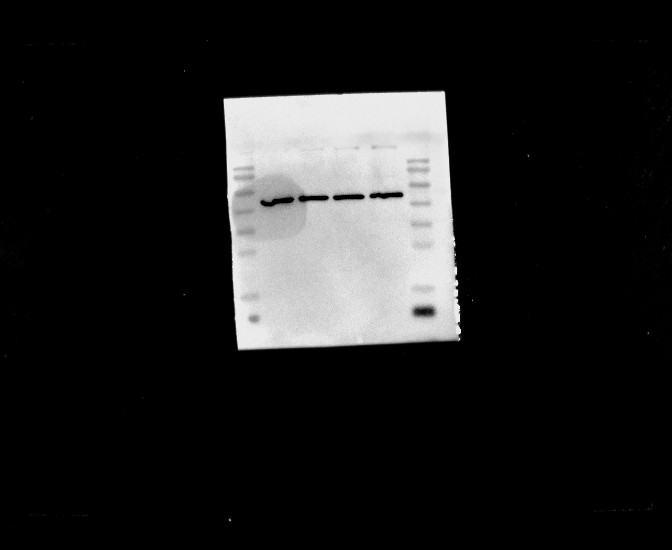

Supplement: Supplemental Information 1 [file peerj-11-15441-s001.zip › Raw data submitted/western blots/Fig. 10B/β-ACTIN (2).tif]

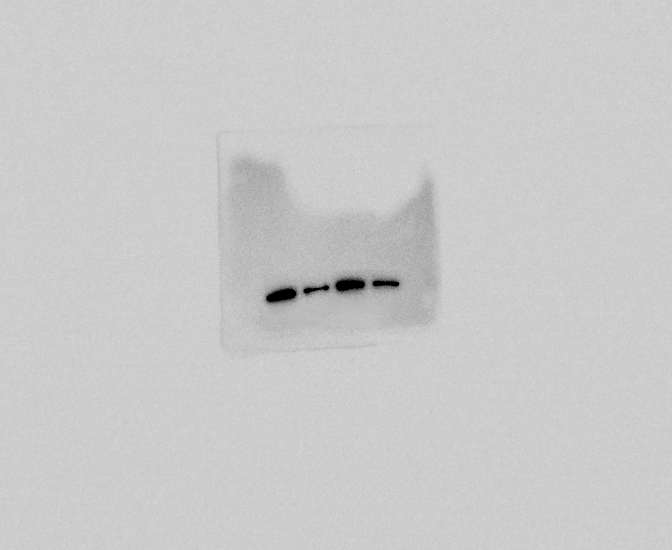

Supplement: Supplemental Information 1 [file peerj-11-15441-s001.zip › Raw data submitted/western blots/Fig. 10E/cleaved caspase 3 (1).tif]

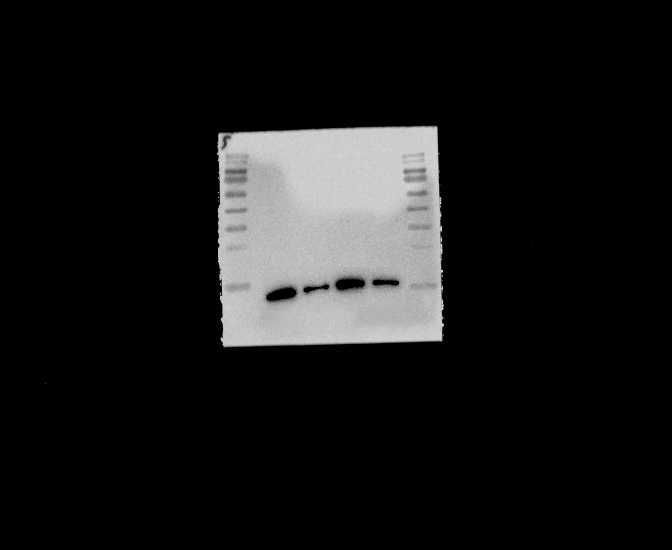

Supplement: Supplemental Information 1 [file peerj-11-15441-s001.zip › Raw data submitted/western blots/Fig. 10E/cleaved caspase 3 (2).tif]

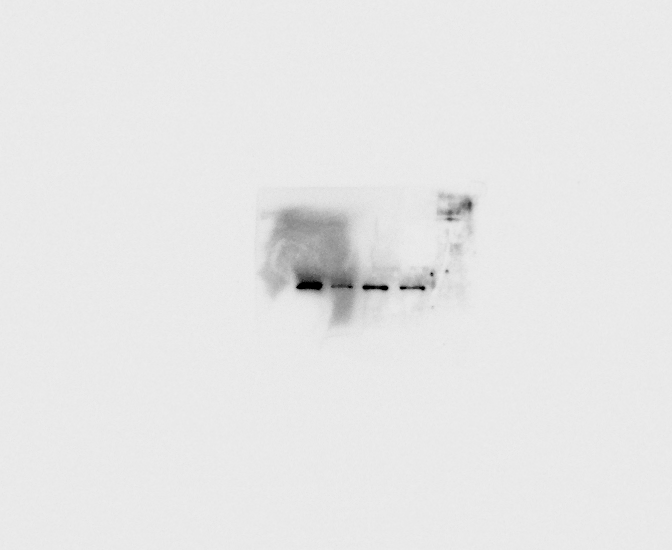

Supplement: Supplemental Information 1 [file peerj-11-15441-s001.zip › Raw data submitted/western blots/Fig. 10E/cleaved caspase 9 (1).tif]

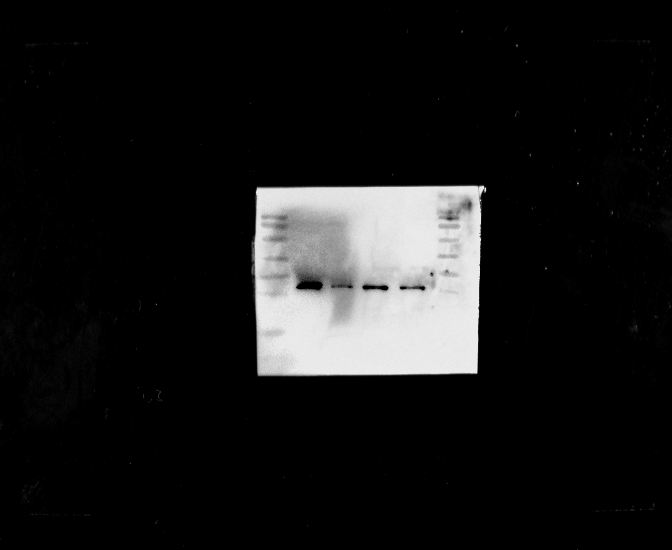

Supplement: Supplemental Information 1 [file peerj-11-15441-s001.zip › Raw data submitted/western blots/Fig. 10E/cleaved caspase 9 (2).tif]

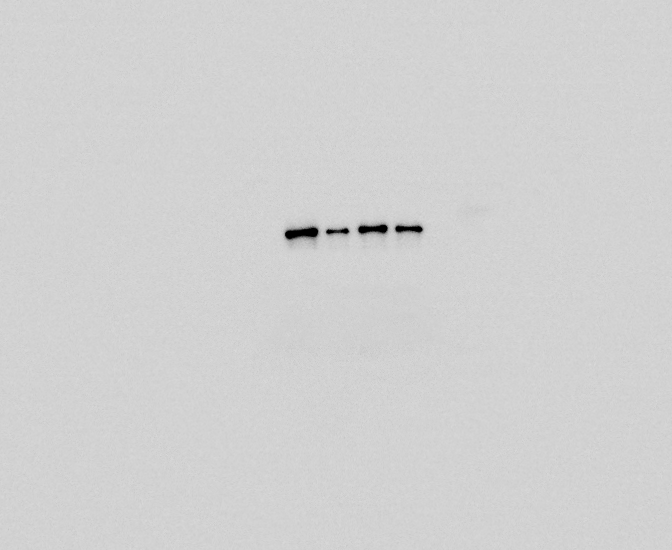

Supplement: Supplemental Information 1 [file peerj-11-15441-s001.zip › Raw data submitted/western blots/Fig. 10E/cleaved PARP (1).tif]

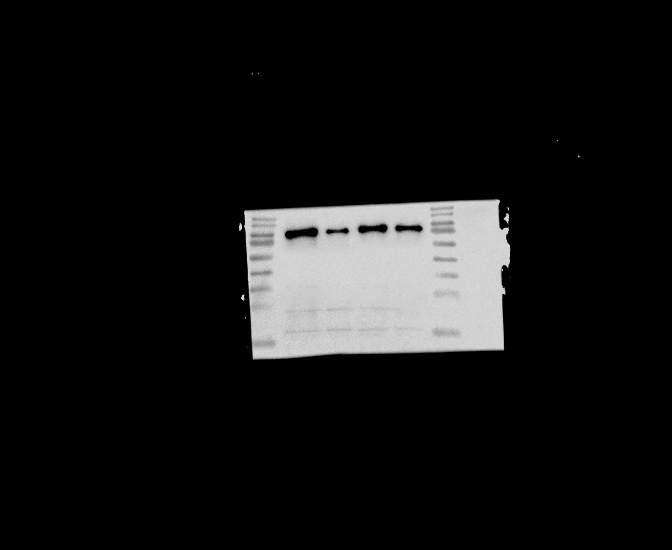

Supplement: Supplemental Information 1 [file peerj-11-15441-s001.zip › Raw data submitted/western blots/Fig. 10E/cleaved PARP (2).tif]

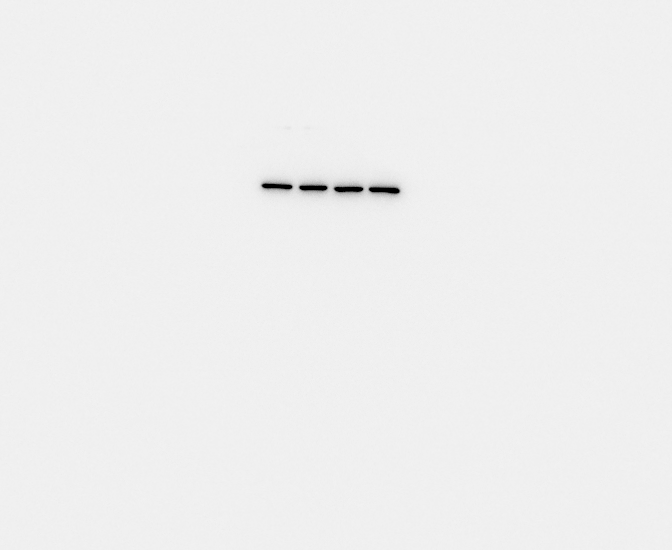

Supplement: Supplemental Information 1 [file peerj-11-15441-s001.zip › Raw data submitted/western blots/Fig. 10E/β-ACTIN (1).tif]

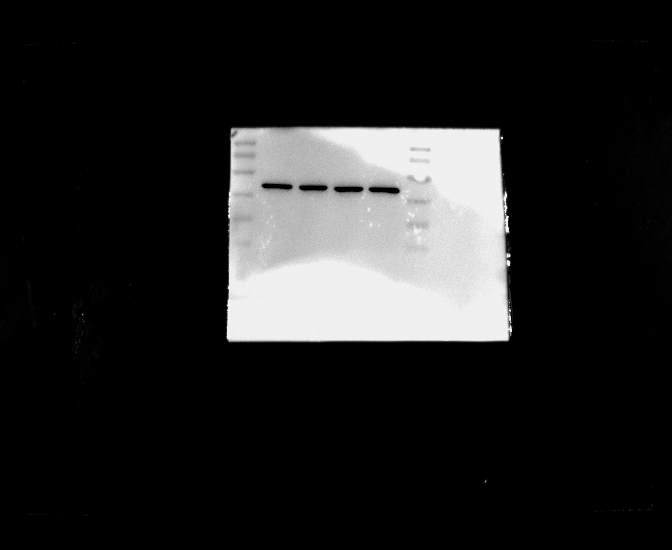

Supplement: Supplemental Information 1 [file peerj-11-15441-s001.zip › Raw data submitted/western blots/Fig. 10E/β-ACTIN (2).tif]

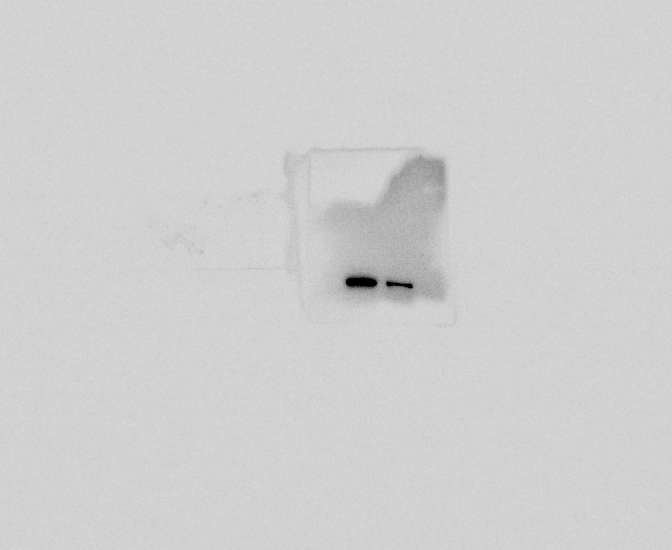

Supplement: Supplemental Information 1 [file peerj-11-15441-s001.zip › Raw data submitted/western blots/Fig. 7C/cleaved caspase 3 (1).tif]

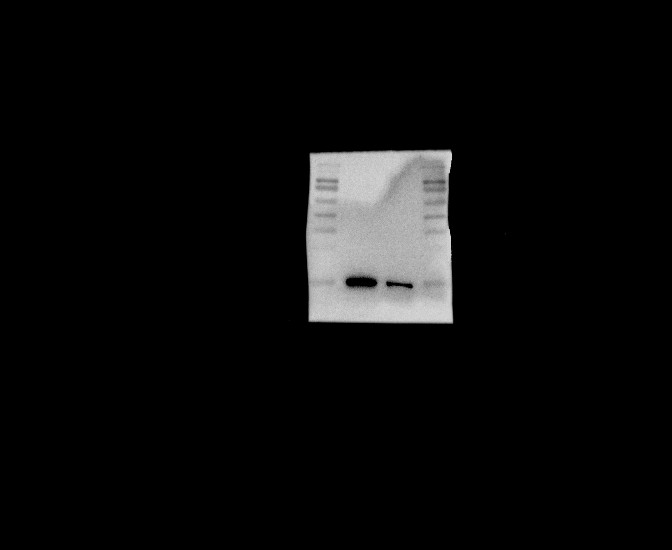

Supplement: Supplemental Information 1 [file peerj-11-15441-s001.zip › Raw data submitted/western blots/Fig. 7C/cleaved caspase 3 (2).tif]

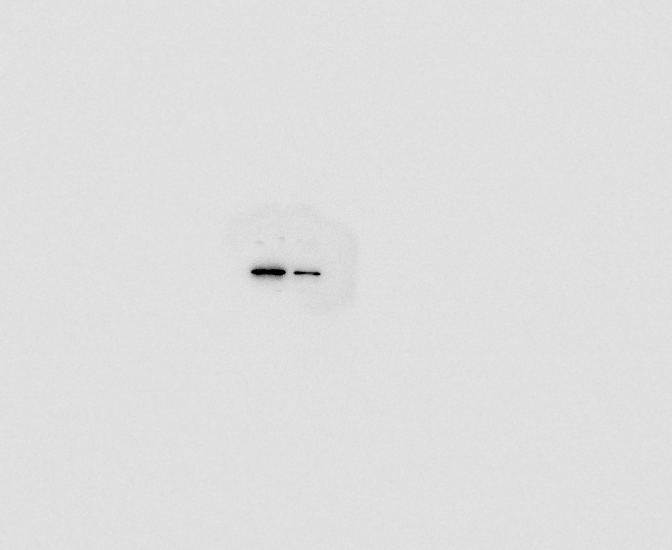

Supplement: Supplemental Information 1 [file peerj-11-15441-s001.zip › Raw data submitted/western blots/Fig. 7C/cleaved caspase 9 (1).tif]

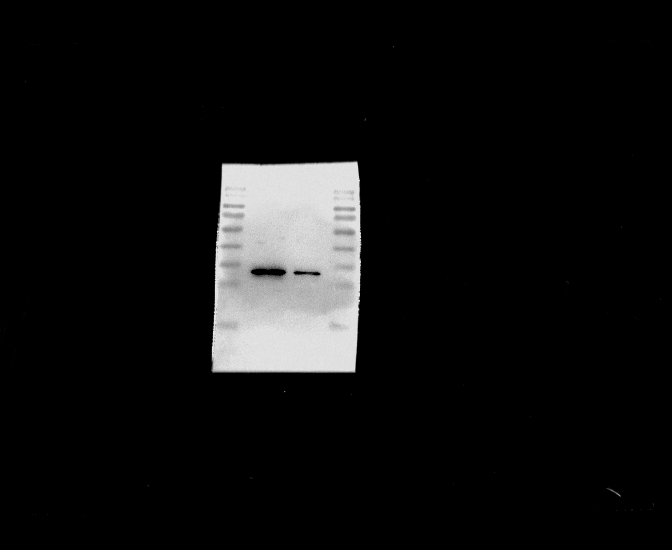

Supplement: Supplemental Information 1 [file peerj-11-15441-s001.zip › Raw data submitted/western blots/Fig. 7C/cleaved caspase 9 (2).tif]

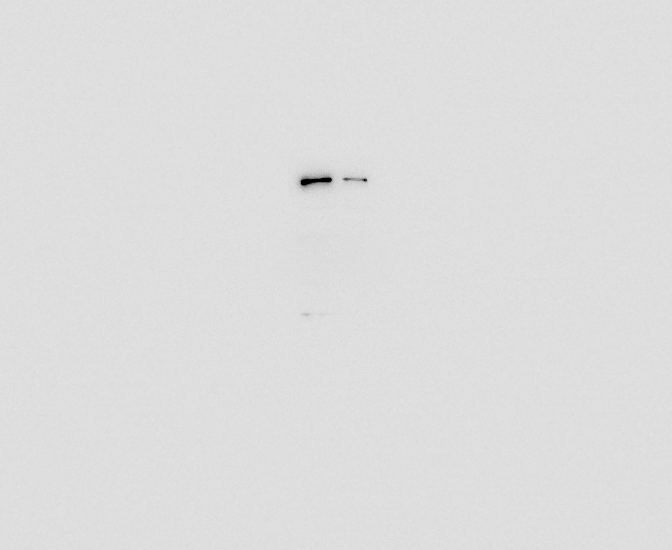

Supplement: Supplemental Information 1 [file peerj-11-15441-s001.zip › Raw data submitted/western blots/Fig. 7C/cleaved PARP (1).tif]

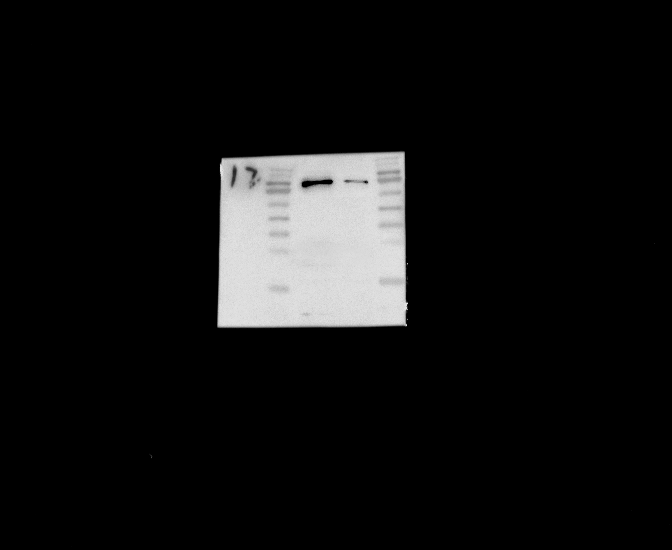

Supplement: Supplemental Information 1 [file peerj-11-15441-s001.zip › Raw data submitted/western blots/Fig. 7C/cleaved PARP (2).tif]

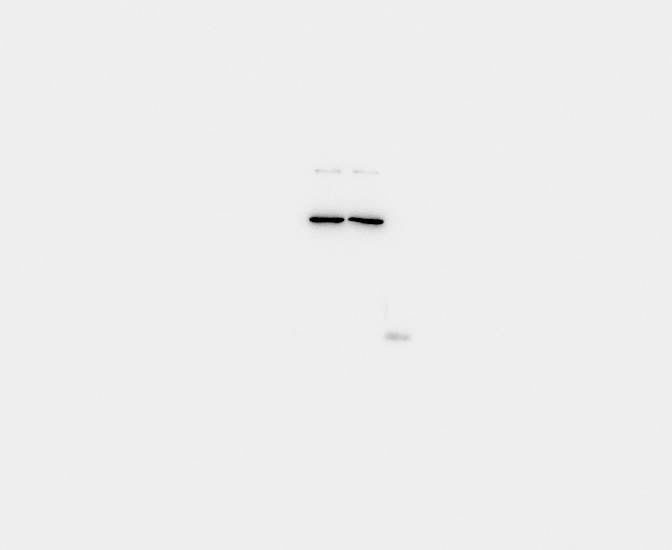

Supplement: Supplemental Information 1 [file peerj-11-15441-s001.zip › Raw data submitted/western blots/Fig. 7C/β-ACTIN (1).tif]

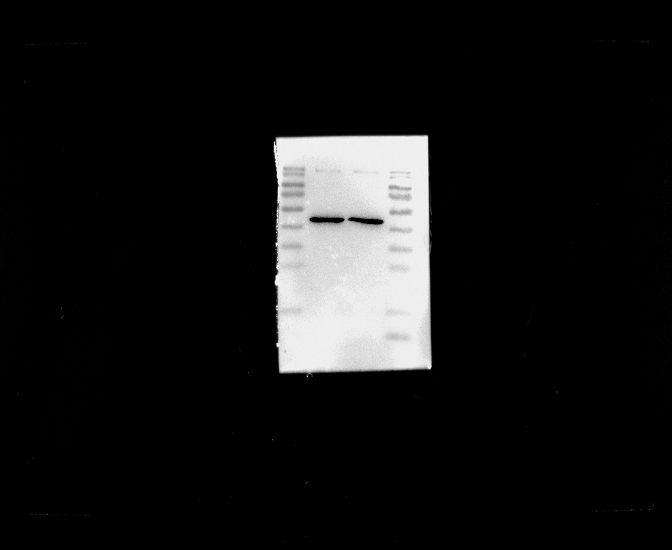

Supplement: Supplemental Information 1 [file peerj-11-15441-s001.zip › Raw data submitted/western blots/Fig. 7C/β-ACTIN (2).tif]

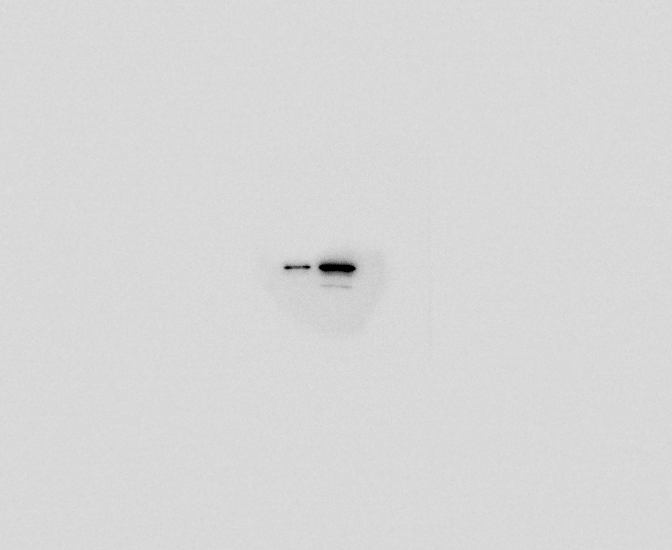

Supplement: Supplemental Information 1 [file peerj-11-15441-s001.zip › Raw data submitted/western blots/Fig. 7D/MFAP2 (1).tif]

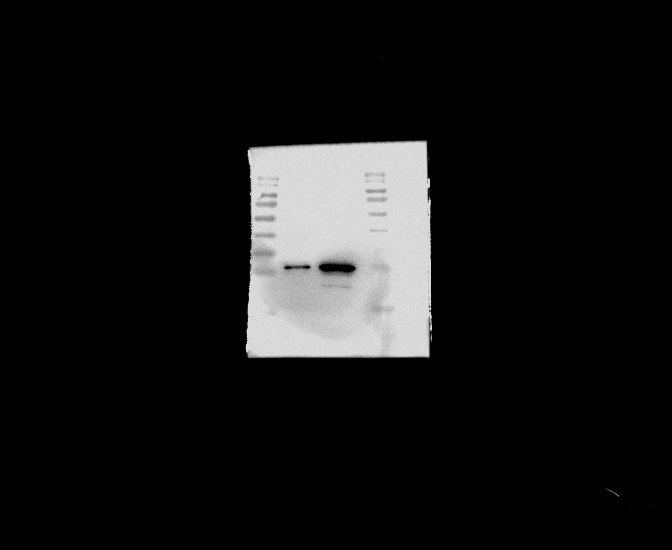

Supplement: Supplemental Information 1 [file peerj-11-15441-s001.zip › Raw data submitted/western blots/Fig. 7D/MFAP2 (2).tif]

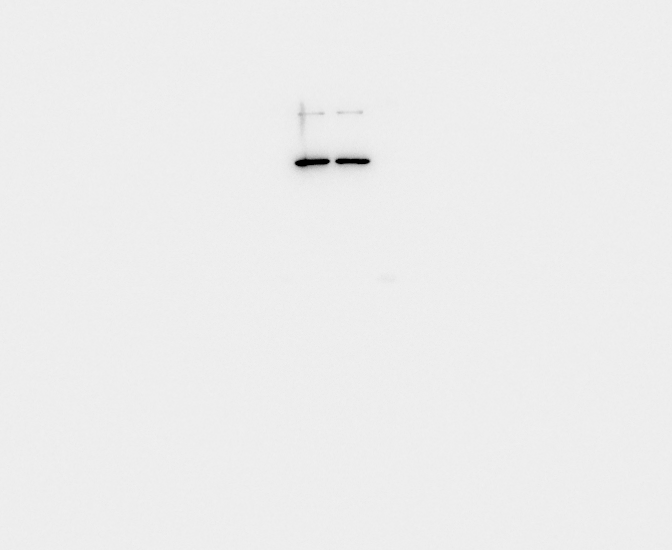

Supplement: Supplemental Information 1 [file peerj-11-15441-s001.zip › Raw data submitted/western blots/Fig. 7D/β-ACTIN (1).tif]

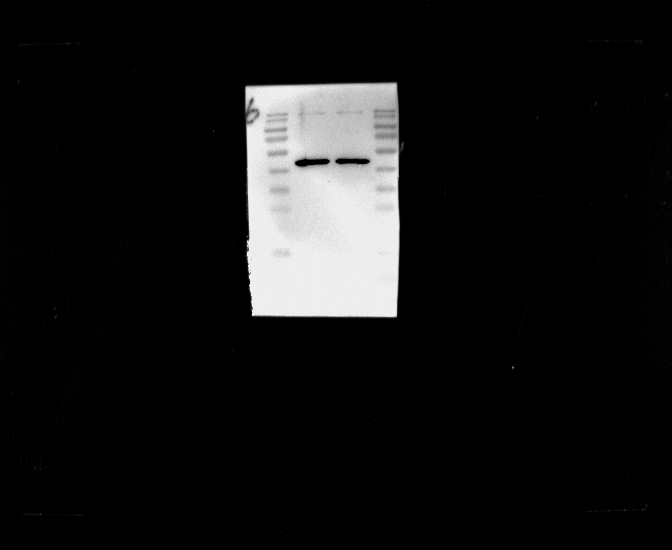

Supplement: Supplemental Information 1 [file peerj-11-15441-s001.zip › Raw data submitted/western blots/Fig. 7D/β-ACTIN (2).tif]

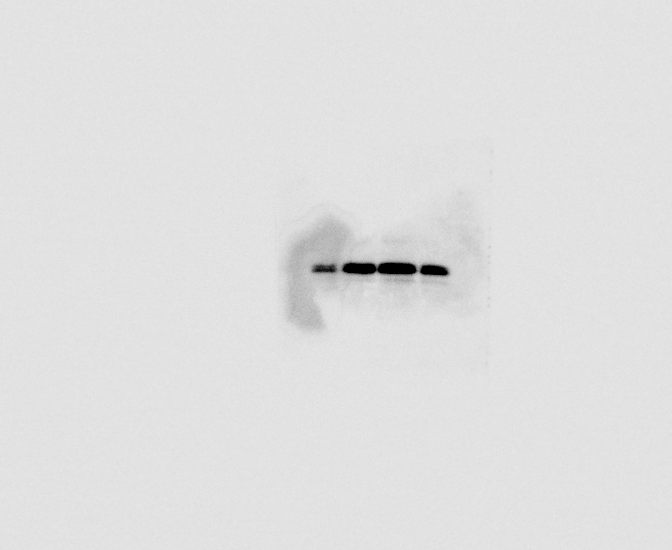

Supplement: Supplemental Information 1 [file peerj-11-15441-s001.zip › Raw data submitted/western blots/Fig. 8A/MFAP2 (1).tif]

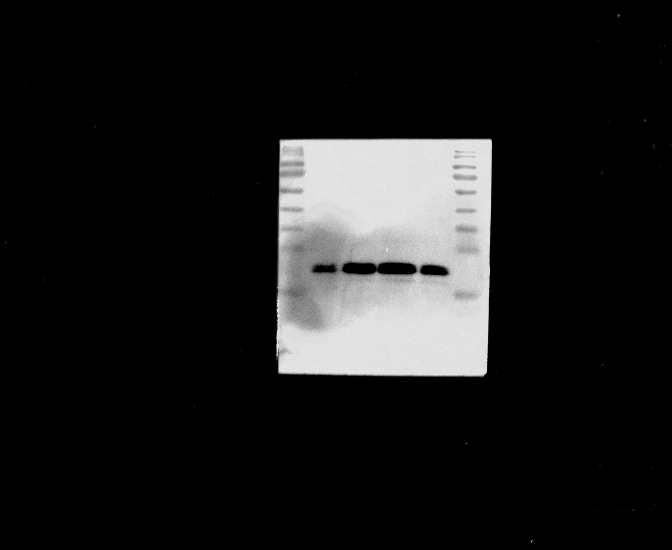

Supplement: Supplemental Information 1 [file peerj-11-15441-s001.zip › Raw data submitted/western blots/Fig. 8A/MFAP2 (2).tif]

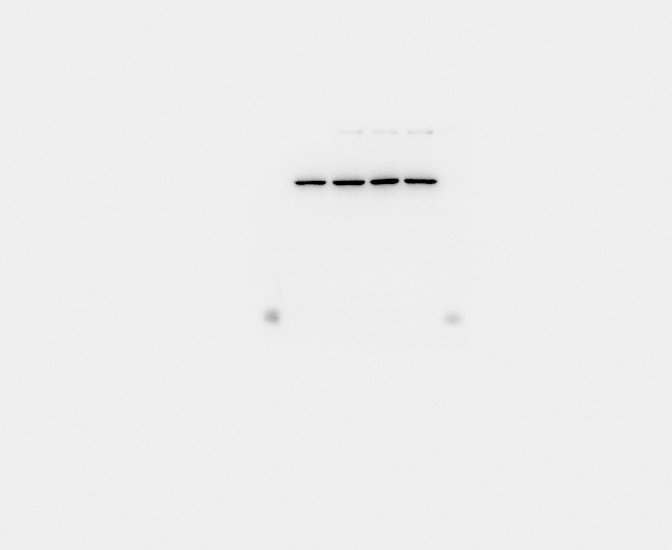

Supplement: Supplemental Information 1 [file peerj-11-15441-s001.zip › Raw data submitted/western blots/Fig. 8A/β-ACTIN (1).tif]

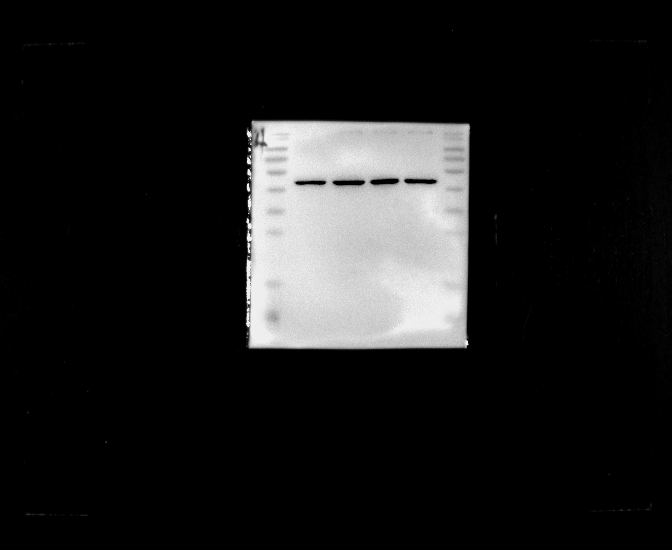

Supplement: Supplemental Information 1 [file peerj-11-15441-s001.zip › Raw data submitted/western blots/Fig. 8A/β-ACTIN (2).tif]

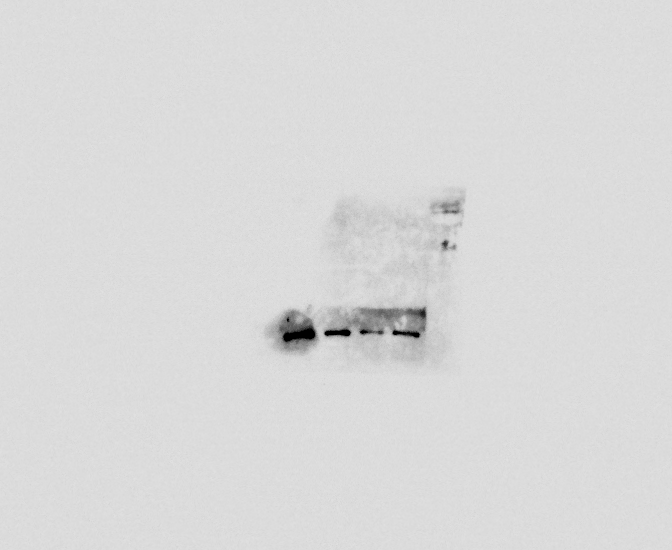

Supplement: Supplemental Information 1 [file peerj-11-15441-s001.zip › Raw data submitted/western blots/Fig. 8D/cleaved caspase 3 (1).tif]

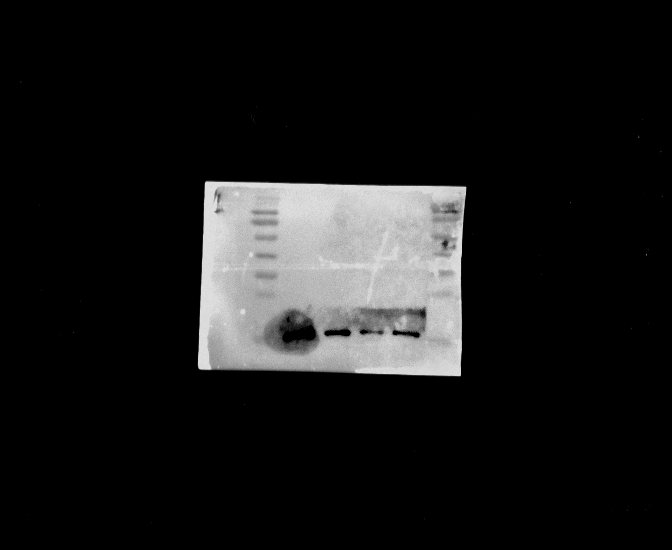

Supplement: Supplemental Information 1 [file peerj-11-15441-s001.zip › Raw data submitted/western blots/Fig. 8D/cleaved caspase 3 (2).tif]

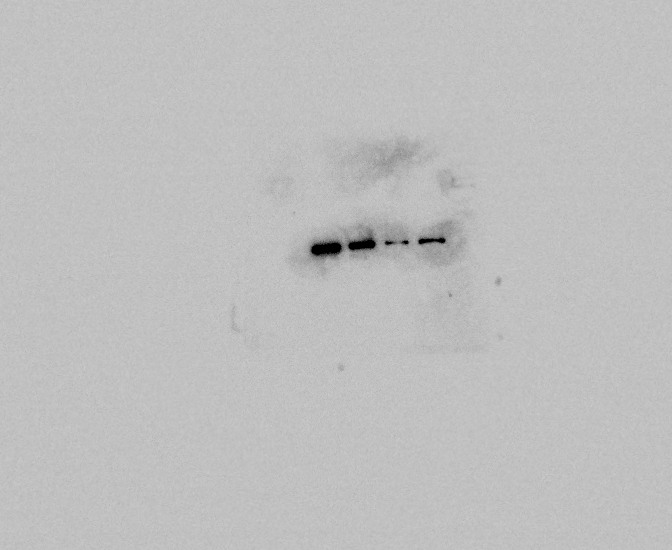

Supplement: Supplemental Information 1 [file peerj-11-15441-s001.zip › Raw data submitted/western blots/Fig. 8D/cleaved caspase 9 (1).tif]

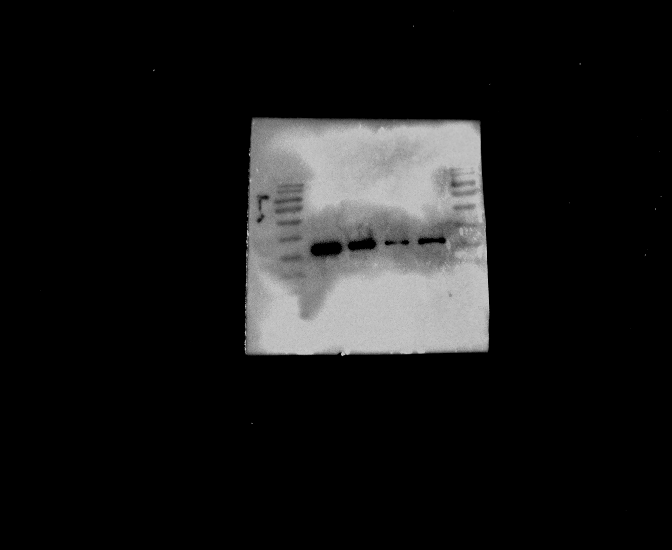

Supplement: Supplemental Information 1 [file peerj-11-15441-s001.zip › Raw data submitted/western blots/Fig. 8D/cleaved caspase 9 (2).tif]

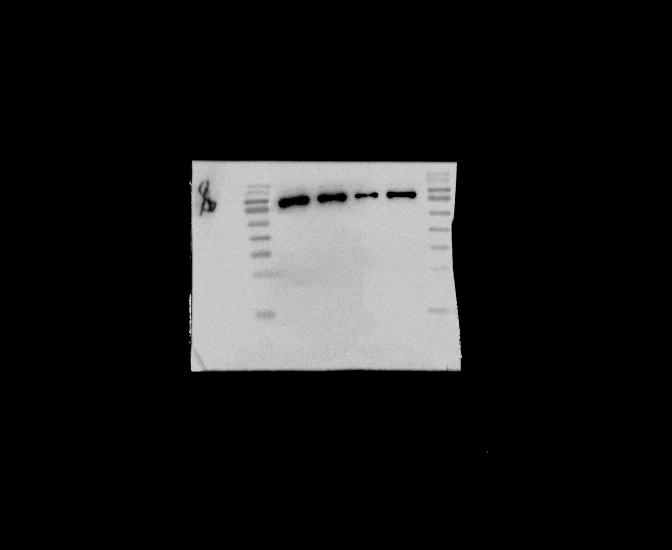

Supplement: Supplemental Information 1 [file peerj-11-15441-s001.zip › Raw data submitted/western blots/Fig. 8D/cleaved PARP (1).tif]

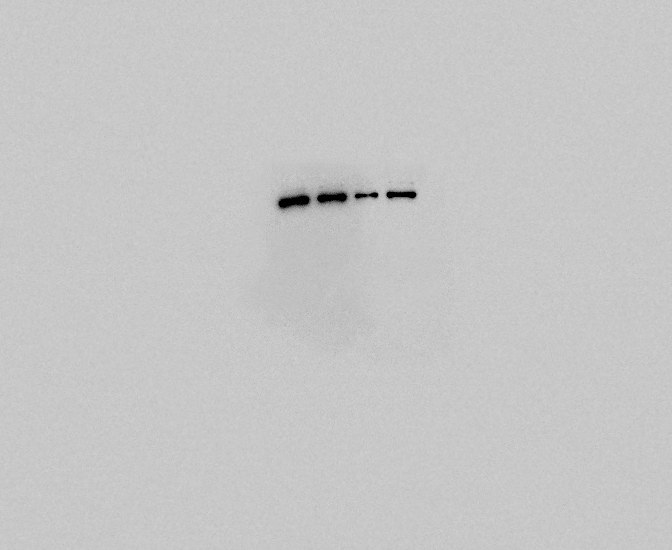

Supplement: Supplemental Information 1 [file peerj-11-15441-s001.zip › Raw data submitted/western blots/Fig. 8D/cleaved PARP (2).tif]

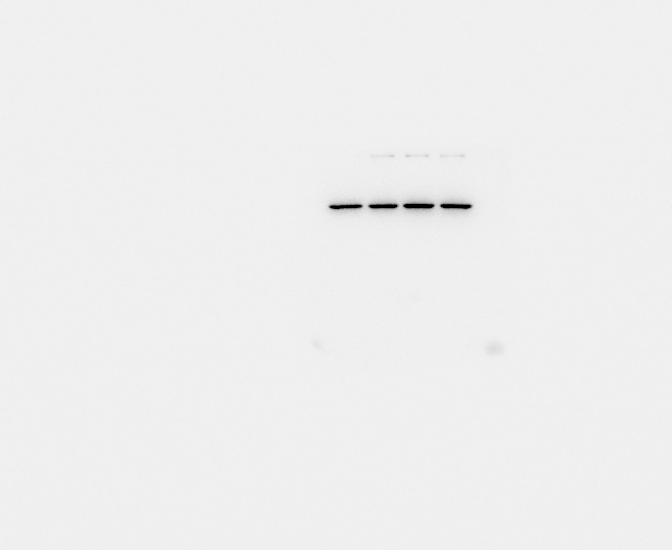

Supplement: Supplemental Information 1 [file peerj-11-15441-s001.zip › Raw data submitted/western blots/Fig. 8D/β-ACTIN (1).tif]

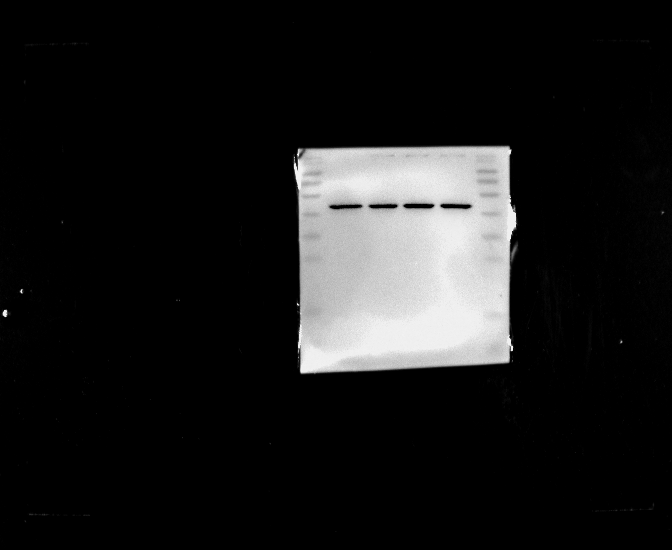

Supplement: Supplemental Information 1 [file peerj-11-15441-s001.zip › Raw data submitted/western blots/Fig. 8D/β-ACTIN (2).tif]

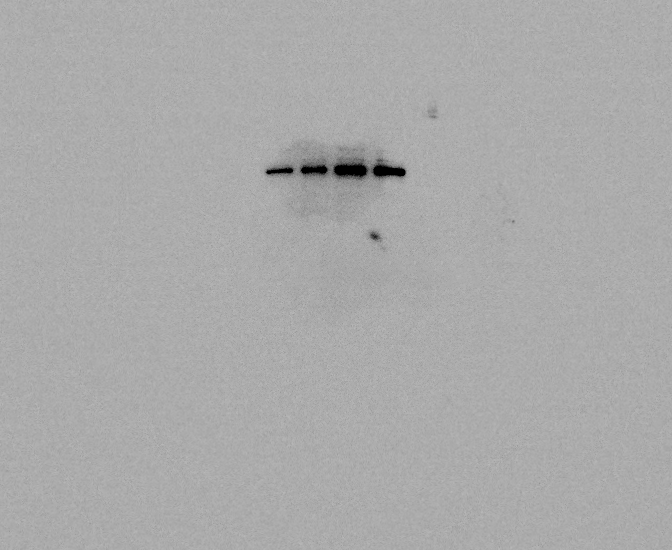

Supplement: Supplemental Information 1 [file peerj-11-15441-s001.zip › Raw data submitted/western blots/Fig. 9/ATG5 (1).tif]

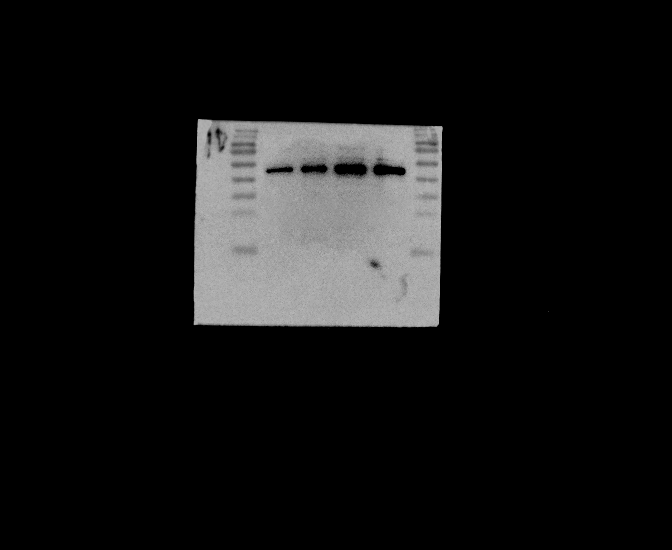

Supplement: Supplemental Information 1 [file peerj-11-15441-s001.zip › Raw data submitted/western blots/Fig. 9/ATG5 (2).tif]

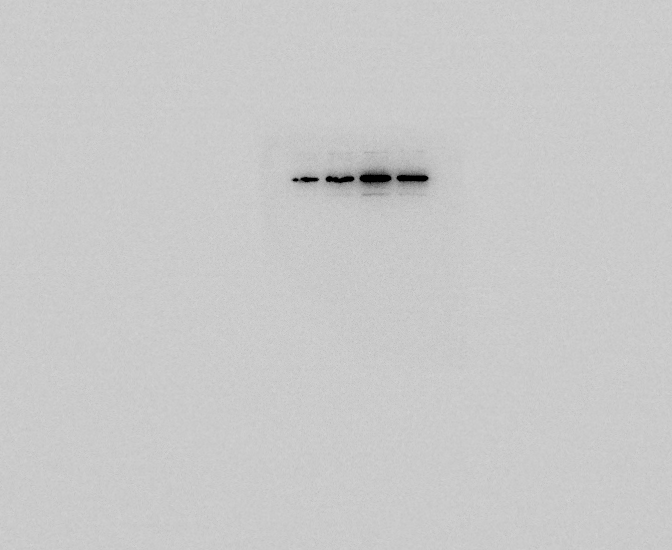

Supplement: Supplemental Information 1 [file peerj-11-15441-s001.zip › Raw data submitted/western blots/Fig. 9/Beclin-1 (1).tif]

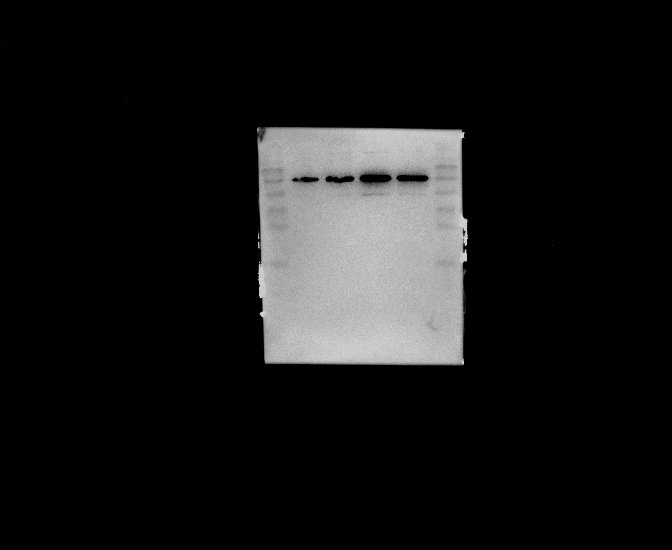

Supplement: Supplemental Information 1 [file peerj-11-15441-s001.zip › Raw data submitted/western blots/Fig. 9/Beclin-1 (2).tif]

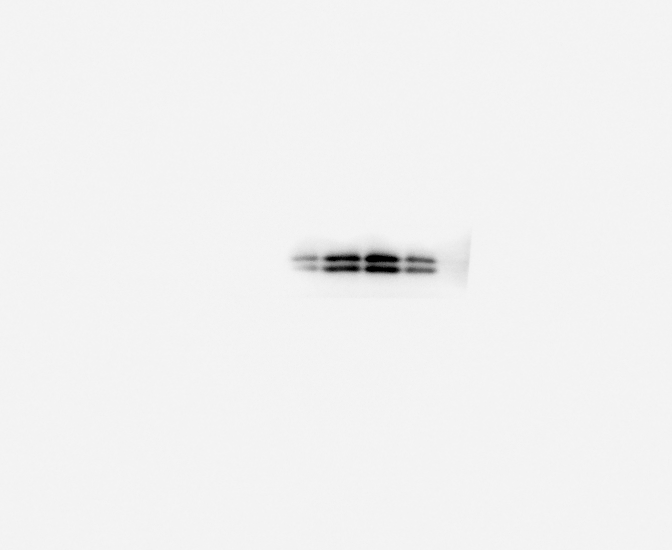

Supplement: Supplemental Information 1 [file peerj-11-15441-s001.zip › Raw data submitted/western blots/Fig. 9/LC3 (1).tif]

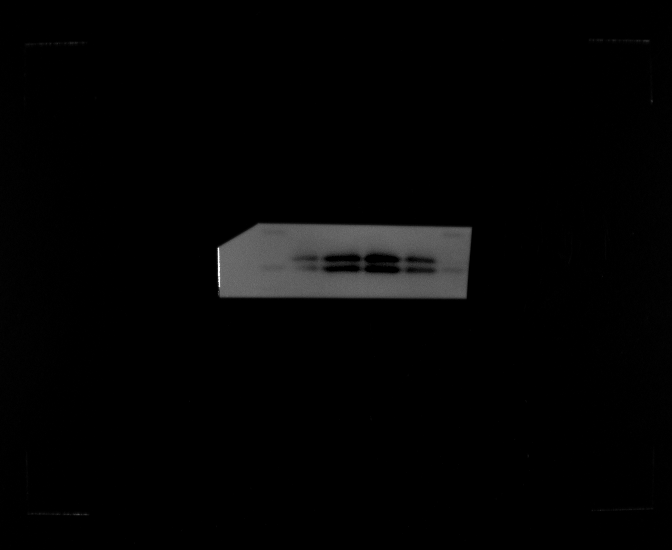

Supplement: Supplemental Information 1 [file peerj-11-15441-s001.zip › Raw data submitted/western blots/Fig. 9/LC3 (2).tif]

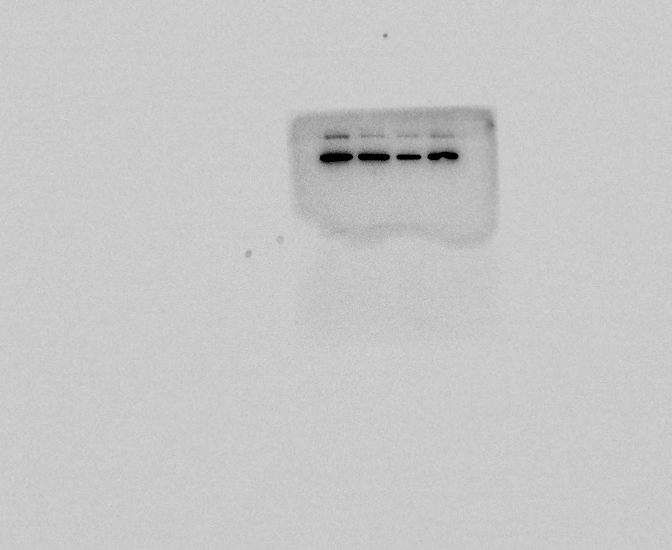

Supplement: Supplemental Information 1 [file peerj-11-15441-s001.zip › Raw data submitted/western blots/Fig. 9/P62 (1).tif]

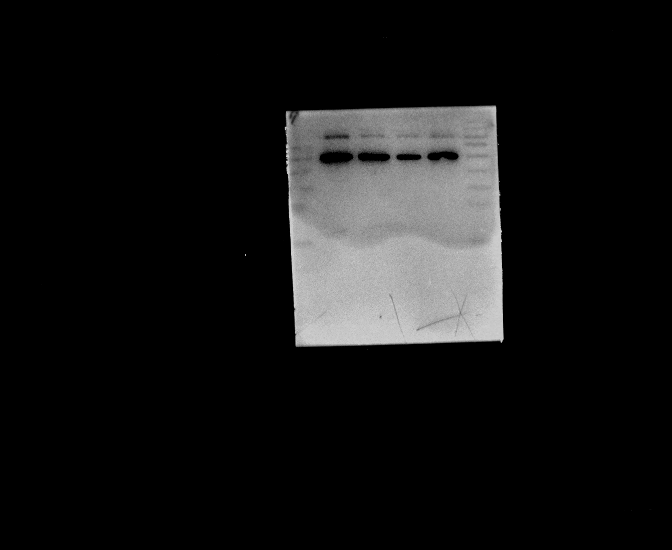

Supplement: Supplemental Information 1 [file peerj-11-15441-s001.zip › Raw data submitted/western blots/Fig. 9/P62 (2).tif]
