# Supplementary figures and images for: MFAP2 enhances cisplatin resistance in gastric cancer cells by regulating autophagy (part 1 of 2)
Source: PeerJ. 2023 Jun 7;11:e15441. doi: 10.7717/peerj.15441 (PMC10257393; doi:10.7717/peerj.15441)

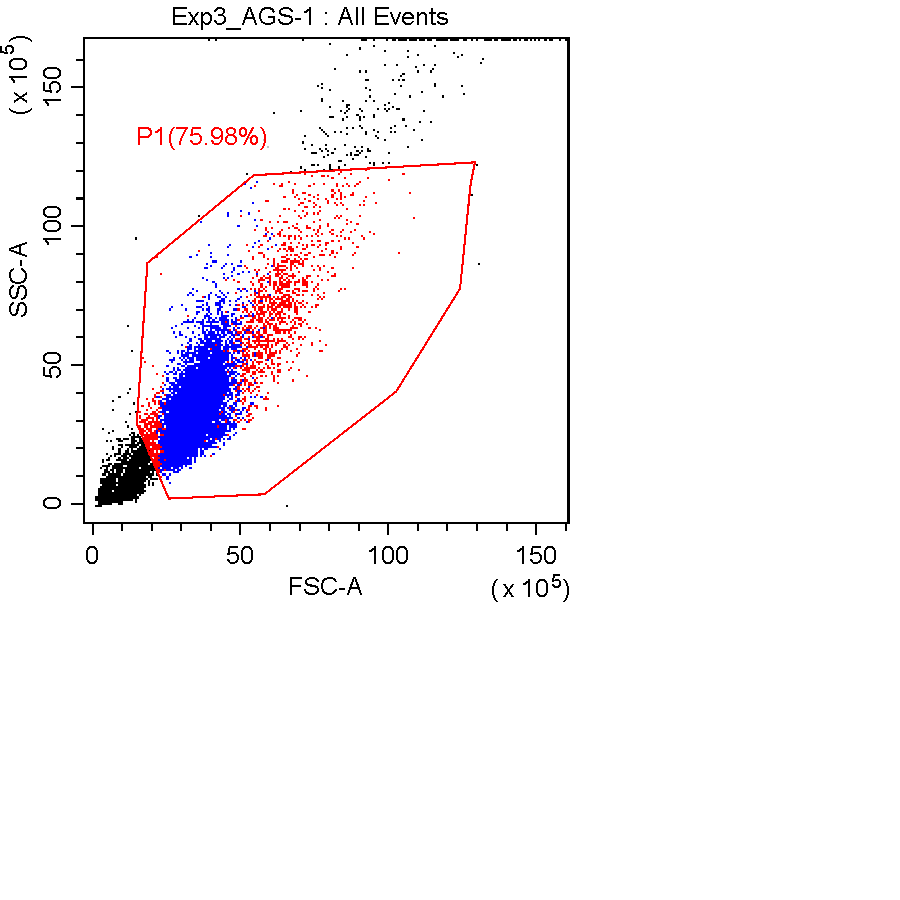

Supplement: Supplemental Information 1 [file peerj-11-15441-s001.zip › Raw data submitted/cell apoptosis/Fig. 10/AGS/Exp3_AGS-1_Plot1.bmp]

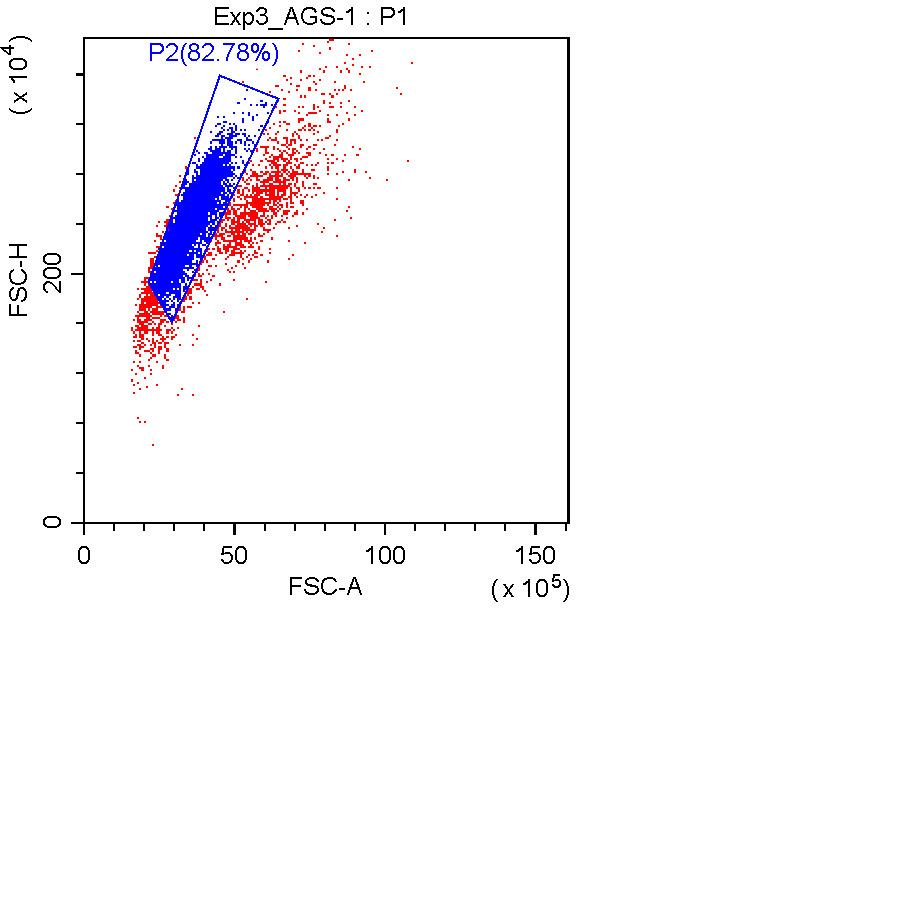

Supplement: Supplemental Information 1 [file peerj-11-15441-s001.zip › Raw data submitted/cell apoptosis/Fig. 10/AGS/Exp3_AGS-1_Plot2.bmp]

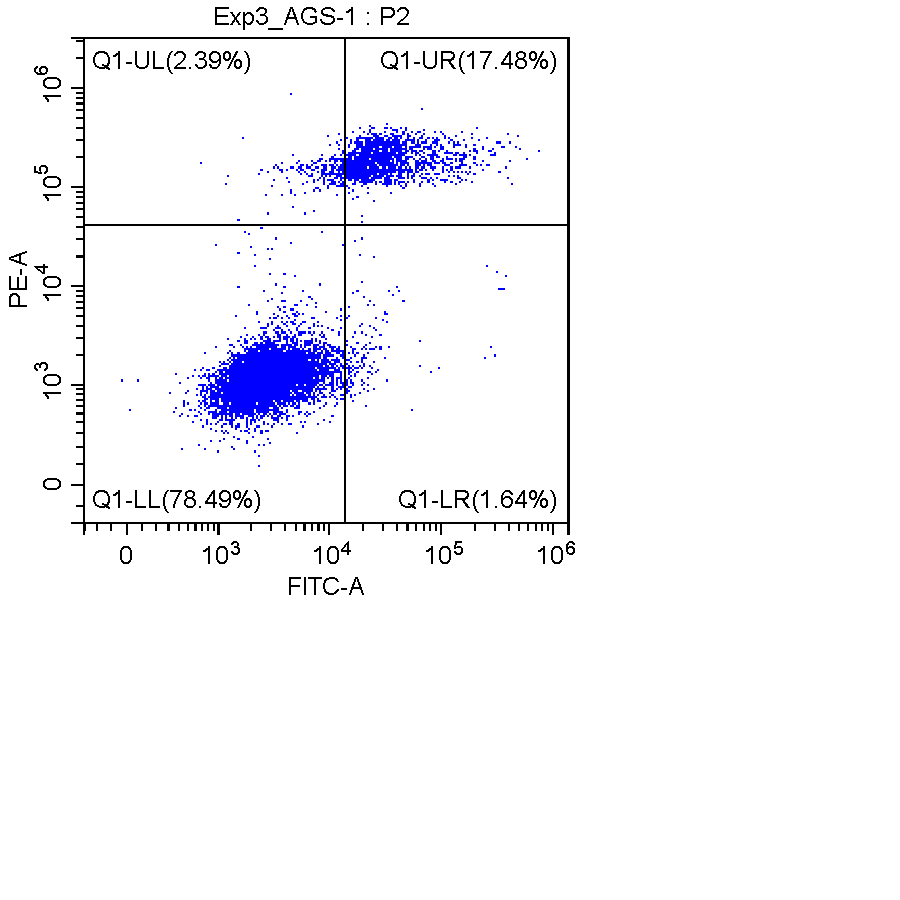

Supplement: Supplemental Information 1 [file peerj-11-15441-s001.zip › Raw data submitted/cell apoptosis/Fig. 10/AGS/Exp3_AGS-1_Plot3.bmp]

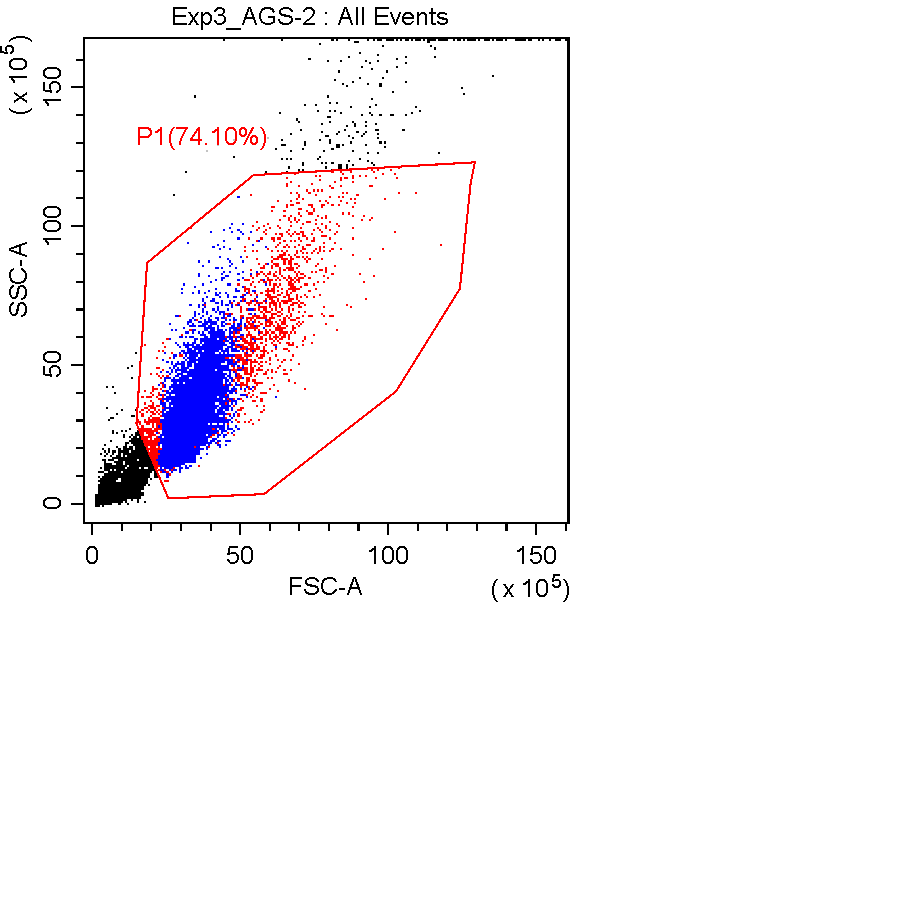

Supplement: Supplemental Information 1 [file peerj-11-15441-s001.zip › Raw data submitted/cell apoptosis/Fig. 10/AGS/Exp3_AGS-2_Plot1.bmp]

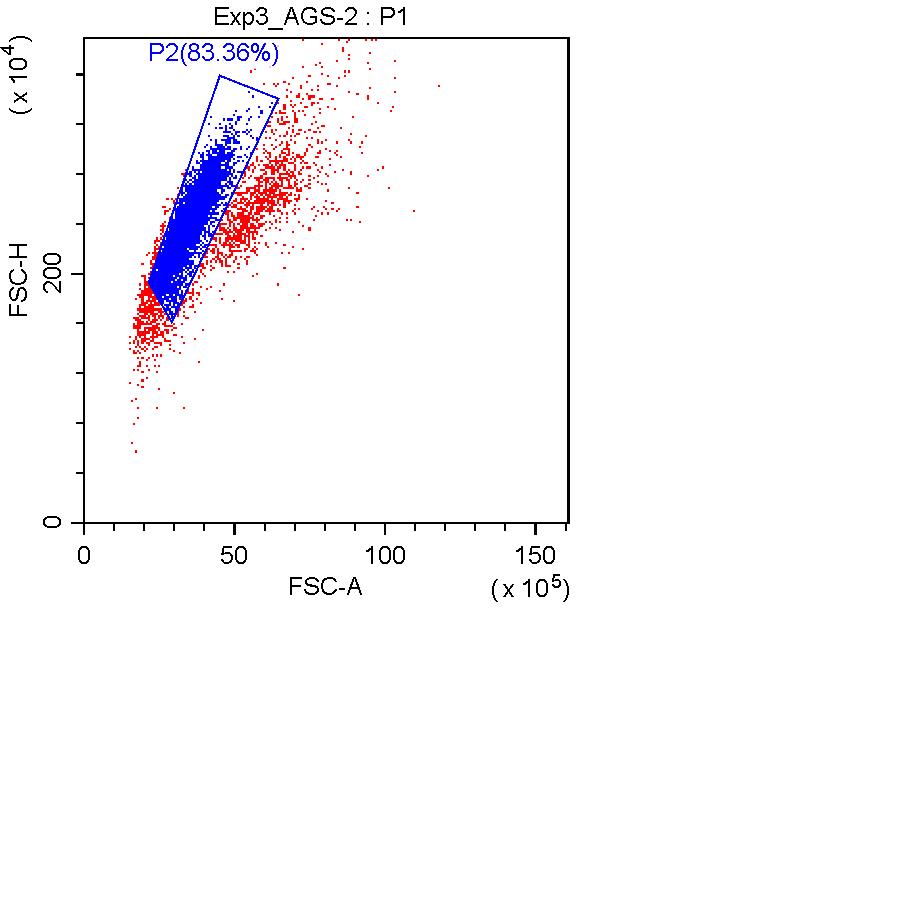

Supplement: Supplemental Information 1 [file peerj-11-15441-s001.zip › Raw data submitted/cell apoptosis/Fig. 10/AGS/Exp3_AGS-2_Plot2.bmp]

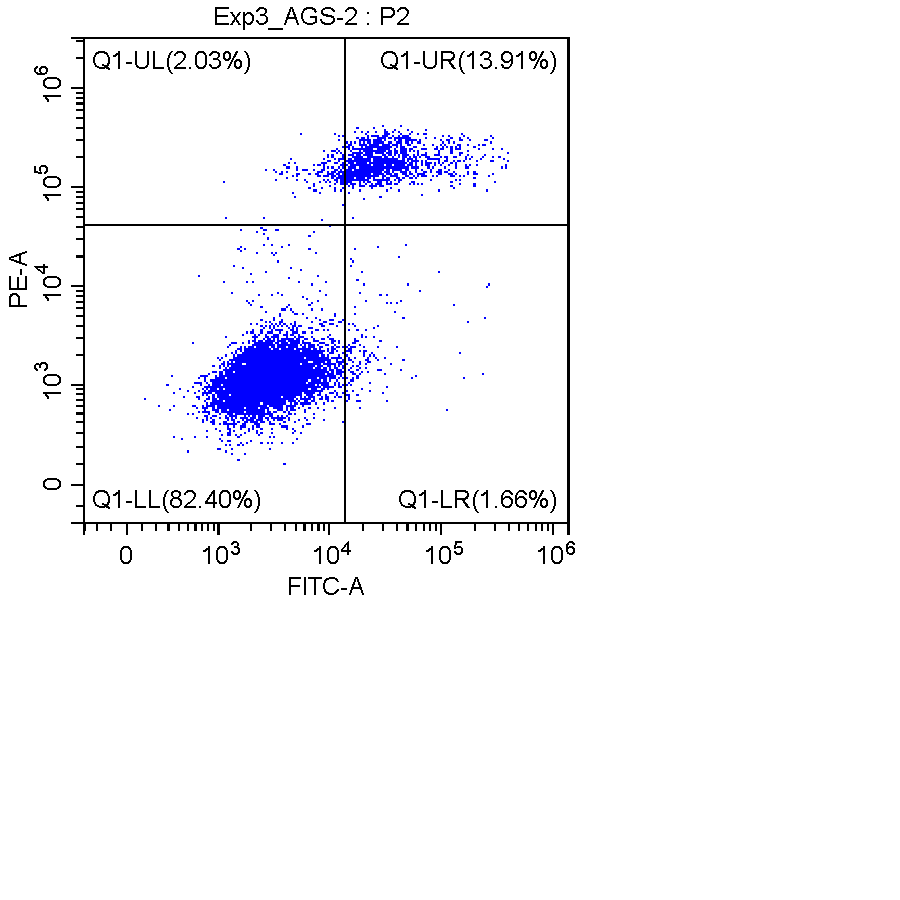

Supplement: Supplemental Information 1 [file peerj-11-15441-s001.zip › Raw data submitted/cell apoptosis/Fig. 10/AGS/Exp3_AGS-2_Plot3.bmp]

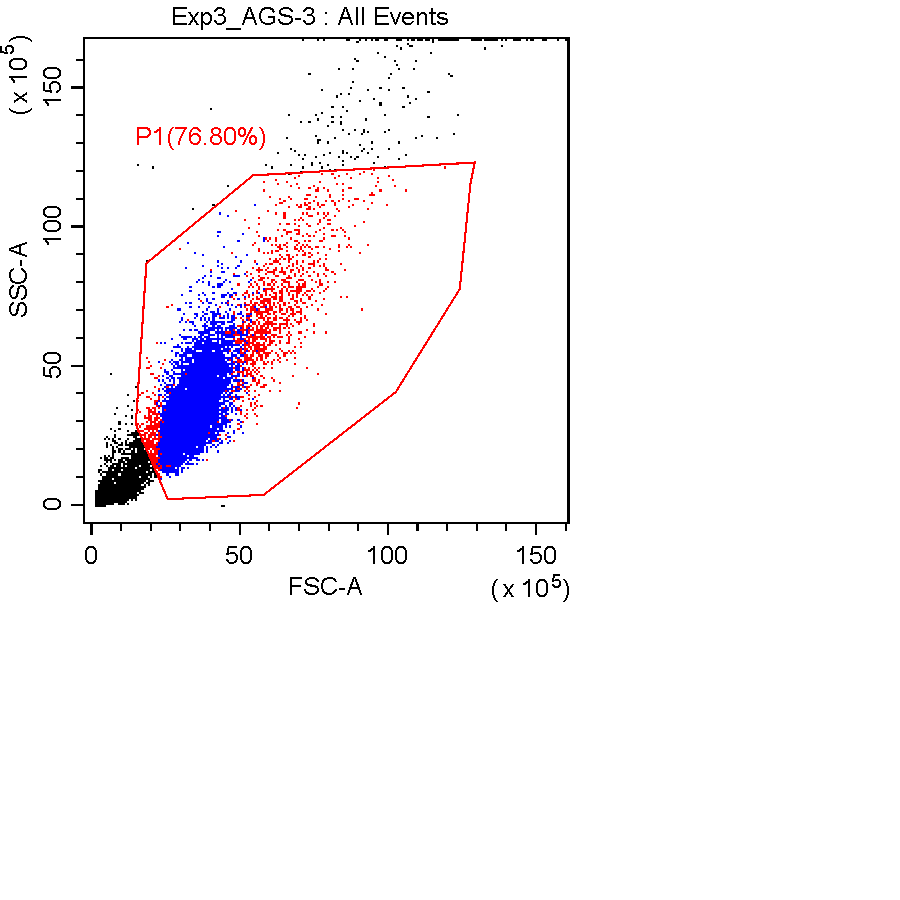

Supplement: Supplemental Information 1 [file peerj-11-15441-s001.zip › Raw data submitted/cell apoptosis/Fig. 10/AGS/Exp3_AGS-3_Plot1.bmp]

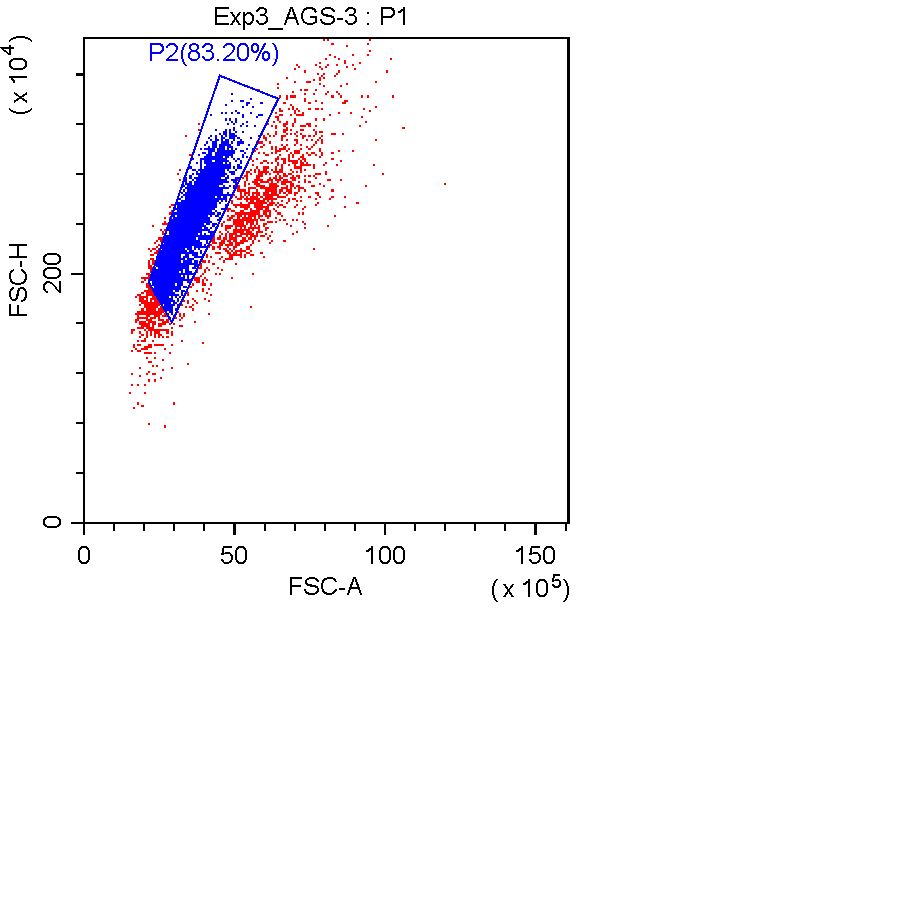

Supplement: Supplemental Information 1 [file peerj-11-15441-s001.zip › Raw data submitted/cell apoptosis/Fig. 10/AGS/Exp3_AGS-3_Plot2.bmp]

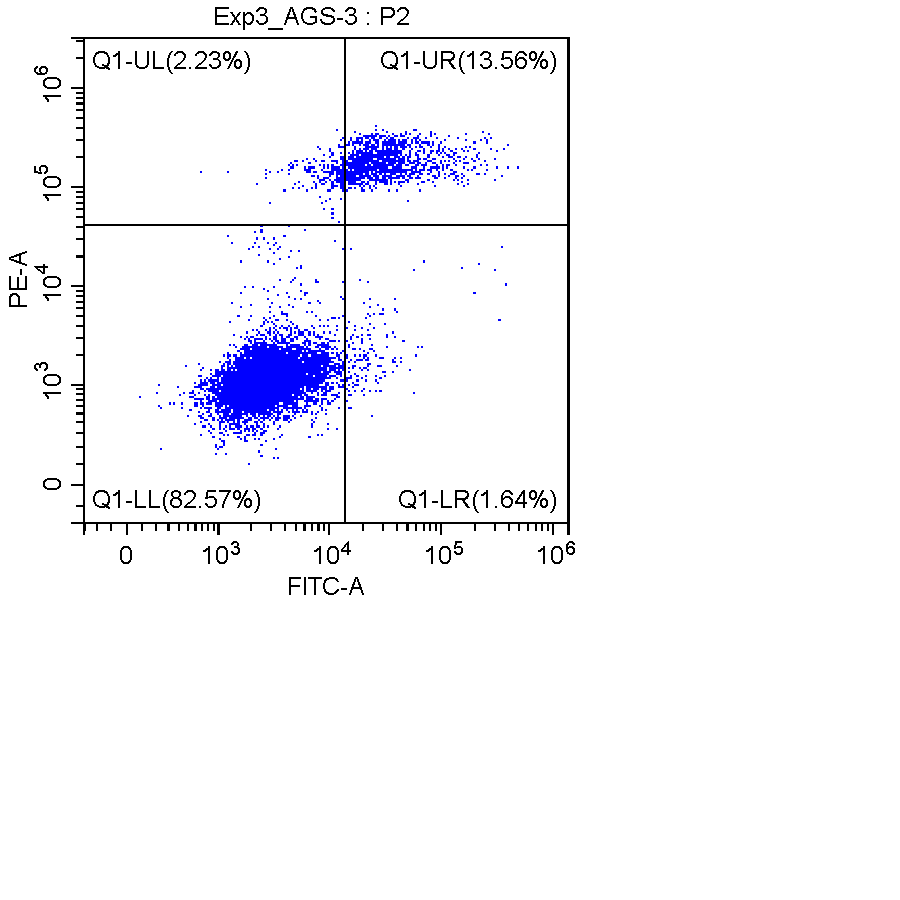

Supplement: Supplemental Information 1 [file peerj-11-15441-s001.zip › Raw data submitted/cell apoptosis/Fig. 10/AGS/Exp3_AGS-3_Plot3.bmp]

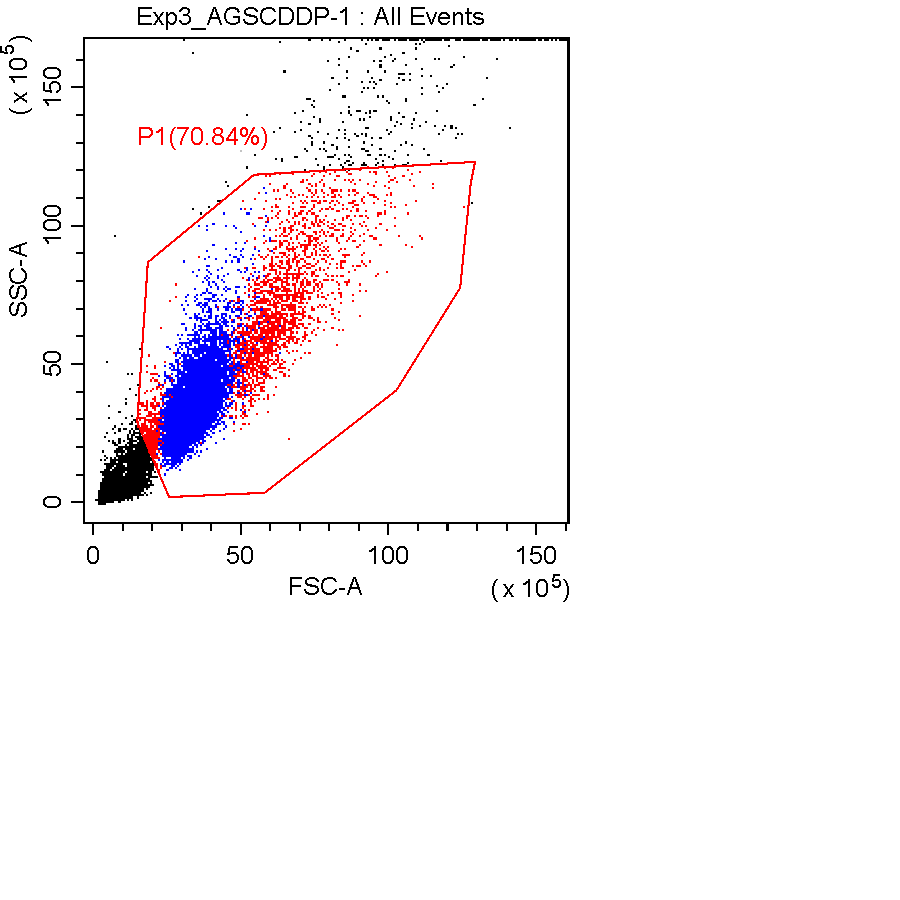

Supplement: Supplemental Information 1 [file peerj-11-15441-s001.zip › Raw data submitted/cell apoptosis/Fig. 10/AGSCDDP/Exp3_AGSCDDP-1_Plot1.bmp]

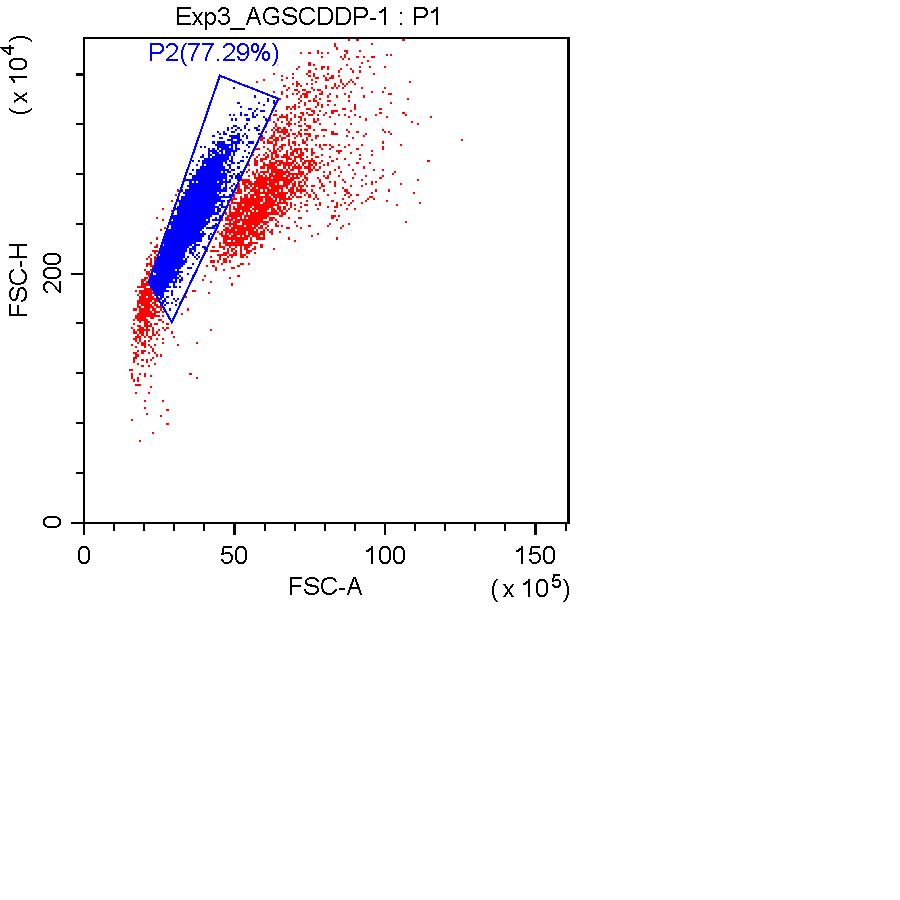

Supplement: Supplemental Information 1 [file peerj-11-15441-s001.zip › Raw data submitted/cell apoptosis/Fig. 10/AGSCDDP/Exp3_AGSCDDP-1_Plot2.bmp]

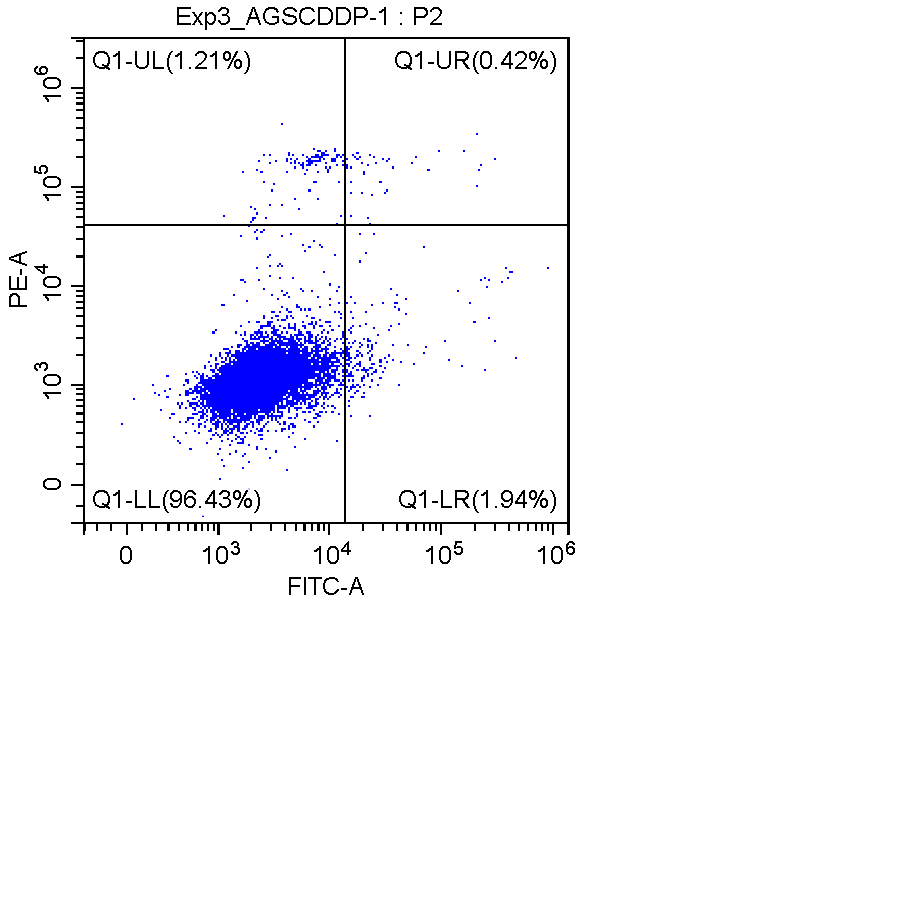

Supplement: Supplemental Information 1 [file peerj-11-15441-s001.zip › Raw data submitted/cell apoptosis/Fig. 10/AGSCDDP/Exp3_AGSCDDP-1_Plot3.bmp]

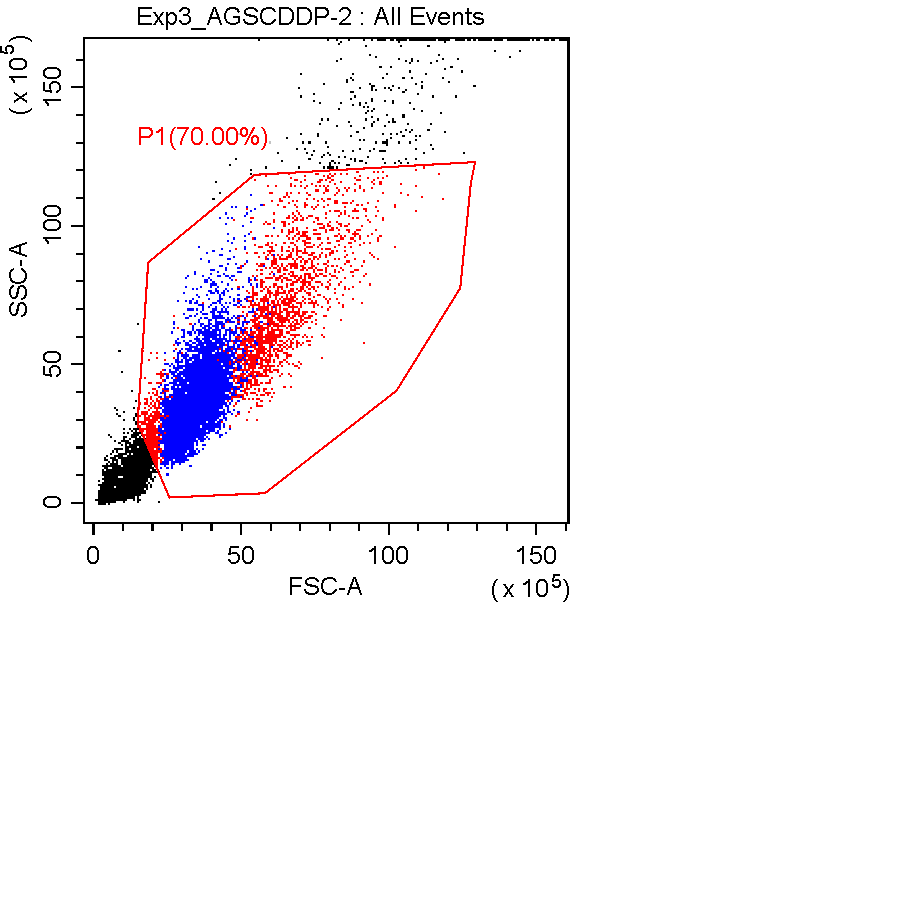

Supplement: Supplemental Information 1 [file peerj-11-15441-s001.zip › Raw data submitted/cell apoptosis/Fig. 10/AGSCDDP/Exp3_AGSCDDP-2_Plot1.bmp]

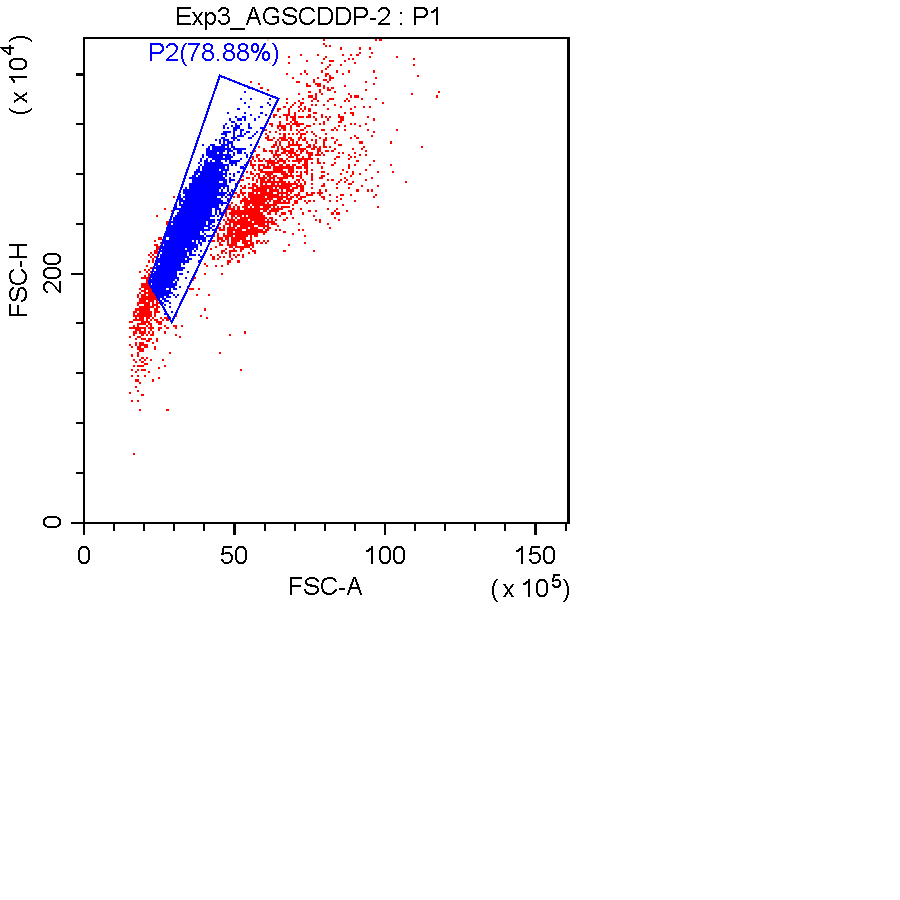

Supplement: Supplemental Information 1 [file peerj-11-15441-s001.zip › Raw data submitted/cell apoptosis/Fig. 10/AGSCDDP/Exp3_AGSCDDP-2_Plot2.bmp]

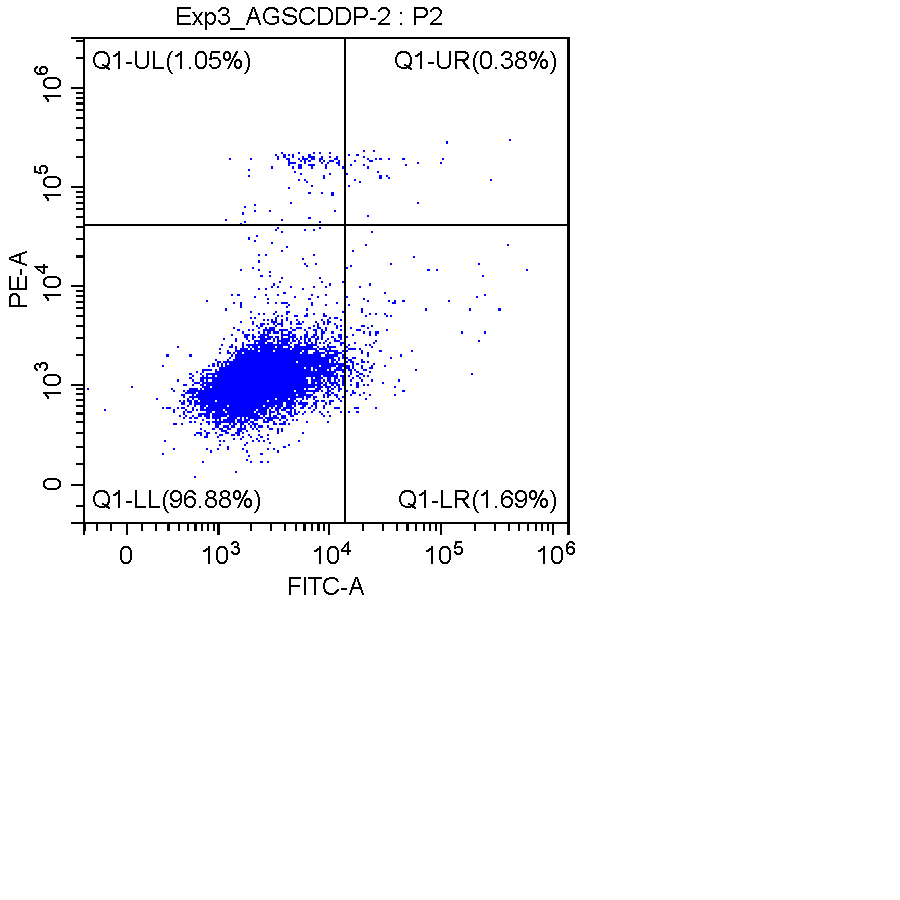

Supplement: Supplemental Information 1 [file peerj-11-15441-s001.zip › Raw data submitted/cell apoptosis/Fig. 10/AGSCDDP/Exp3_AGSCDDP-2_Plot3.bmp]

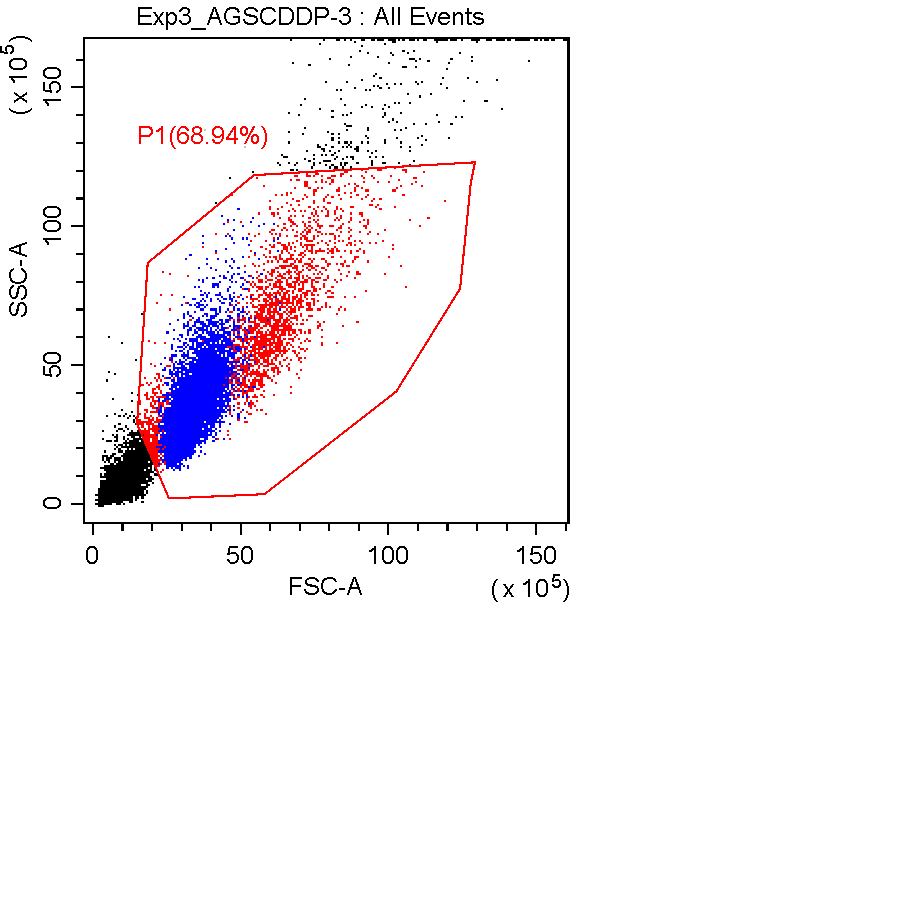

Supplement: Supplemental Information 1 [file peerj-11-15441-s001.zip › Raw data submitted/cell apoptosis/Fig. 10/AGSCDDP/Exp3_AGSCDDP-3_Plot1.bmp]

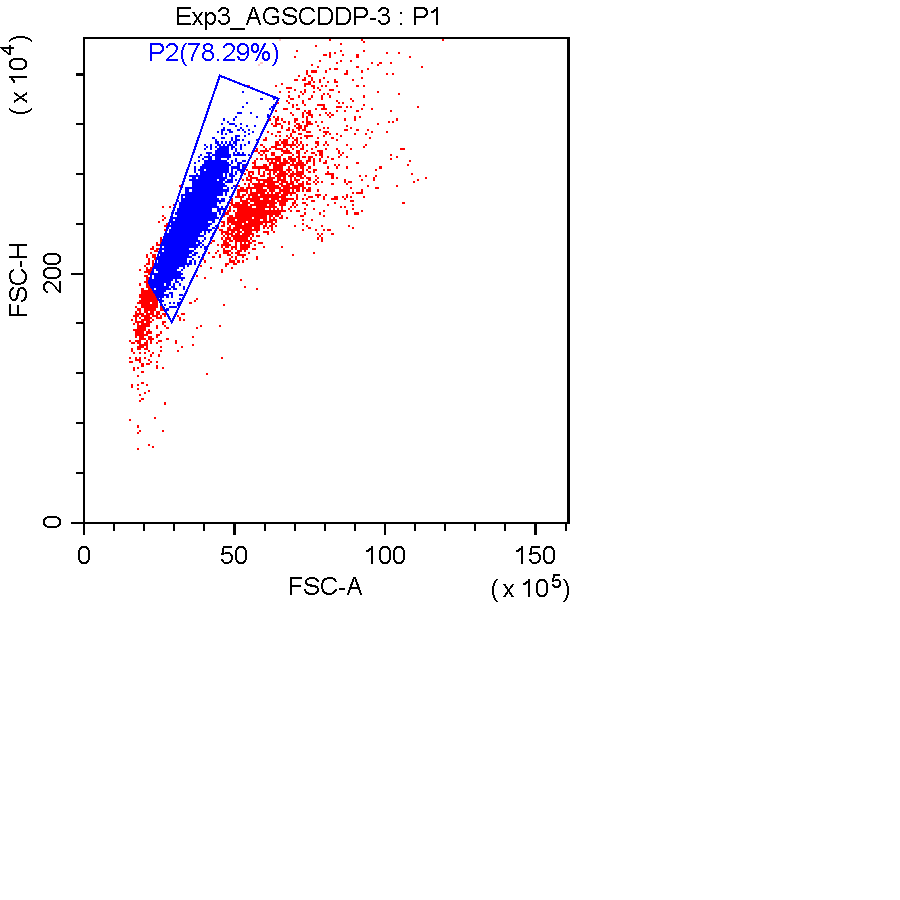

Supplement: Supplemental Information 1 [file peerj-11-15441-s001.zip › Raw data submitted/cell apoptosis/Fig. 10/AGSCDDP/Exp3_AGSCDDP-3_Plot2.bmp]

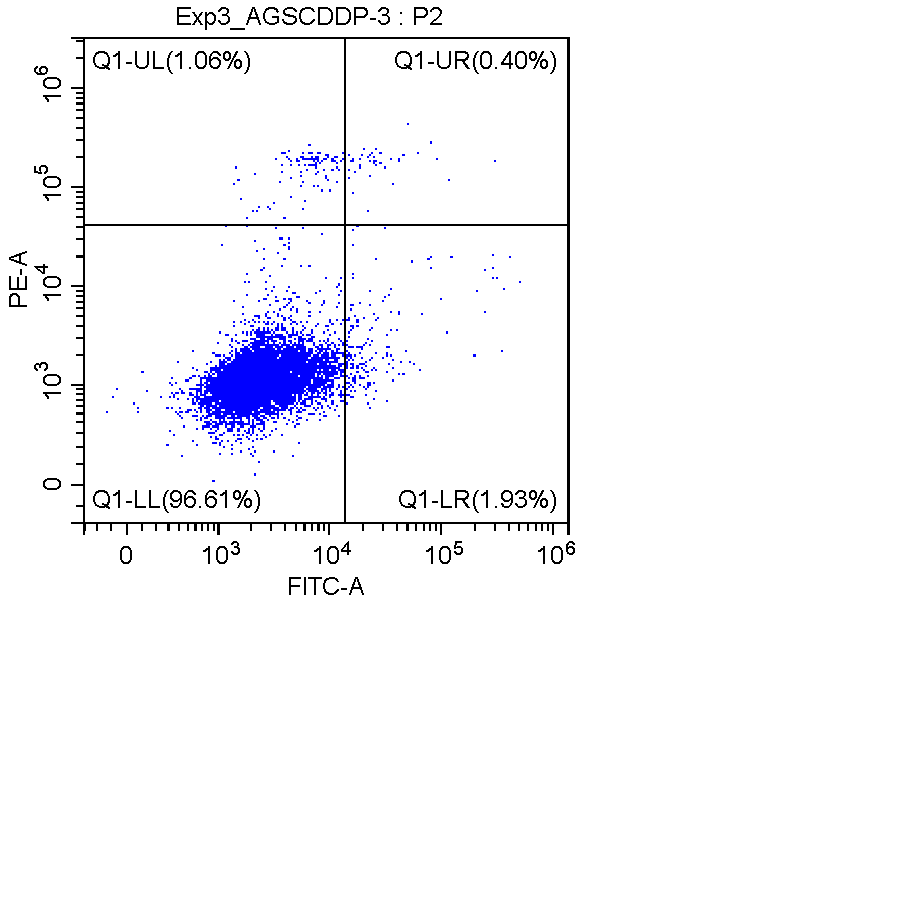

Supplement: Supplemental Information 1 [file peerj-11-15441-s001.zip › Raw data submitted/cell apoptosis/Fig. 10/AGSCDDP/Exp3_AGSCDDP-3_Plot3.bmp]

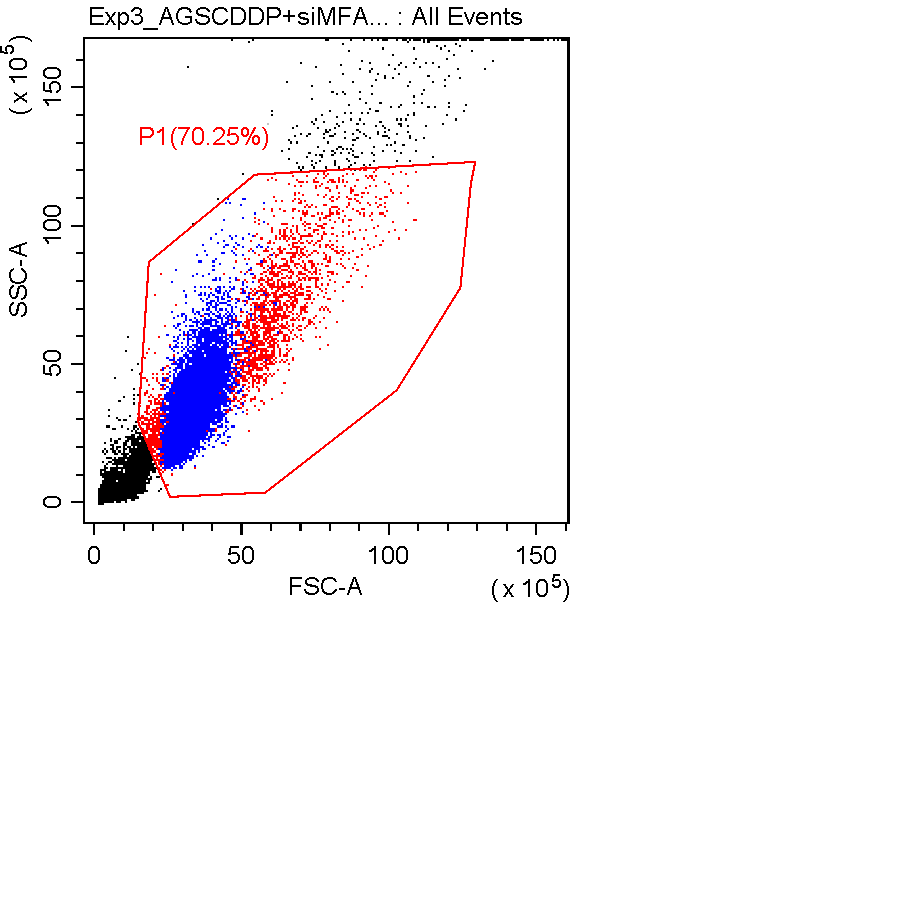

Supplement: Supplemental Information 1 [file peerj-11-15441-s001.zip › Raw data submitted/cell apoptosis/Fig. 10/AGSCDDP+siMFAP2+Rap/Exp3_AGSCDDP+siMFAP2+Rap-1_Plot1.bmp]

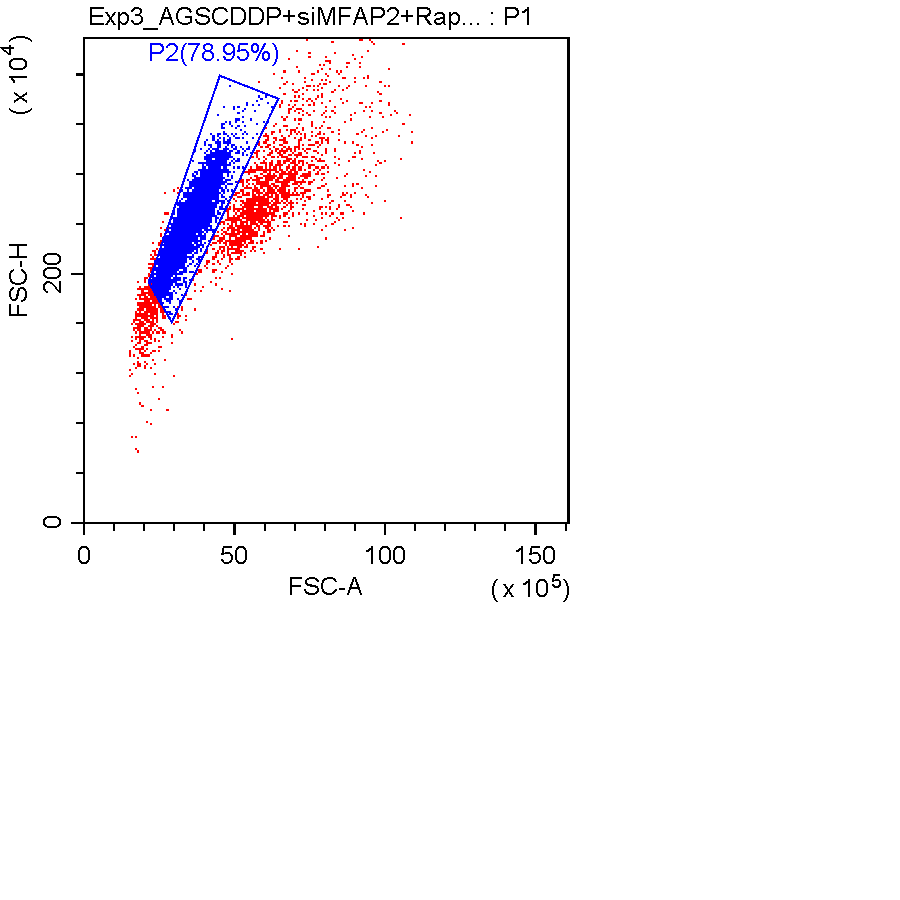

Supplement: Supplemental Information 1 [file peerj-11-15441-s001.zip › Raw data submitted/cell apoptosis/Fig. 10/AGSCDDP+siMFAP2+Rap/Exp3_AGSCDDP+siMFAP2+Rap-1_Plot2.bmp]

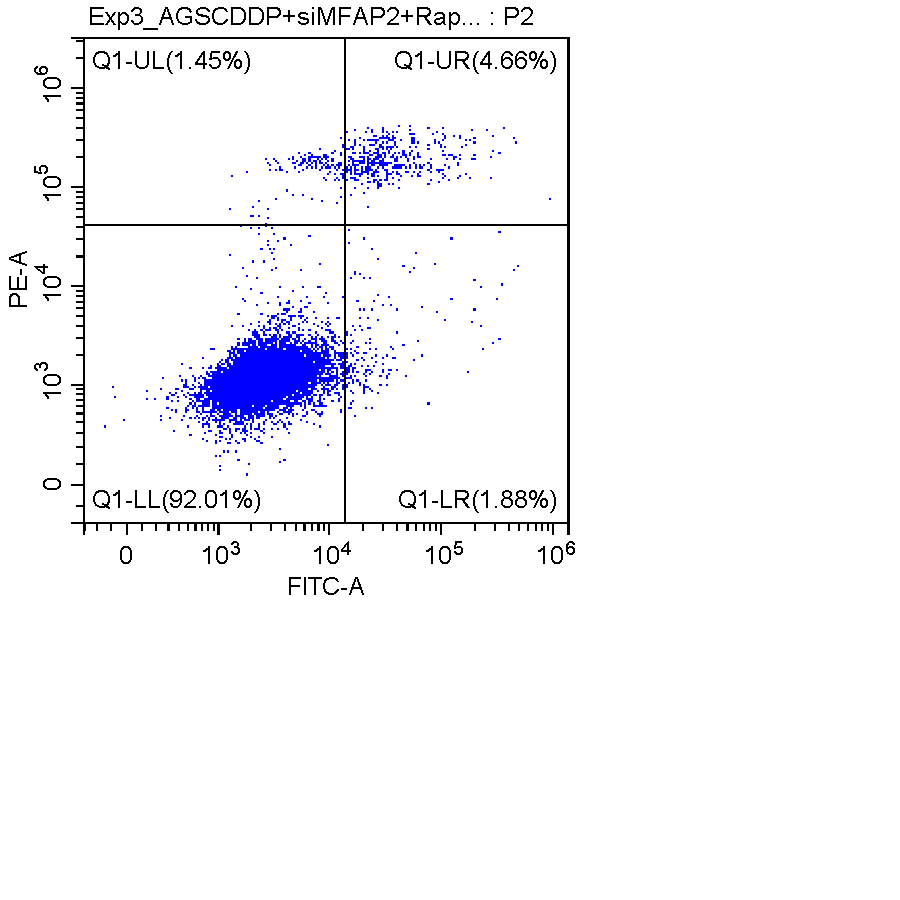

Supplement: Supplemental Information 1 [file peerj-11-15441-s001.zip › Raw data submitted/cell apoptosis/Fig. 10/AGSCDDP+siMFAP2+Rap/Exp3_AGSCDDP+siMFAP2+Rap-1_Plot3.bmp]

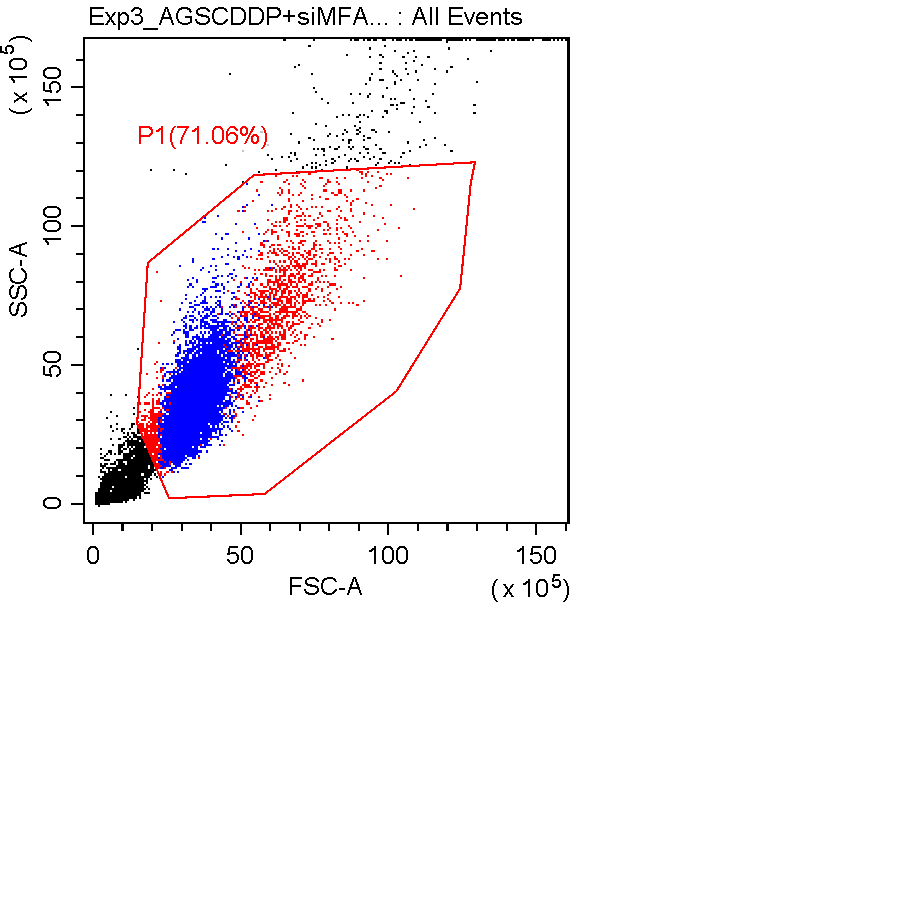

Supplement: Supplemental Information 1 [file peerj-11-15441-s001.zip › Raw data submitted/cell apoptosis/Fig. 10/AGSCDDP+siMFAP2+Rap/Exp3_AGSCDDP+siMFAP2+Rap-2_Plot1.bmp]

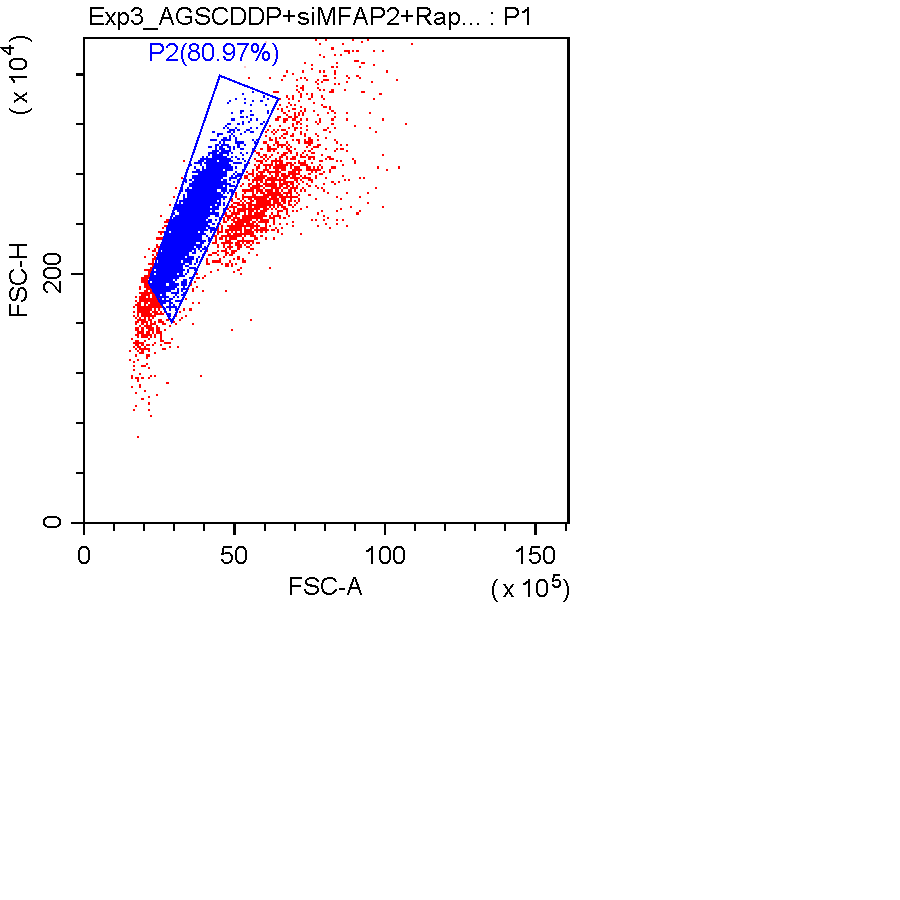

Supplement: Supplemental Information 1 [file peerj-11-15441-s001.zip › Raw data submitted/cell apoptosis/Fig. 10/AGSCDDP+siMFAP2+Rap/Exp3_AGSCDDP+siMFAP2+Rap-2_Plot2.bmp]

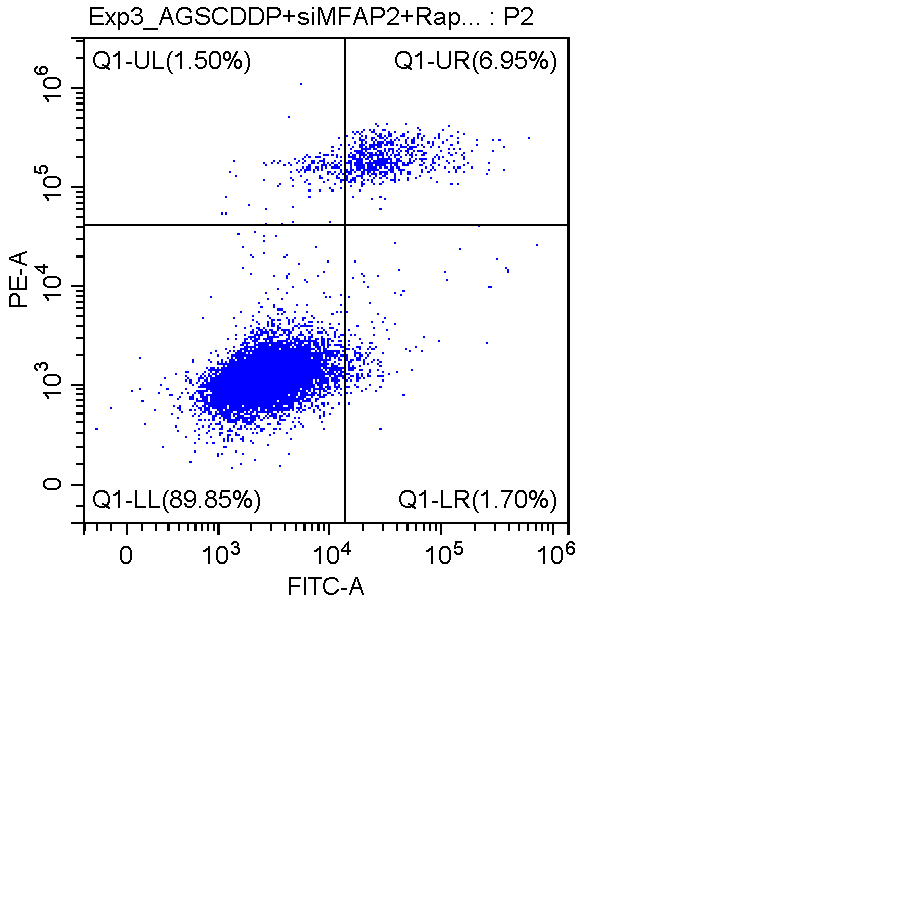

Supplement: Supplemental Information 1 [file peerj-11-15441-s001.zip › Raw data submitted/cell apoptosis/Fig. 10/AGSCDDP+siMFAP2+Rap/Exp3_AGSCDDP+siMFAP2+Rap-2_Plot3.bmp]

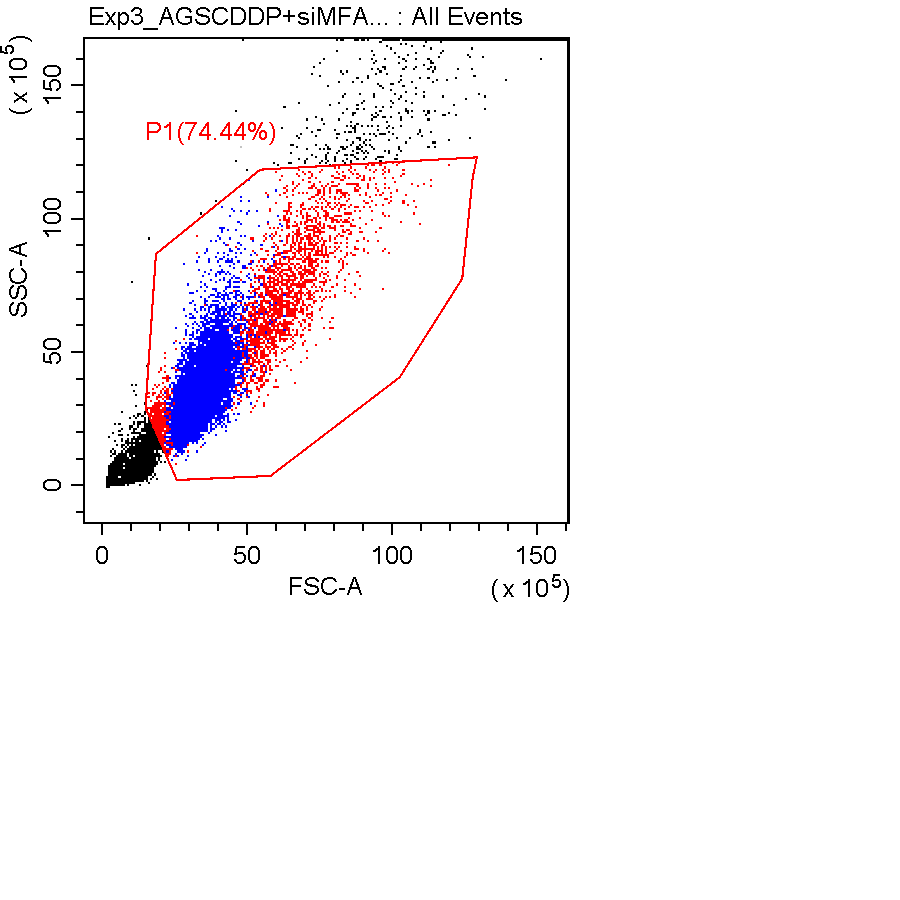

Supplement: Supplemental Information 1 [file peerj-11-15441-s001.zip › Raw data submitted/cell apoptosis/Fig. 10/AGSCDDP+siMFAP2+Rap/Exp3_AGSCDDP+siMFAP2+Rap-3_Plot1.bmp]

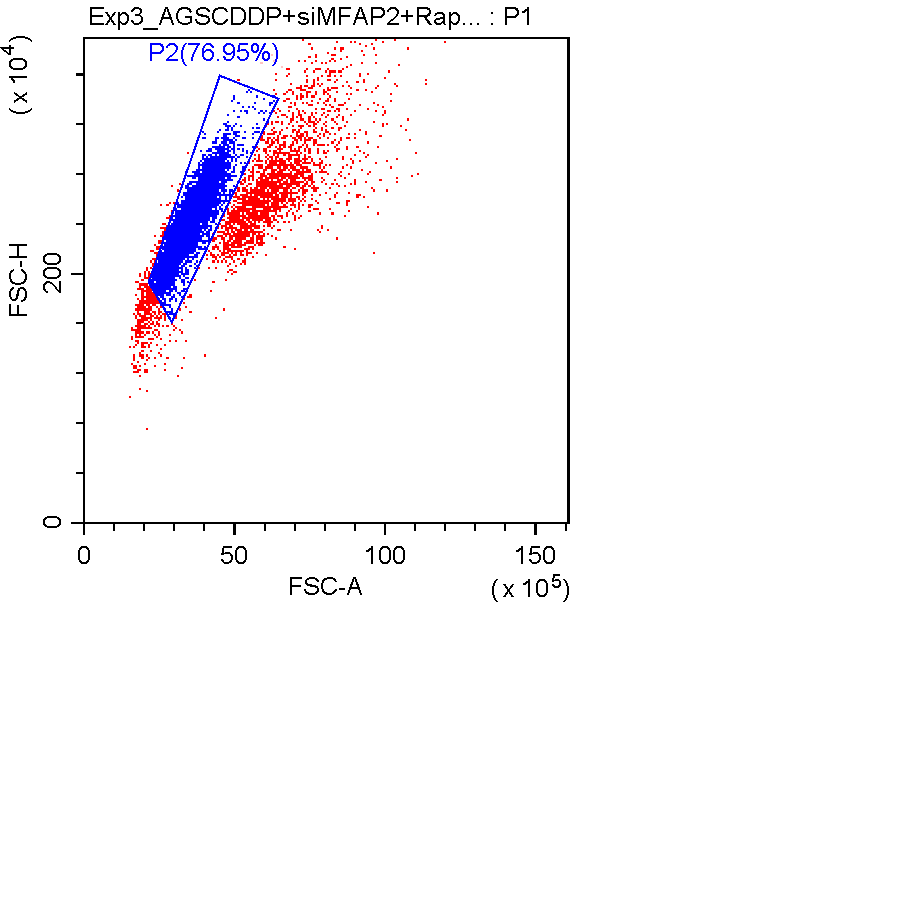

Supplement: Supplemental Information 1 [file peerj-11-15441-s001.zip › Raw data submitted/cell apoptosis/Fig. 10/AGSCDDP+siMFAP2+Rap/Exp3_AGSCDDP+siMFAP2+Rap-3_Plot2.bmp]

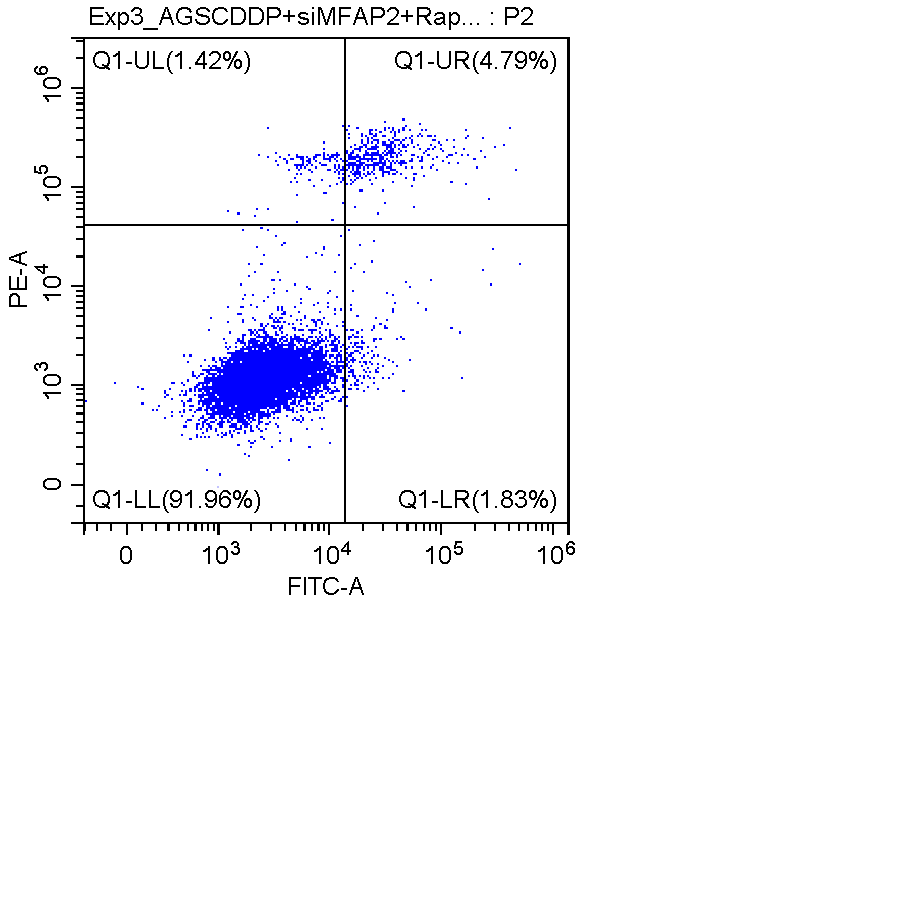

Supplement: Supplemental Information 1 [file peerj-11-15441-s001.zip › Raw data submitted/cell apoptosis/Fig. 10/AGSCDDP+siMFAP2+Rap/Exp3_AGSCDDP+siMFAP2+Rap-3_Plot3.bmp]

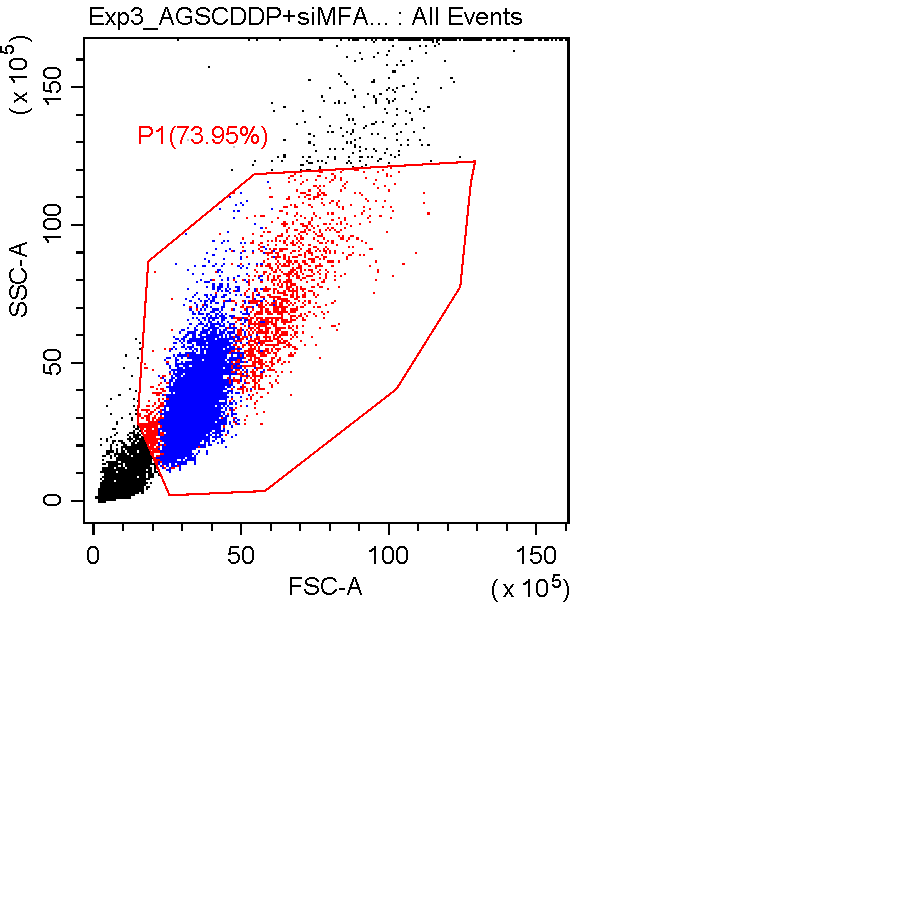

Supplement: Supplemental Information 1 [file peerj-11-15441-s001.zip › Raw data submitted/cell apoptosis/Fig. 10/Exp3_AGSCDDP+siMFAP2/Exp3_AGSCDDP+siMFAP2-1_Plot1.bmp]

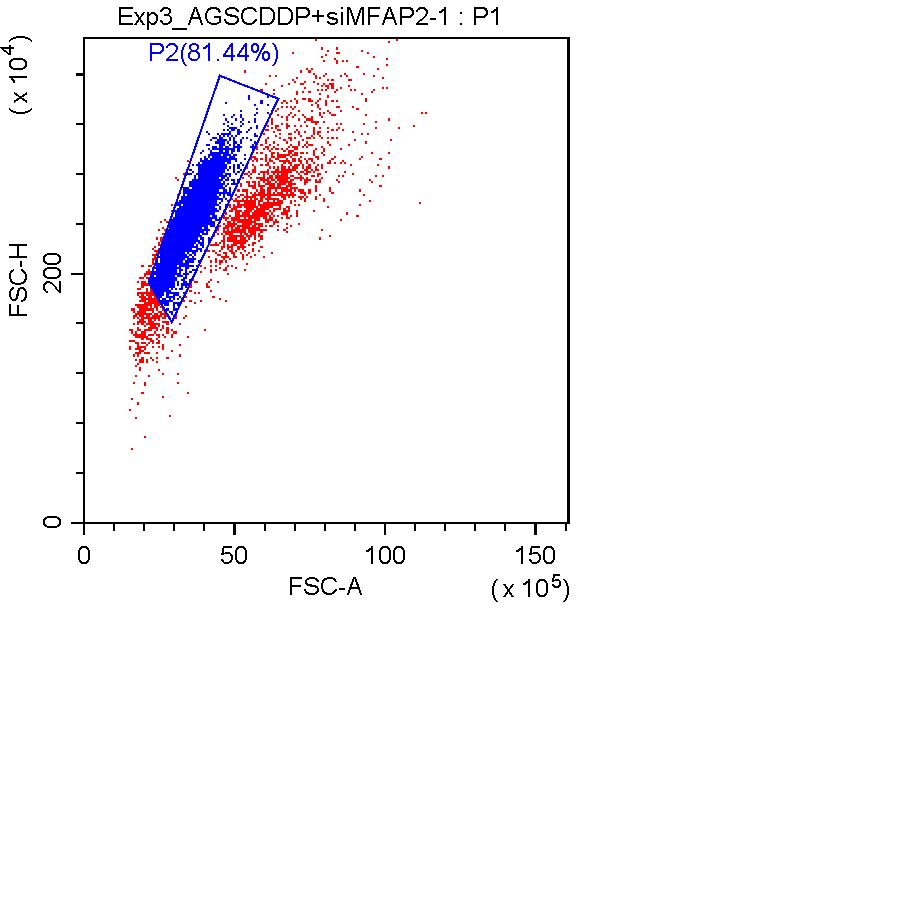

Supplement: Supplemental Information 1 [file peerj-11-15441-s001.zip › Raw data submitted/cell apoptosis/Fig. 10/Exp3_AGSCDDP+siMFAP2/Exp3_AGSCDDP+siMFAP2-1_Plot2.bmp]

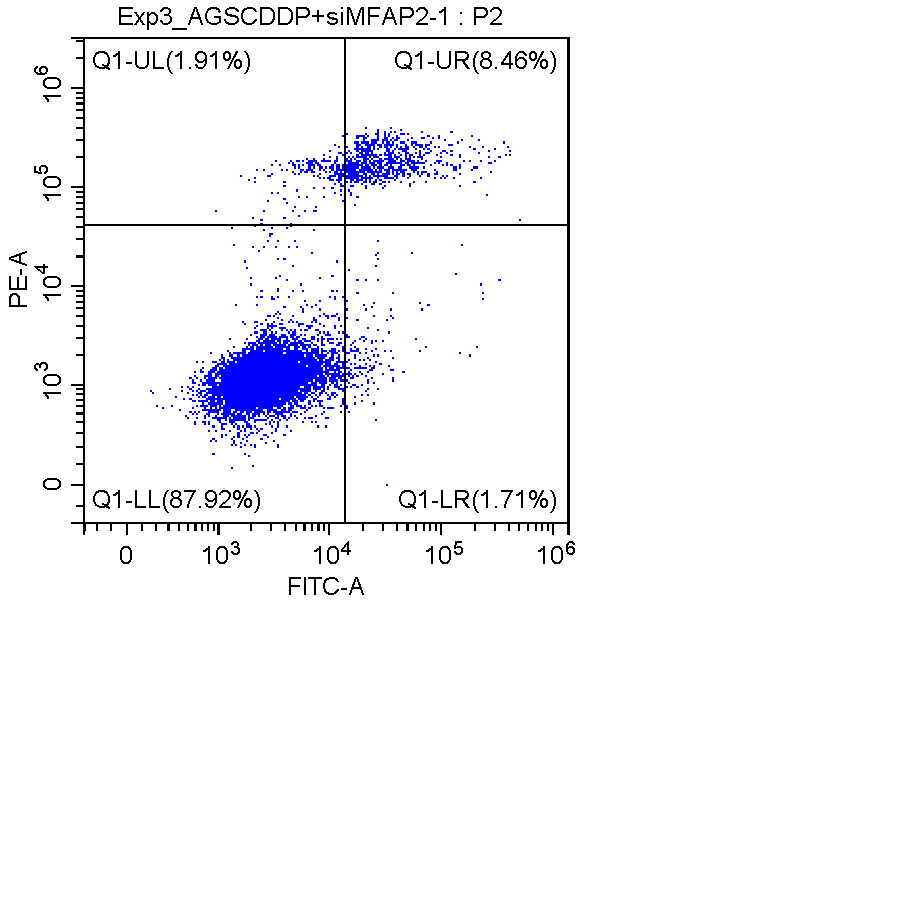

Supplement: Supplemental Information 1 [file peerj-11-15441-s001.zip › Raw data submitted/cell apoptosis/Fig. 10/Exp3_AGSCDDP+siMFAP2/Exp3_AGSCDDP+siMFAP2-1_Plot3.bmp]

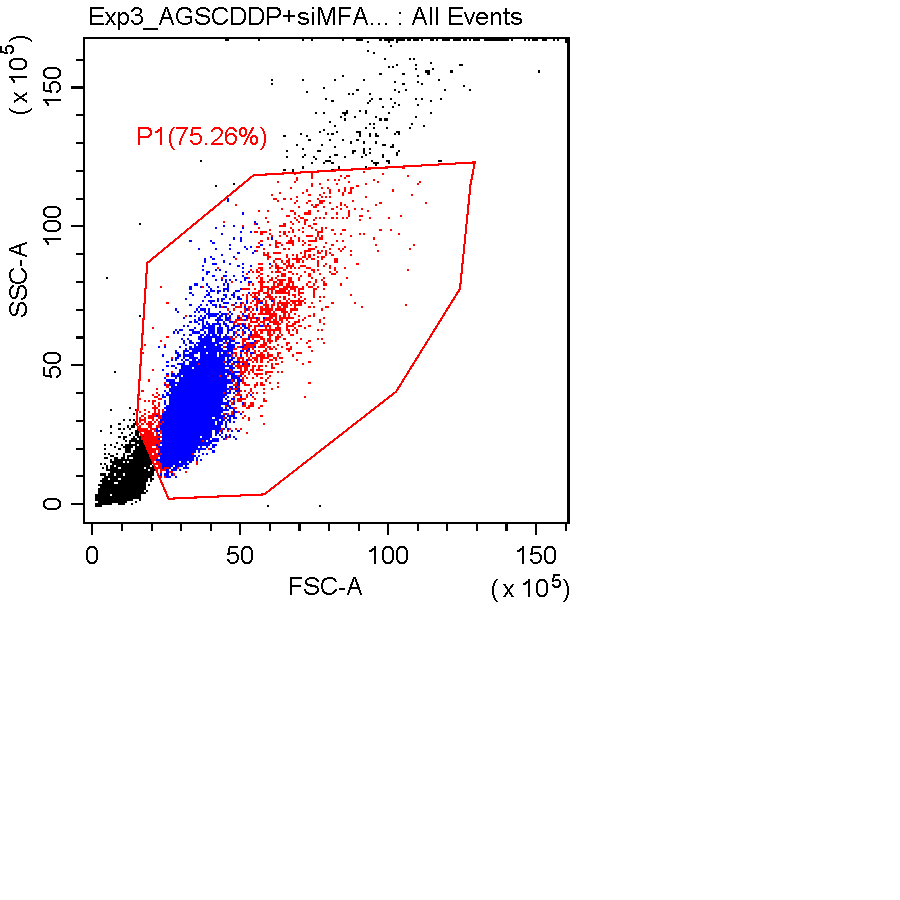

Supplement: Supplemental Information 1 [file peerj-11-15441-s001.zip › Raw data submitted/cell apoptosis/Fig. 10/Exp3_AGSCDDP+siMFAP2/Exp3_AGSCDDP+siMFAP2-2_Plot1.bmp]

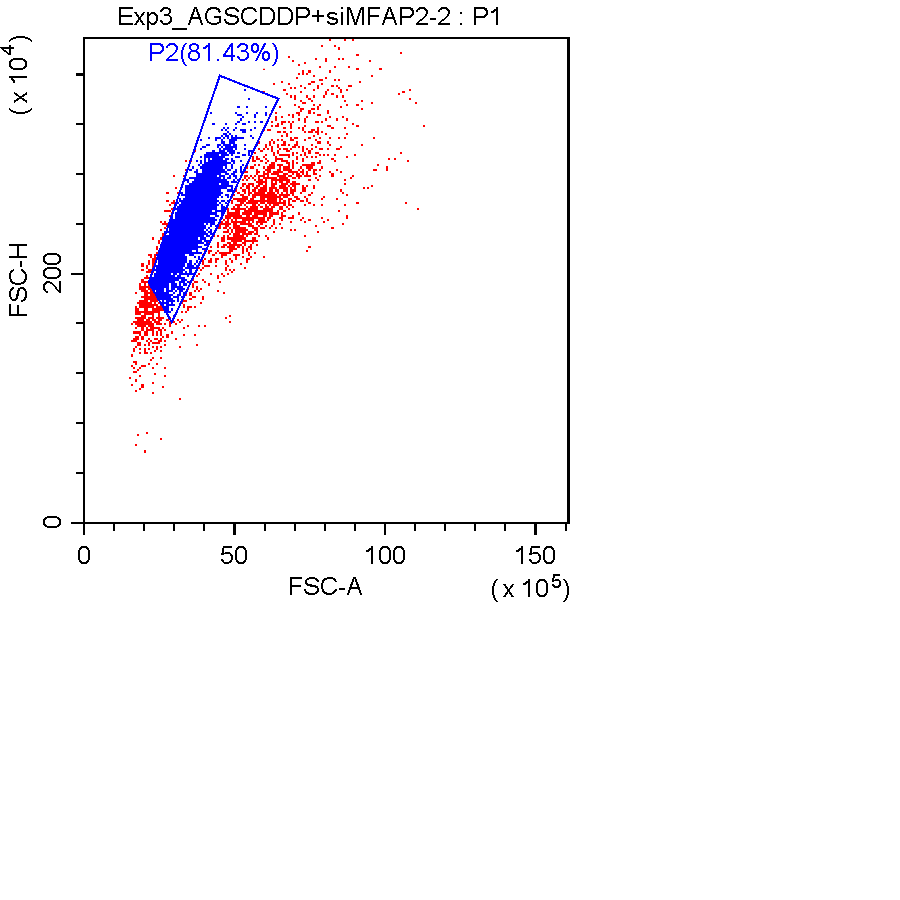

Supplement: Supplemental Information 1 [file peerj-11-15441-s001.zip › Raw data submitted/cell apoptosis/Fig. 10/Exp3_AGSCDDP+siMFAP2/Exp3_AGSCDDP+siMFAP2-2_Plot2.bmp]

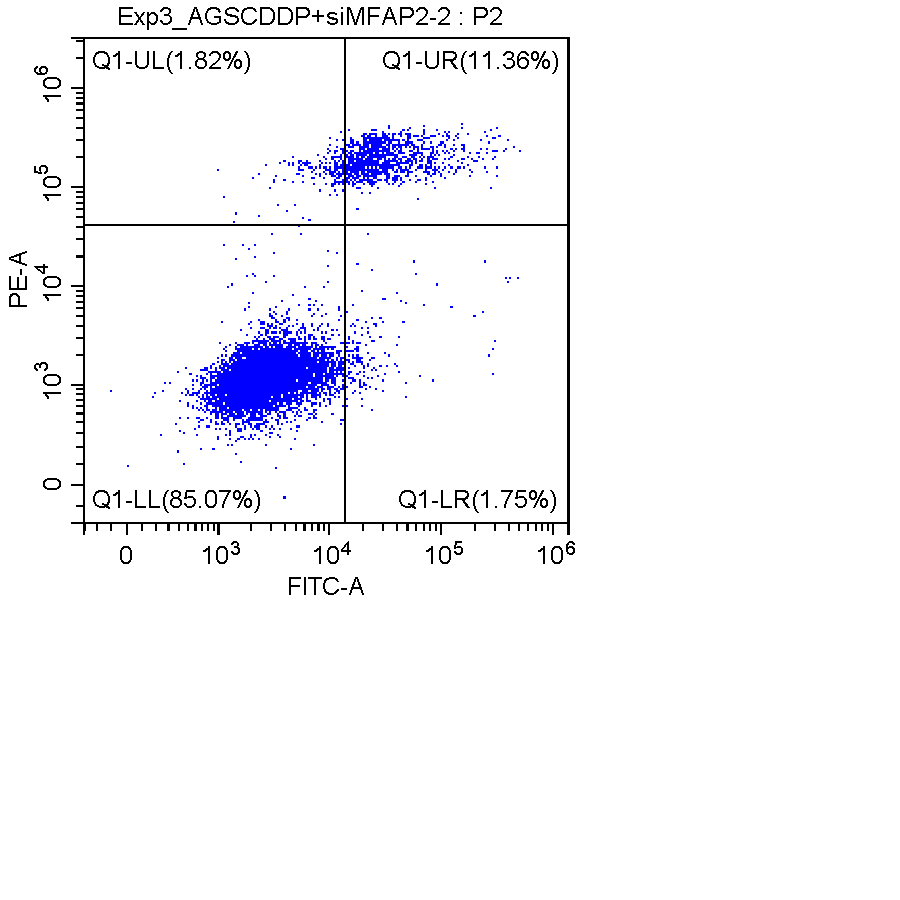

Supplement: Supplemental Information 1 [file peerj-11-15441-s001.zip › Raw data submitted/cell apoptosis/Fig. 10/Exp3_AGSCDDP+siMFAP2/Exp3_AGSCDDP+siMFAP2-2_Plot3.bmp]

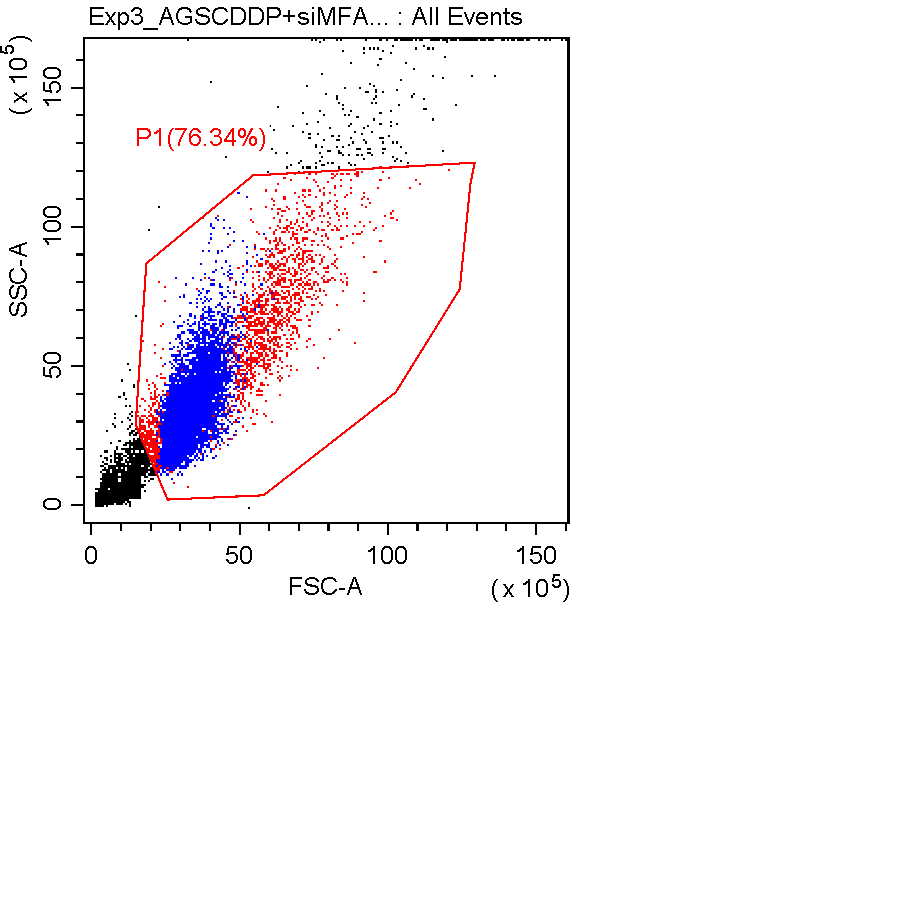

Supplement: Supplemental Information 1 [file peerj-11-15441-s001.zip › Raw data submitted/cell apoptosis/Fig. 10/Exp3_AGSCDDP+siMFAP2/Exp3_AGSCDDP+siMFAP2-3_Plot1.bmp]

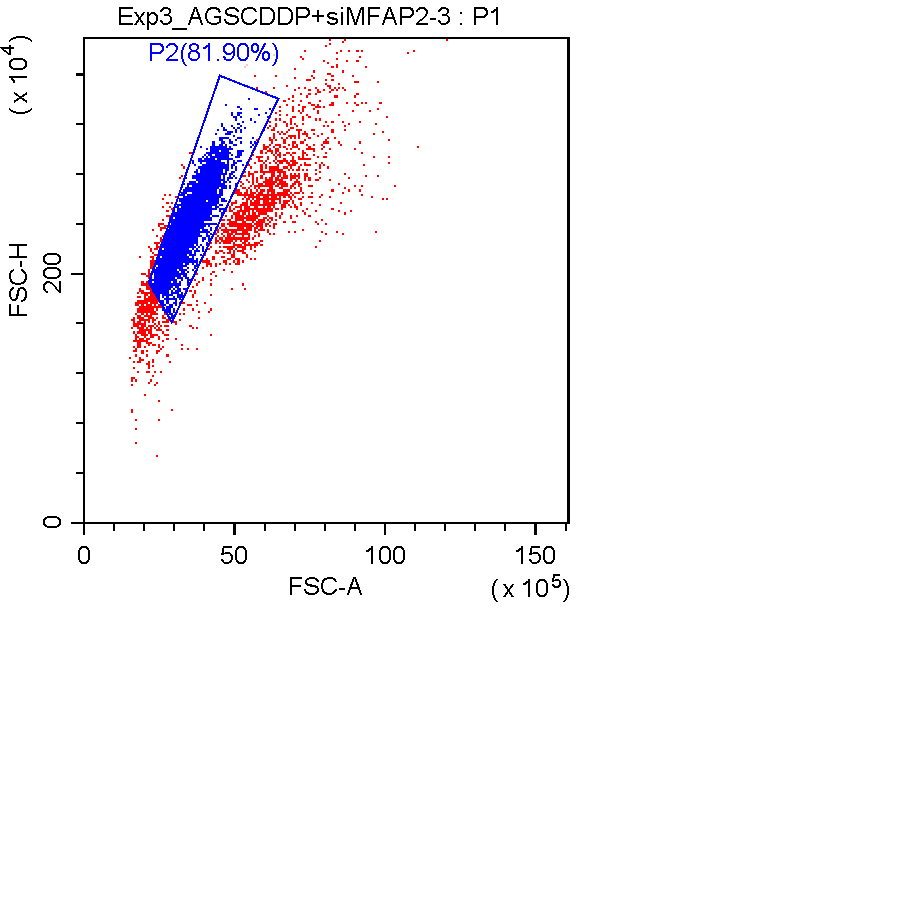

Supplement: Supplemental Information 1 [file peerj-11-15441-s001.zip › Raw data submitted/cell apoptosis/Fig. 10/Exp3_AGSCDDP+siMFAP2/Exp3_AGSCDDP+siMFAP2-3_Plot2.bmp]

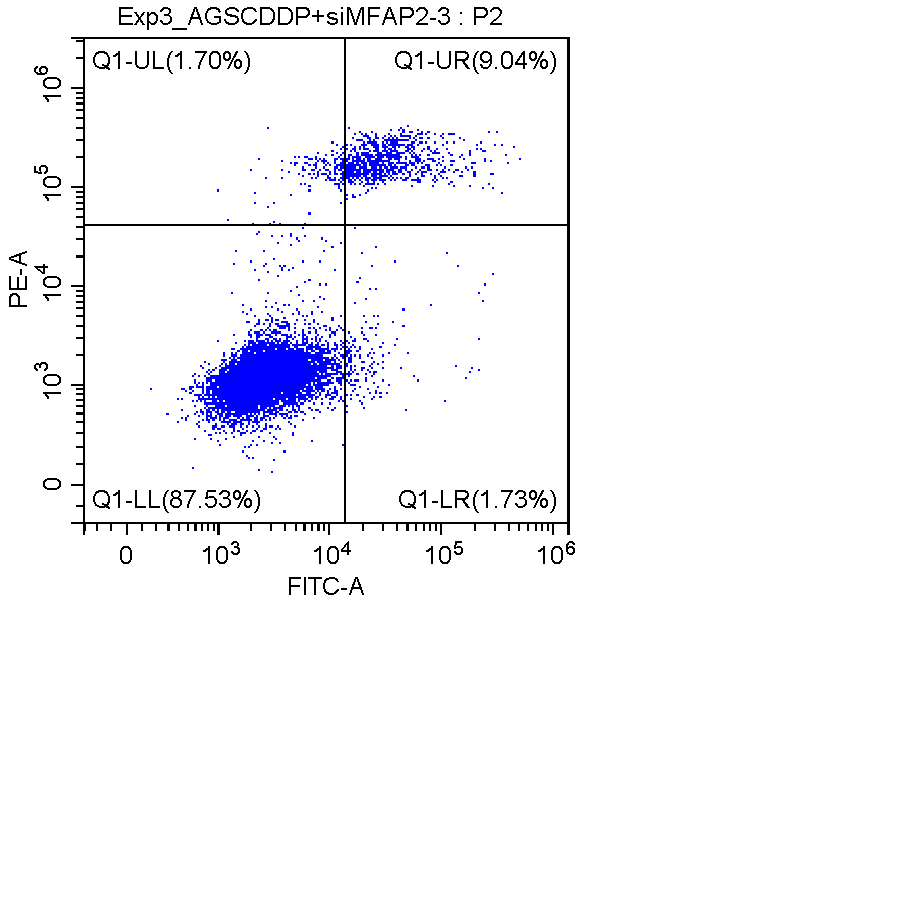

Supplement: Supplemental Information 1 [file peerj-11-15441-s001.zip › Raw data submitted/cell apoptosis/Fig. 10/Exp3_AGSCDDP+siMFAP2/Exp3_AGSCDDP+siMFAP2-3_Plot3.bmp]

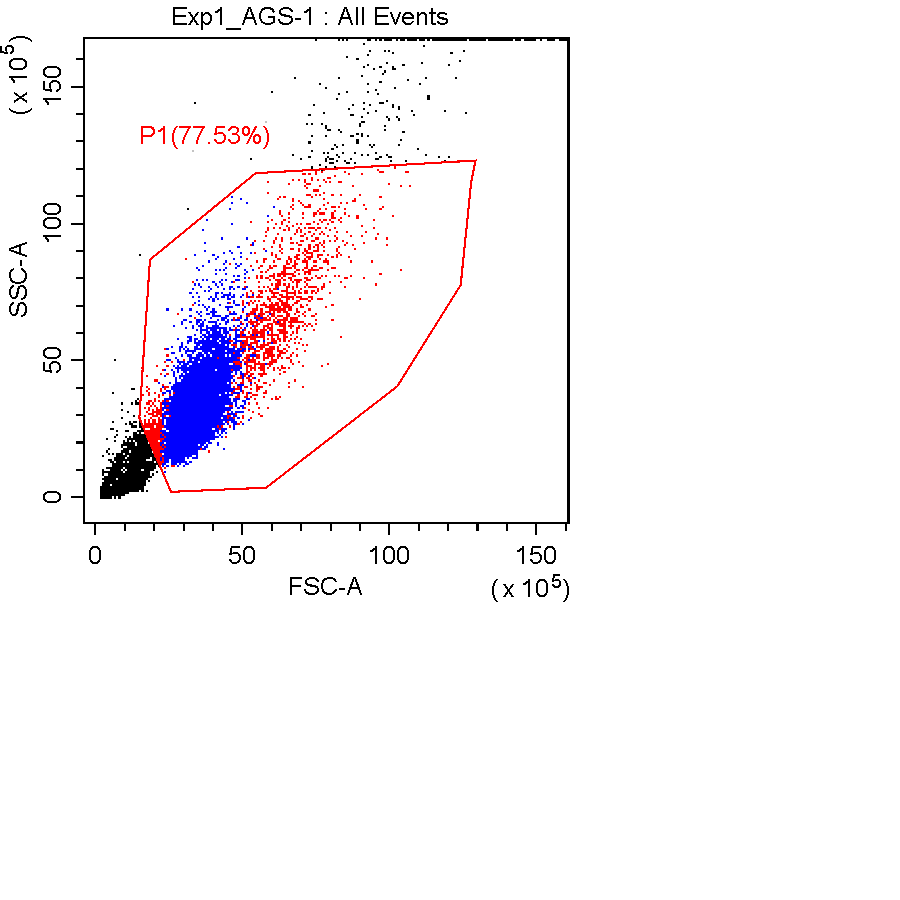

Supplement: Supplemental Information 1 [file peerj-11-15441-s001.zip › Raw data submitted/cell apoptosis/Fig. 7/AGS/Exp1_AGS-1_Plot1.bmp]

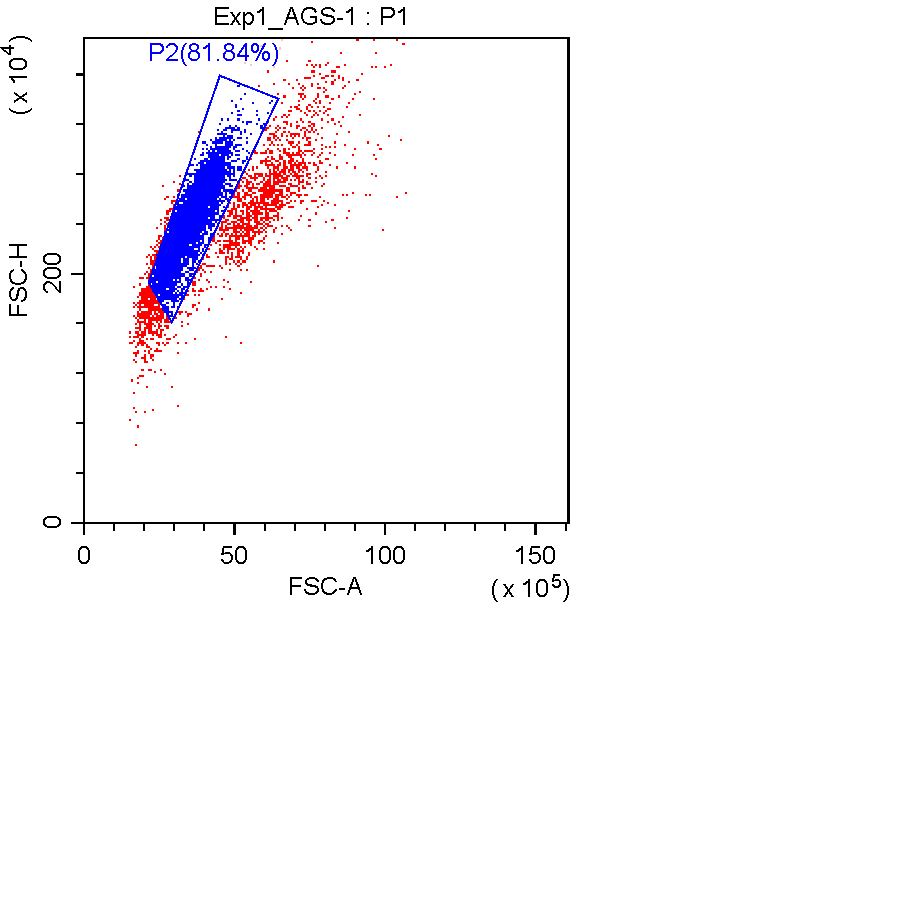

Supplement: Supplemental Information 1 [file peerj-11-15441-s001.zip › Raw data submitted/cell apoptosis/Fig. 7/AGS/Exp1_AGS-1_Plot2.bmp]

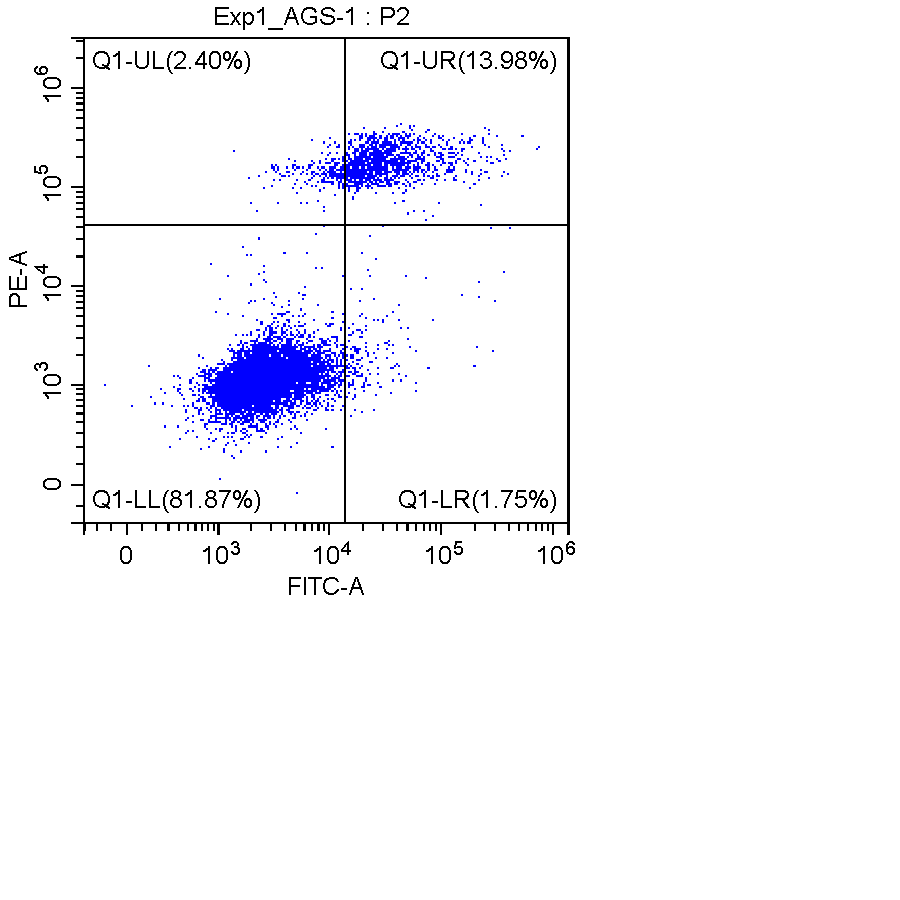

Supplement: Supplemental Information 1 [file peerj-11-15441-s001.zip › Raw data submitted/cell apoptosis/Fig. 7/AGS/Exp1_AGS-1_Plot3.bmp]

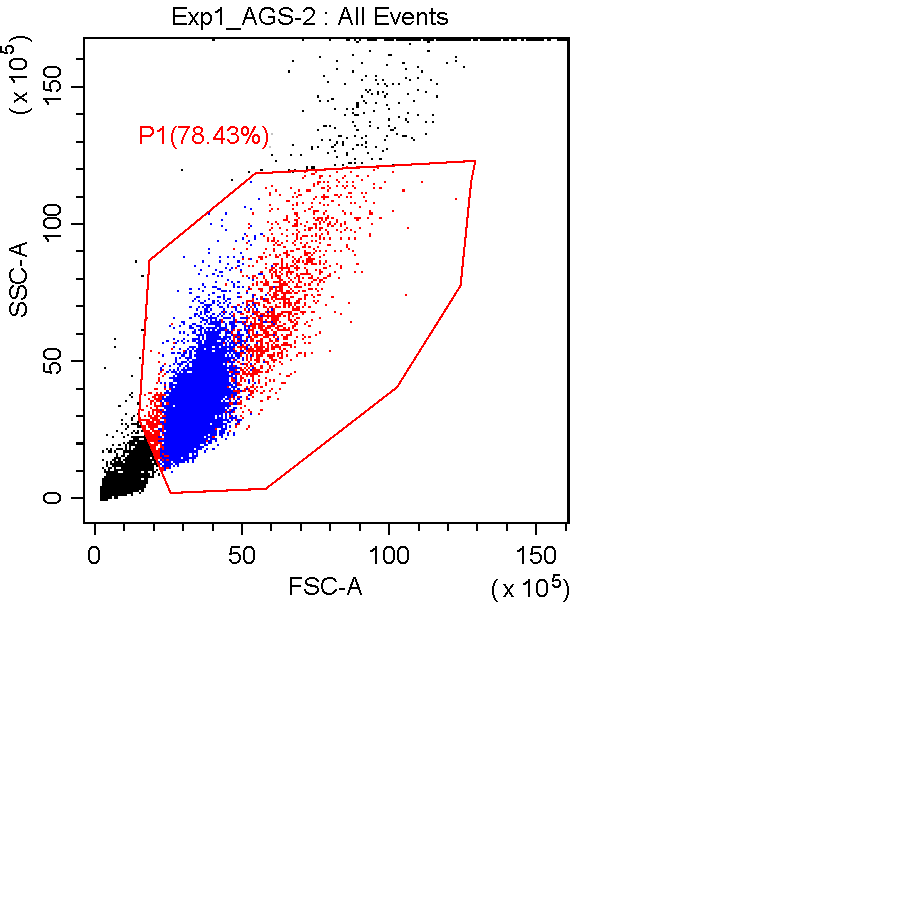

Supplement: Supplemental Information 1 [file peerj-11-15441-s001.zip › Raw data submitted/cell apoptosis/Fig. 7/AGS/Exp1_AGS-2_Plot1.bmp]

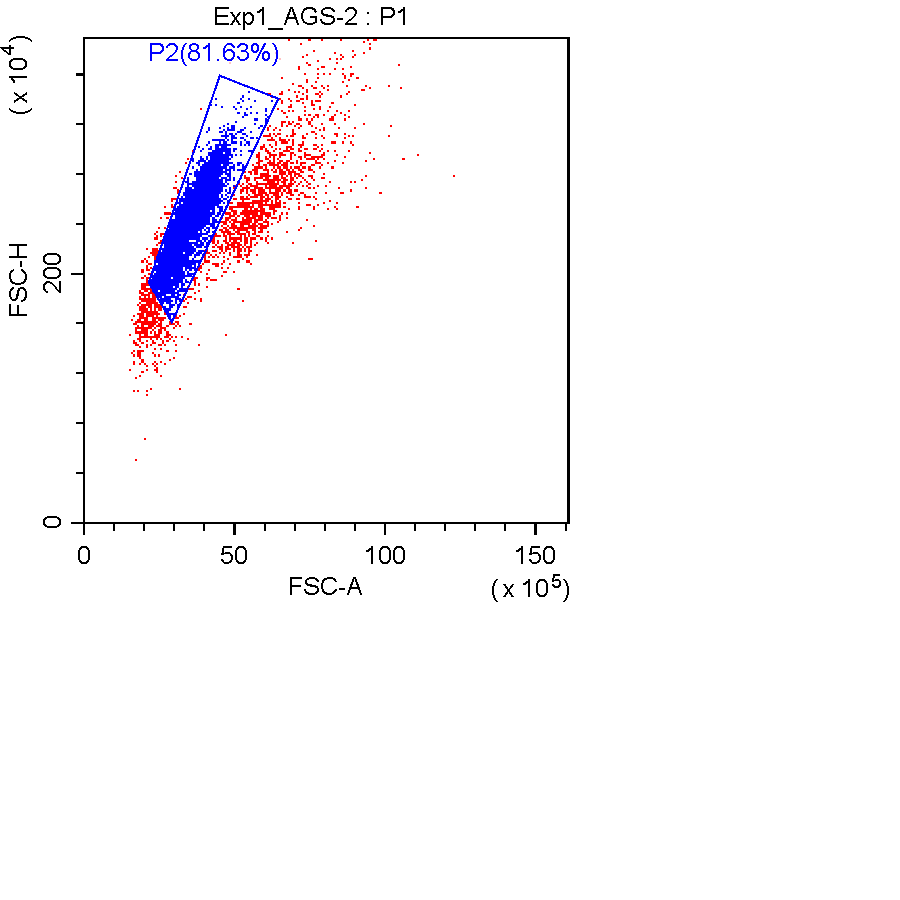

Supplement: Supplemental Information 1 [file peerj-11-15441-s001.zip › Raw data submitted/cell apoptosis/Fig. 7/AGS/Exp1_AGS-2_Plot2.bmp]

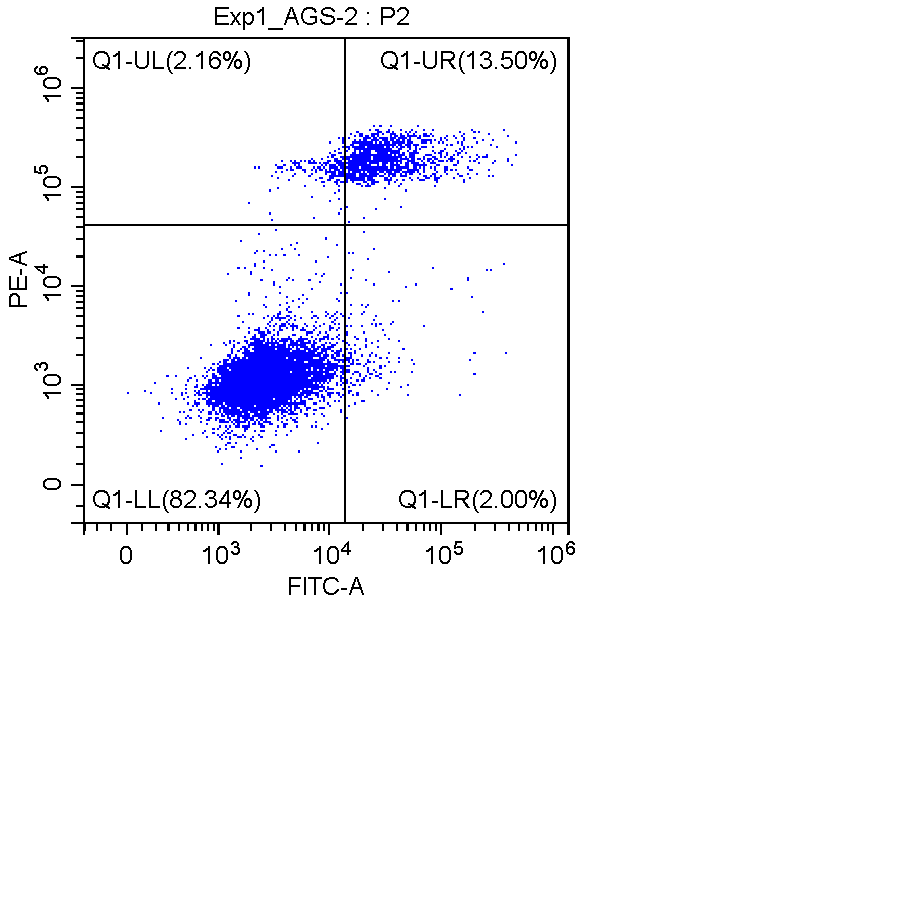

Supplement: Supplemental Information 1 [file peerj-11-15441-s001.zip › Raw data submitted/cell apoptosis/Fig. 7/AGS/Exp1_AGS-2_Plot3.bmp]

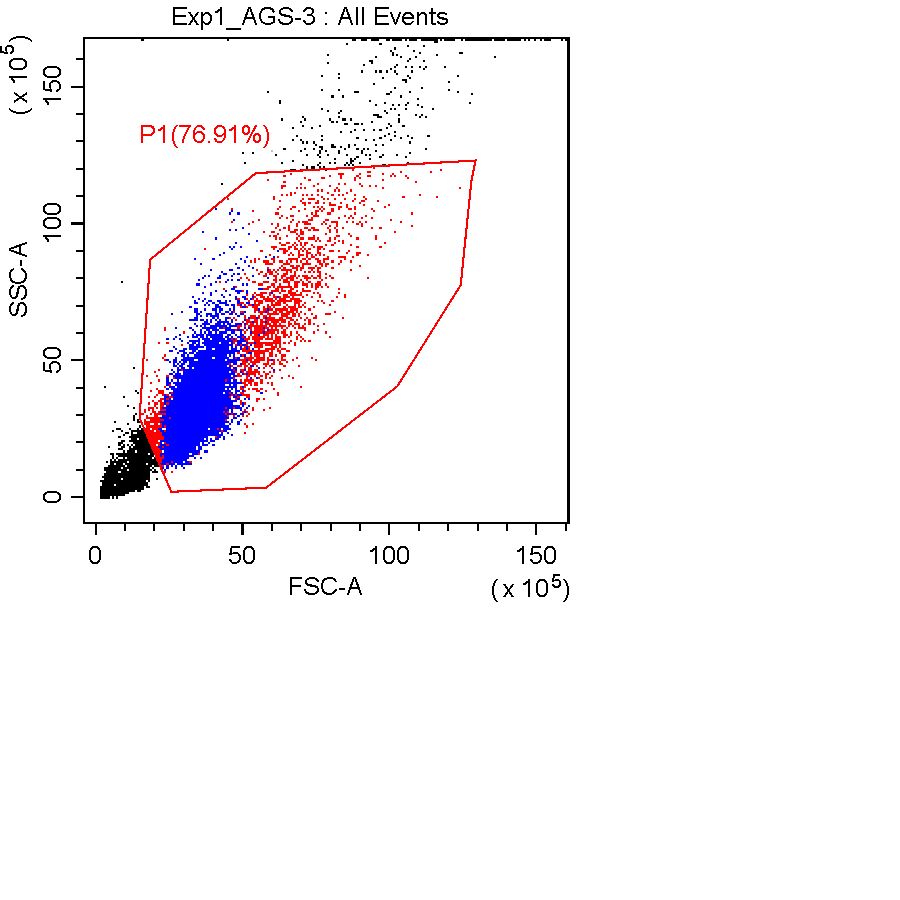

Supplement: Supplemental Information 1 [file peerj-11-15441-s001.zip › Raw data submitted/cell apoptosis/Fig. 7/AGS/Exp1_AGS-3_Plot1.bmp]

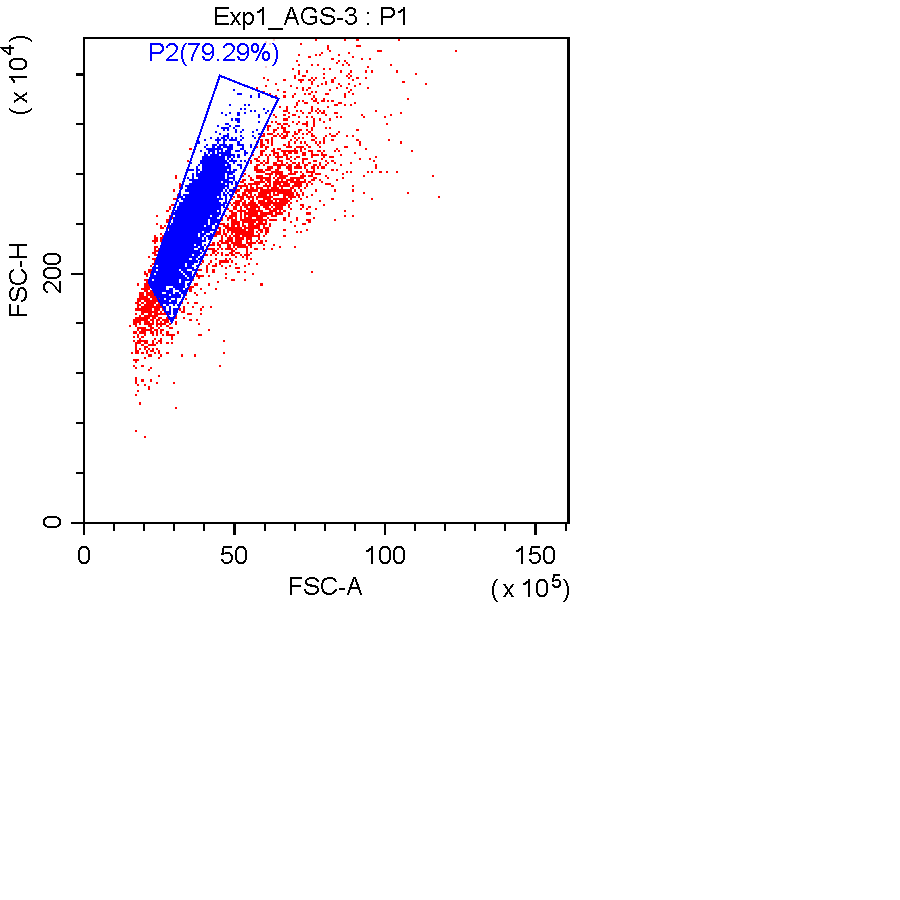

Supplement: Supplemental Information 1 [file peerj-11-15441-s001.zip › Raw data submitted/cell apoptosis/Fig. 7/AGS/Exp1_AGS-3_Plot2.bmp]

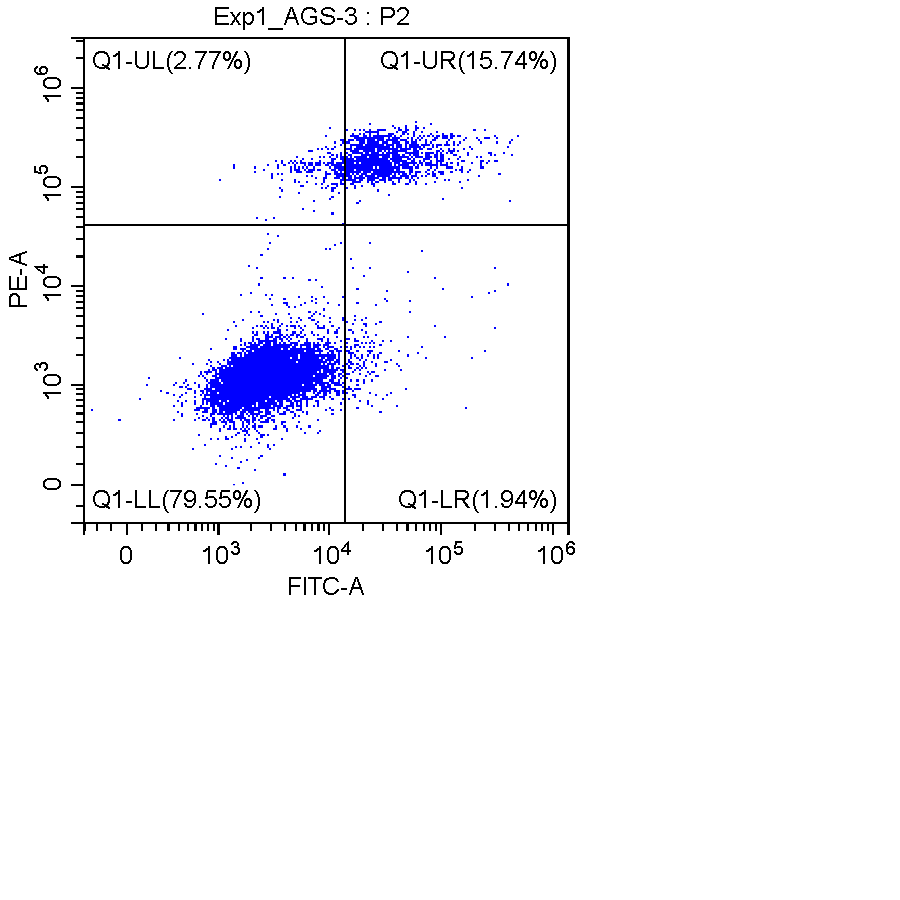

Supplement: Supplemental Information 1 [file peerj-11-15441-s001.zip › Raw data submitted/cell apoptosis/Fig. 7/AGS/Exp1_AGS-3_Plot3.bmp]

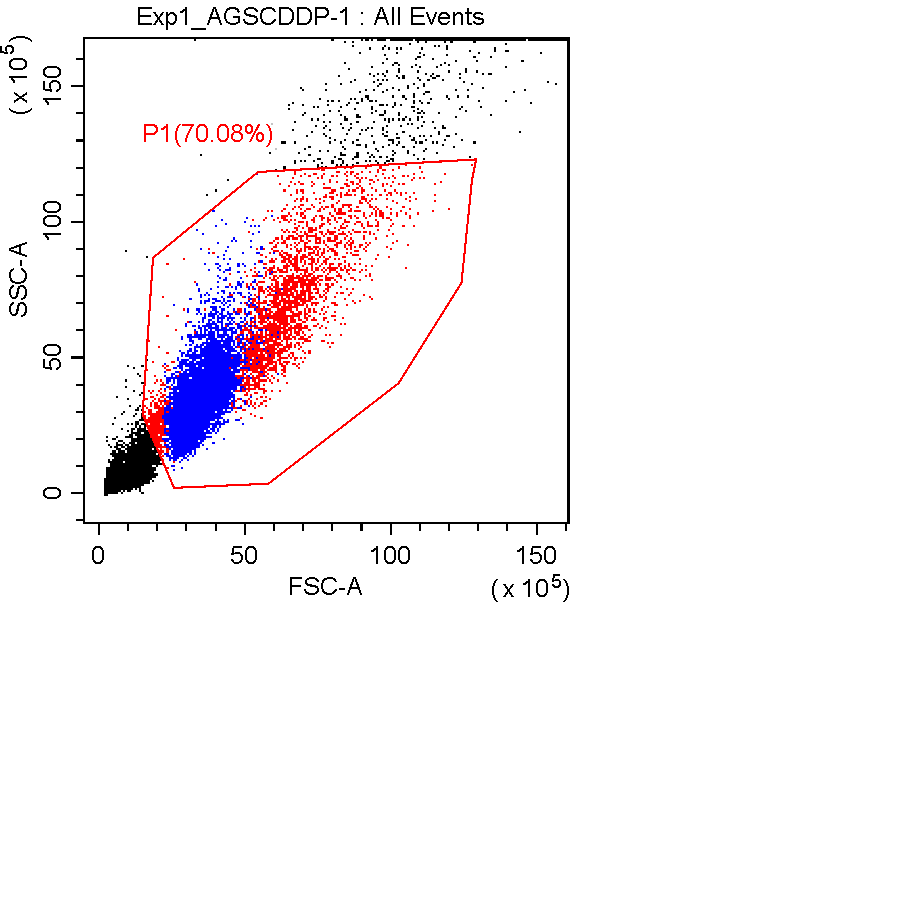

Supplement: Supplemental Information 1 [file peerj-11-15441-s001.zip › Raw data submitted/cell apoptosis/Fig. 7/AGSCDDP/Exp1_AGSCDDP-1_Plot1.bmp]

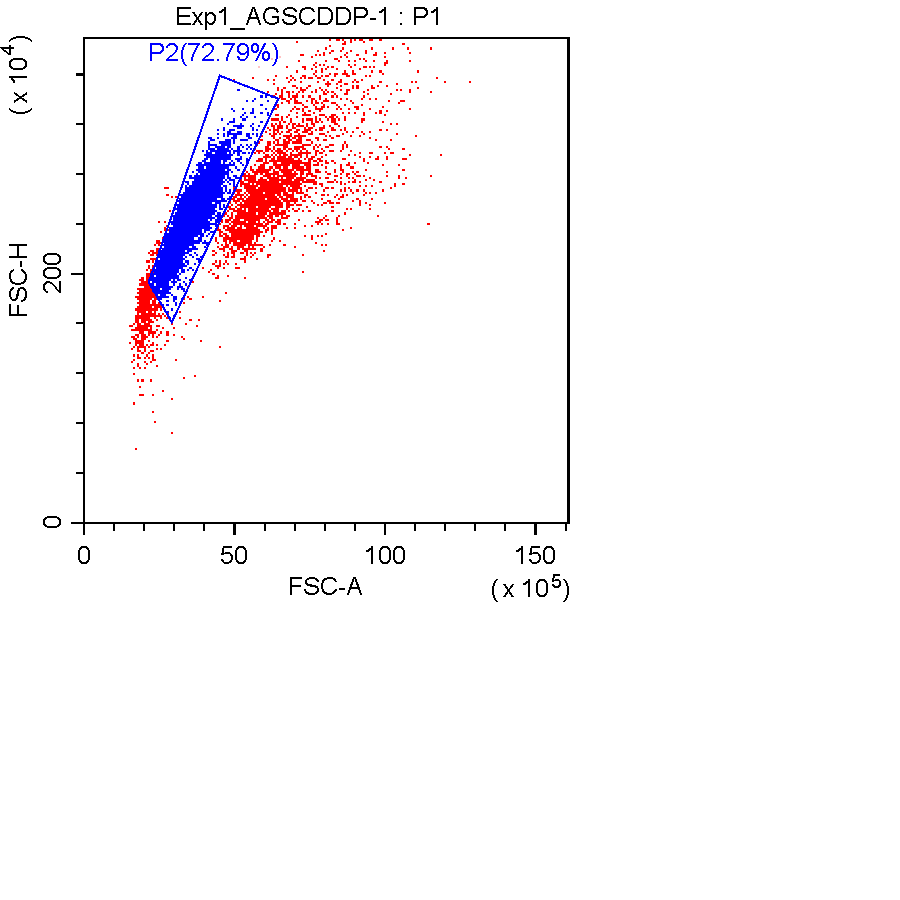

Supplement: Supplemental Information 1 [file peerj-11-15441-s001.zip › Raw data submitted/cell apoptosis/Fig. 7/AGSCDDP/Exp1_AGSCDDP-1_Plot2.bmp]

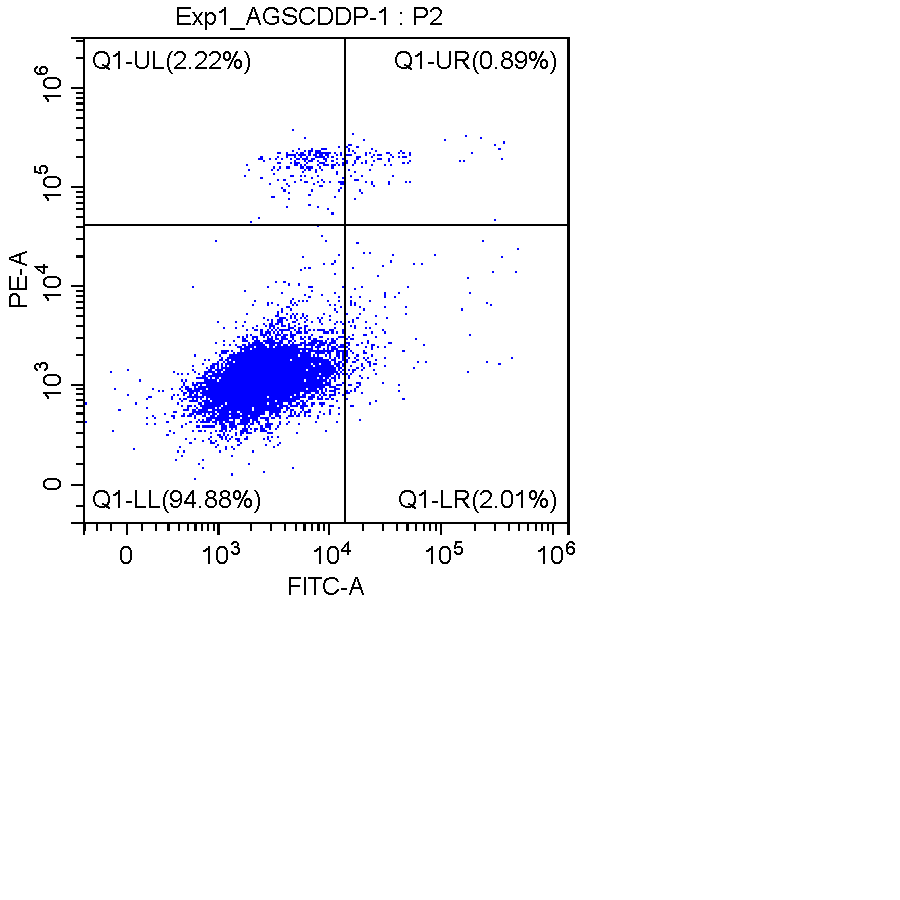

Supplement: Supplemental Information 1 [file peerj-11-15441-s001.zip › Raw data submitted/cell apoptosis/Fig. 7/AGSCDDP/Exp1_AGSCDDP-1_Plot3.bmp]

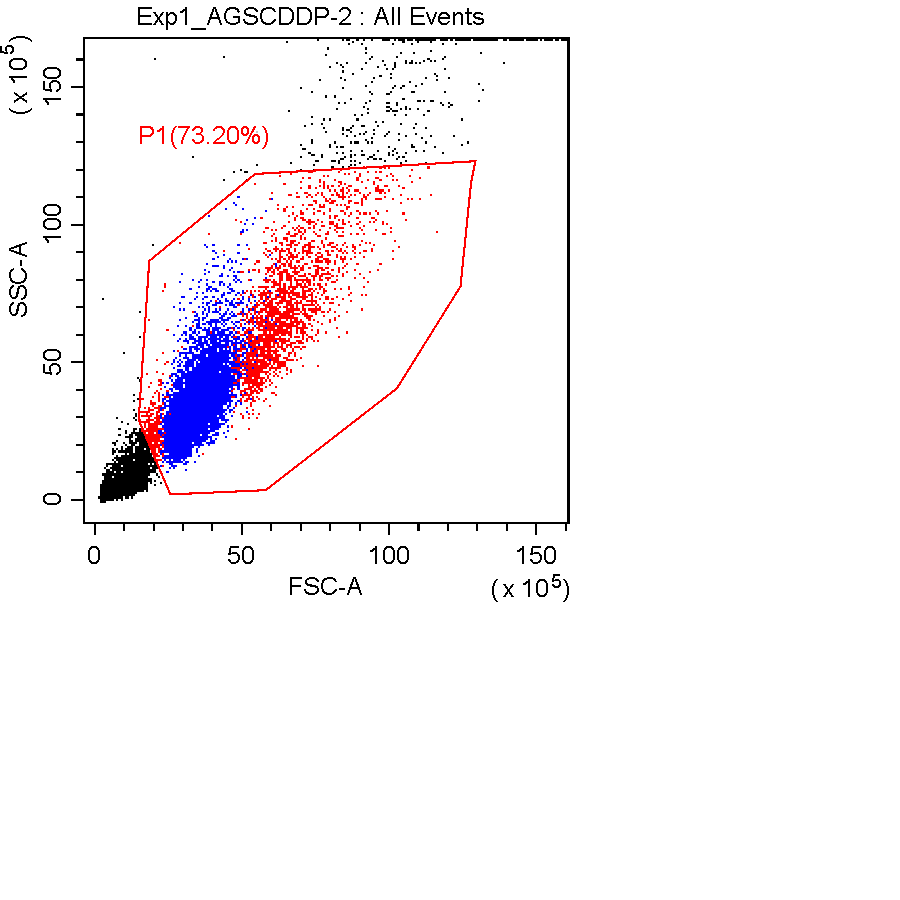

Supplement: Supplemental Information 1 [file peerj-11-15441-s001.zip › Raw data submitted/cell apoptosis/Fig. 7/AGSCDDP/Exp1_AGSCDDP-2_Plot1.bmp]

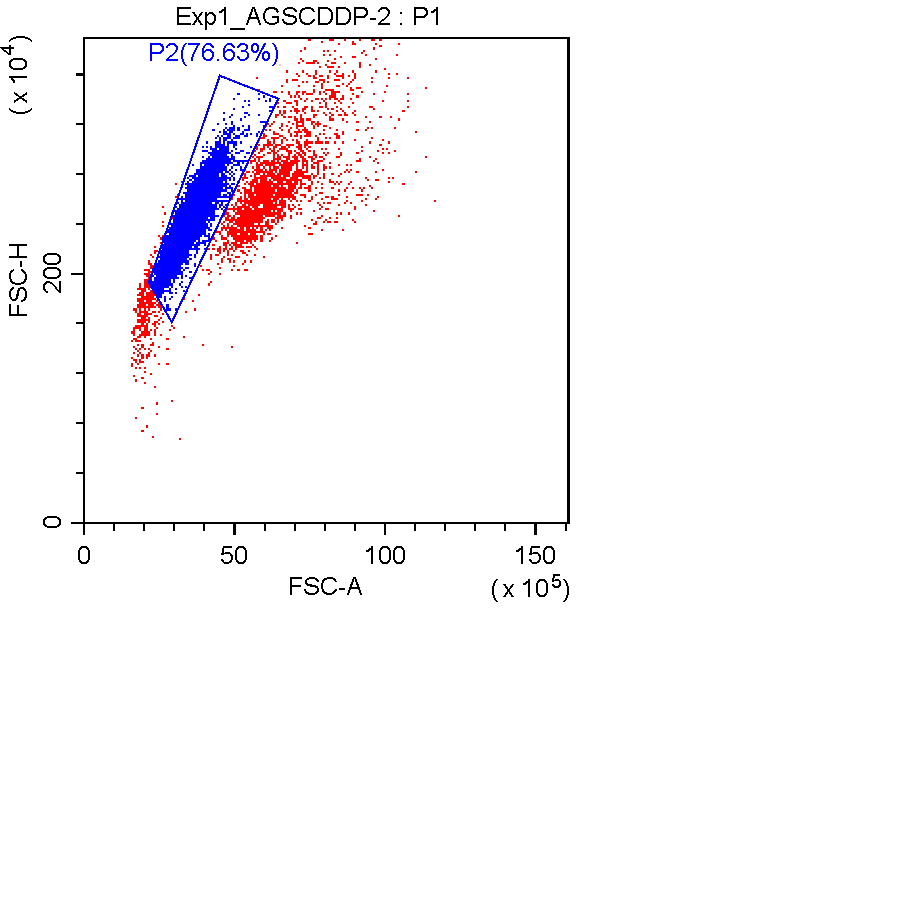

Supplement: Supplemental Information 1 [file peerj-11-15441-s001.zip › Raw data submitted/cell apoptosis/Fig. 7/AGSCDDP/Exp1_AGSCDDP-2_Plot2.bmp]

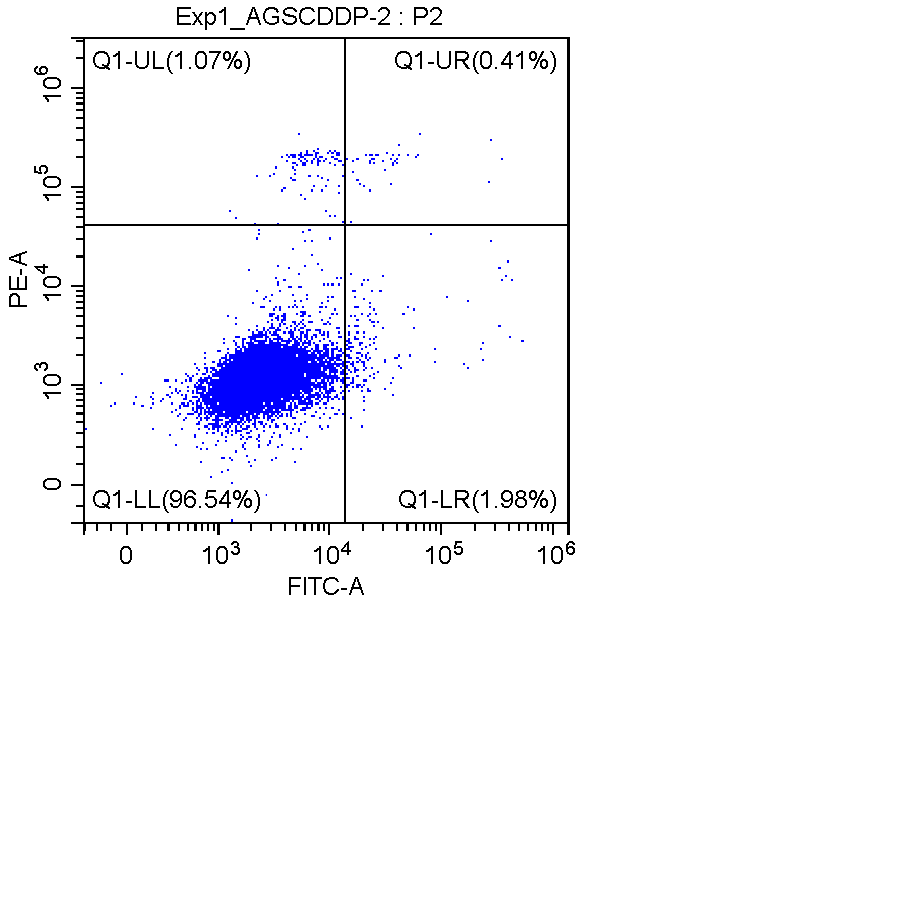

Supplement: Supplemental Information 1 [file peerj-11-15441-s001.zip › Raw data submitted/cell apoptosis/Fig. 7/AGSCDDP/Exp1_AGSCDDP-2_Plot3.bmp]

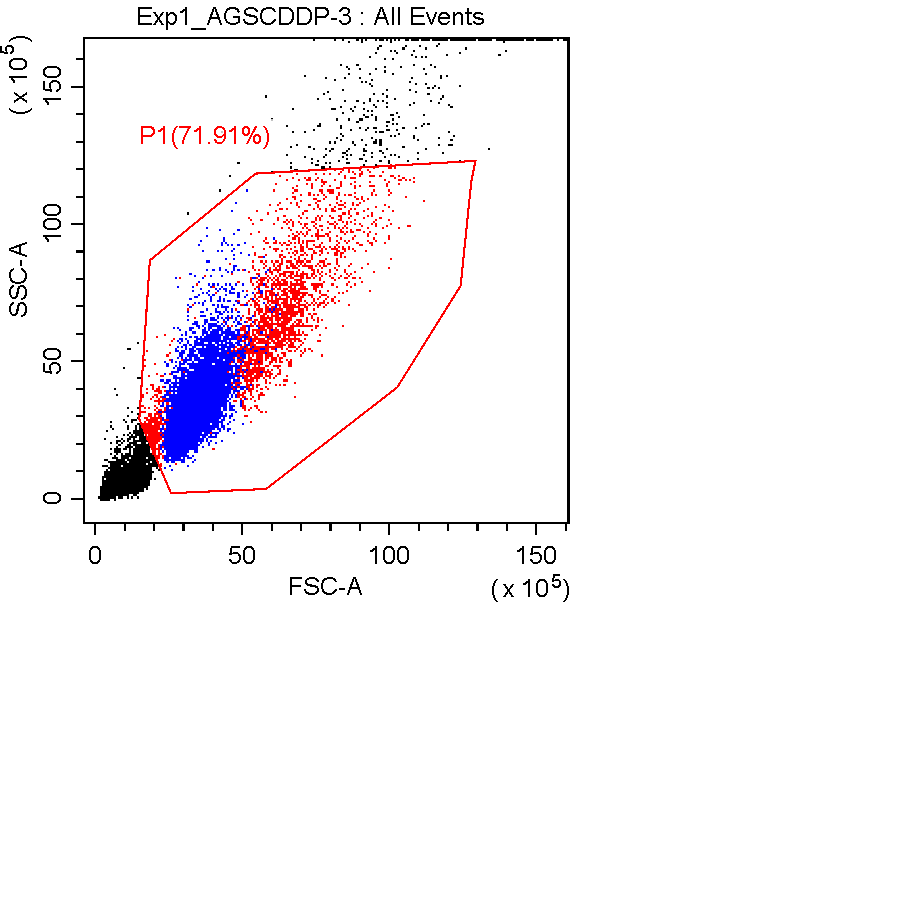

Supplement: Supplemental Information 1 [file peerj-11-15441-s001.zip › Raw data submitted/cell apoptosis/Fig. 7/AGSCDDP/Exp1_AGSCDDP-3_Plot1.bmp]

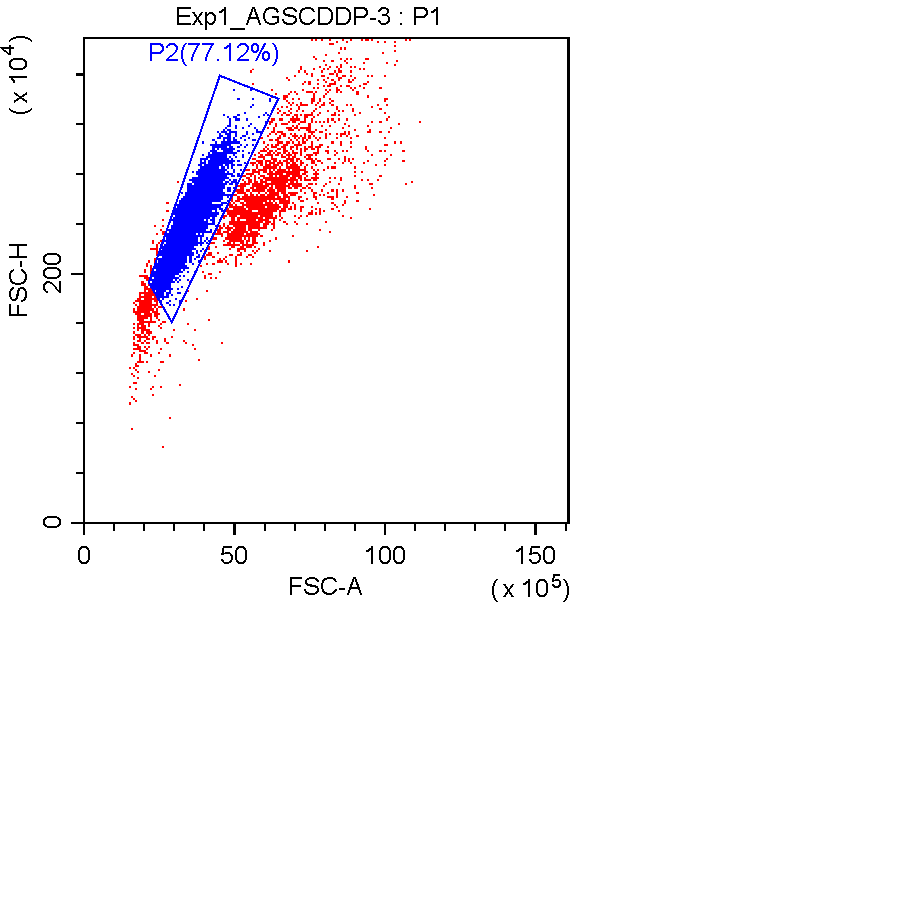

Supplement: Supplemental Information 1 [file peerj-11-15441-s001.zip › Raw data submitted/cell apoptosis/Fig. 7/AGSCDDP/Exp1_AGSCDDP-3_Plot2.bmp]

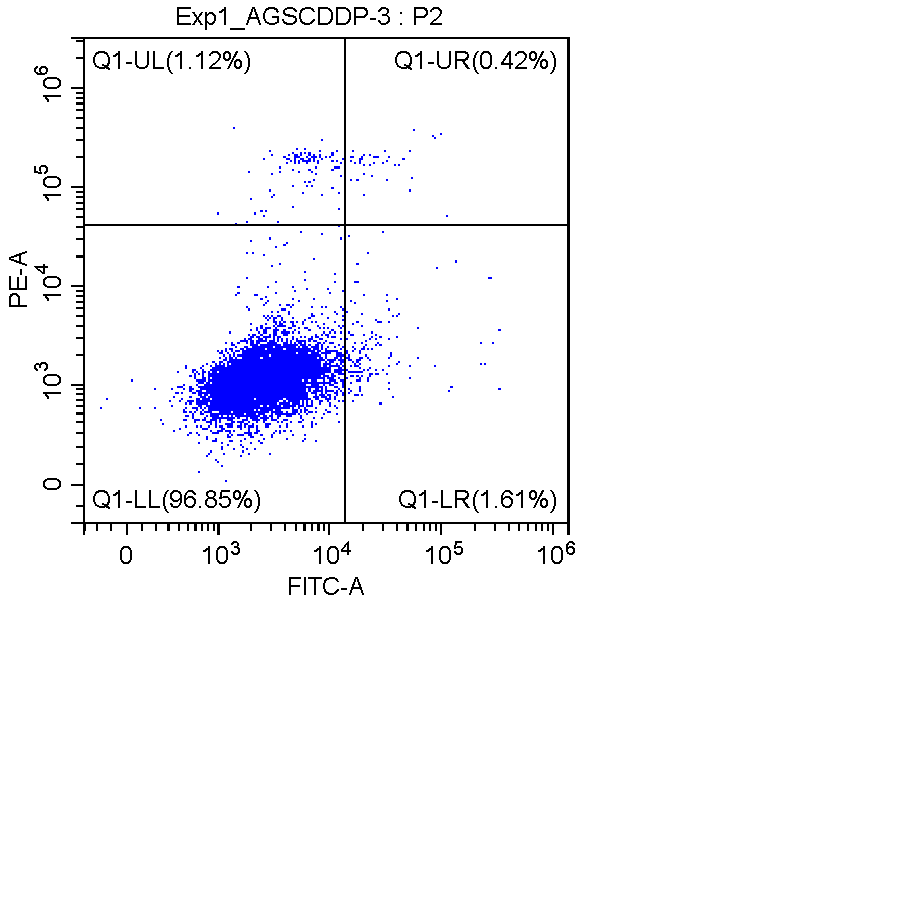

Supplement: Supplemental Information 1 [file peerj-11-15441-s001.zip › Raw data submitted/cell apoptosis/Fig. 7/AGSCDDP/Exp1_AGSCDDP-3_Plot3.bmp]

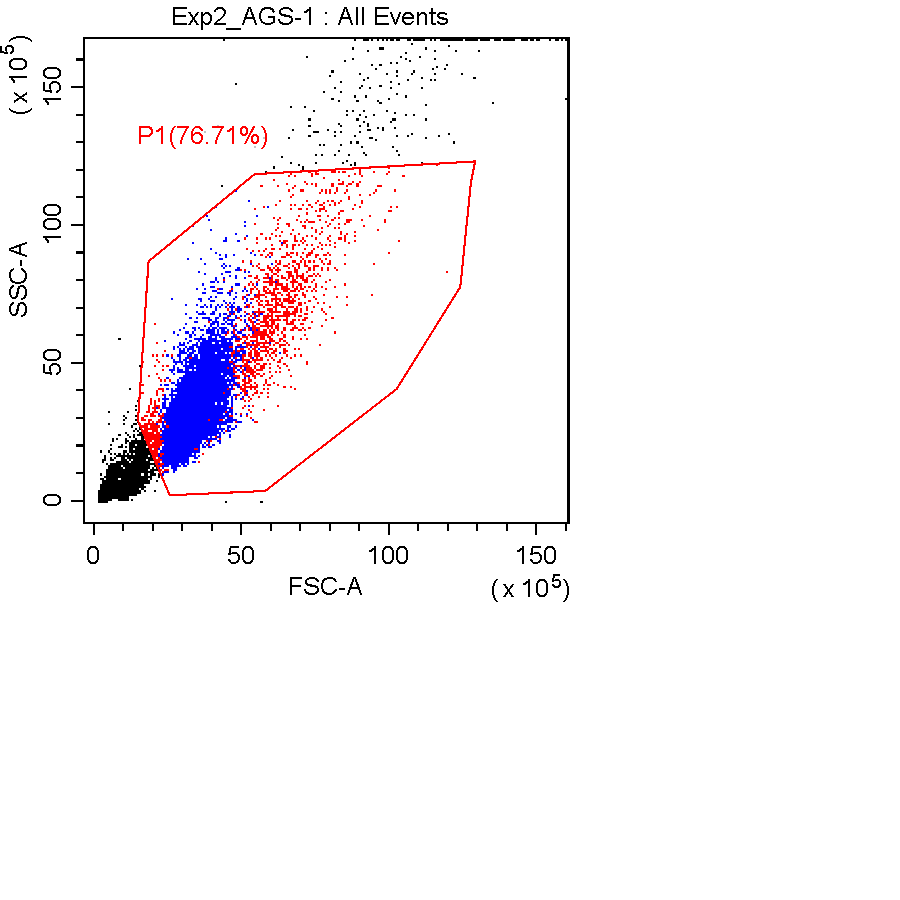

Supplement: Supplemental Information 1 [file peerj-11-15441-s001.zip › Raw data submitted/cell apoptosis/Fig. 8/AGS/Exp2_AGS-1_Plot1.bmp]

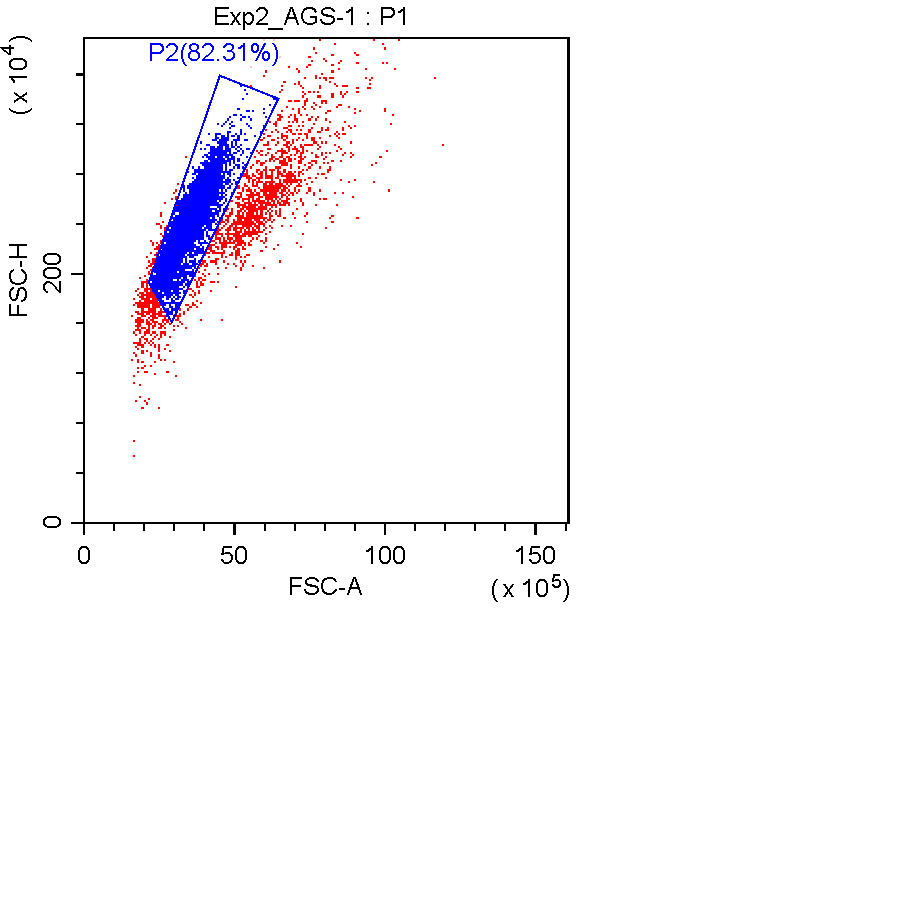

Supplement: Supplemental Information 1 [file peerj-11-15441-s001.zip › Raw data submitted/cell apoptosis/Fig. 8/AGS/Exp2_AGS-1_Plot2.bmp]

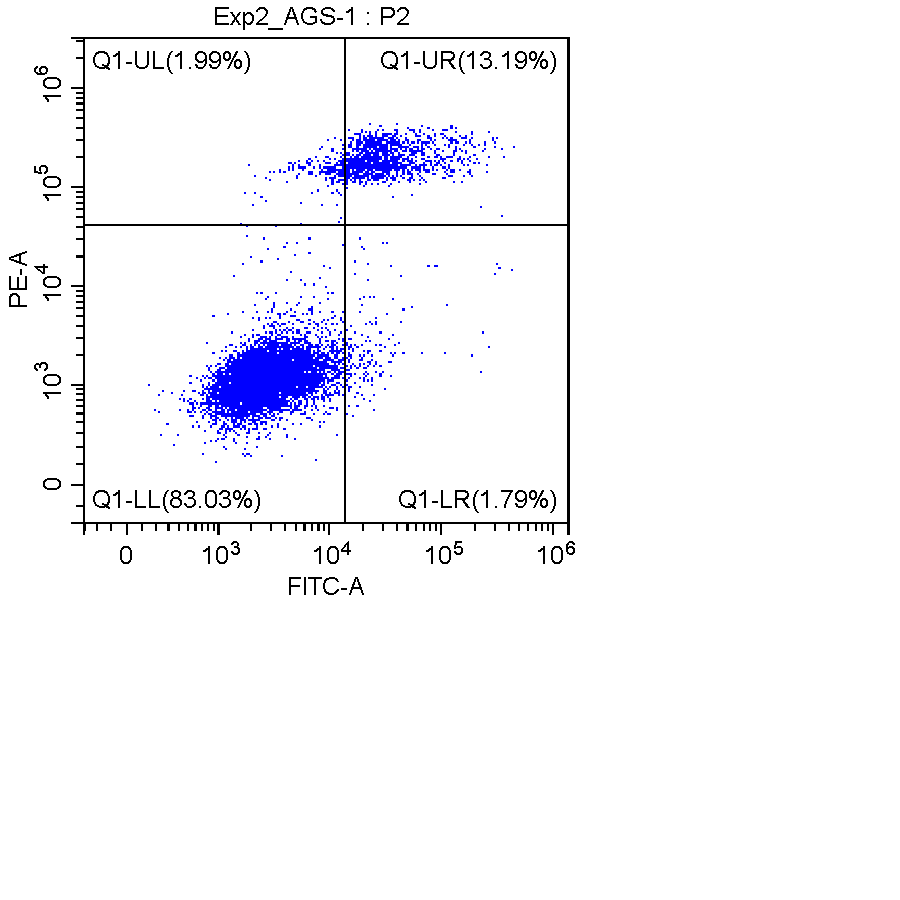

Supplement: Supplemental Information 1 [file peerj-11-15441-s001.zip › Raw data submitted/cell apoptosis/Fig. 8/AGS/Exp2_AGS-1_Plot3.bmp]

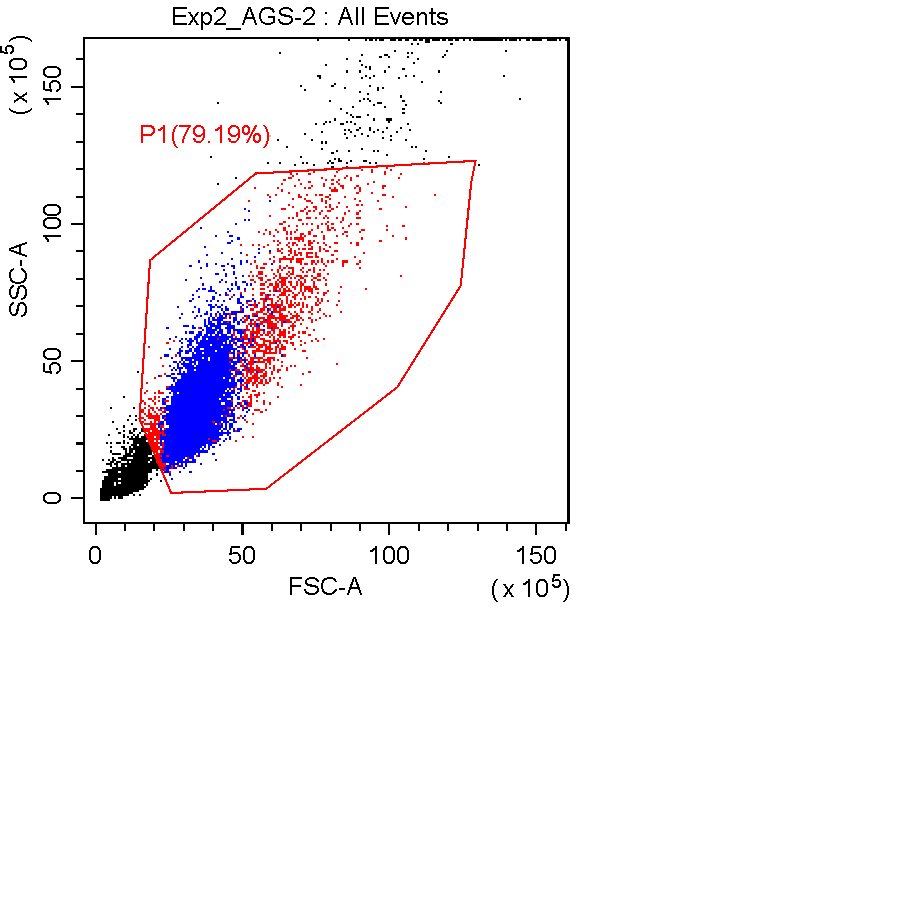

Supplement: Supplemental Information 1 [file peerj-11-15441-s001.zip › Raw data submitted/cell apoptosis/Fig. 8/AGS/Exp2_AGS-2_Plot1.bmp]

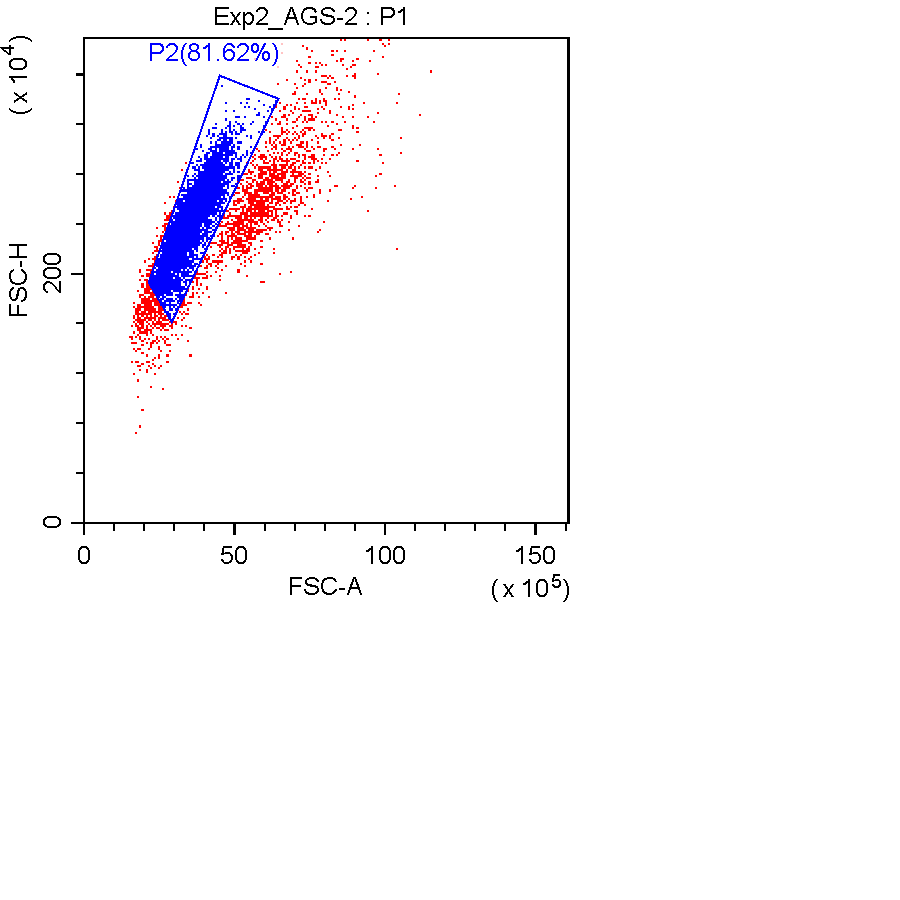

Supplement: Supplemental Information 1 [file peerj-11-15441-s001.zip › Raw data submitted/cell apoptosis/Fig. 8/AGS/Exp2_AGS-2_Plot2.bmp]

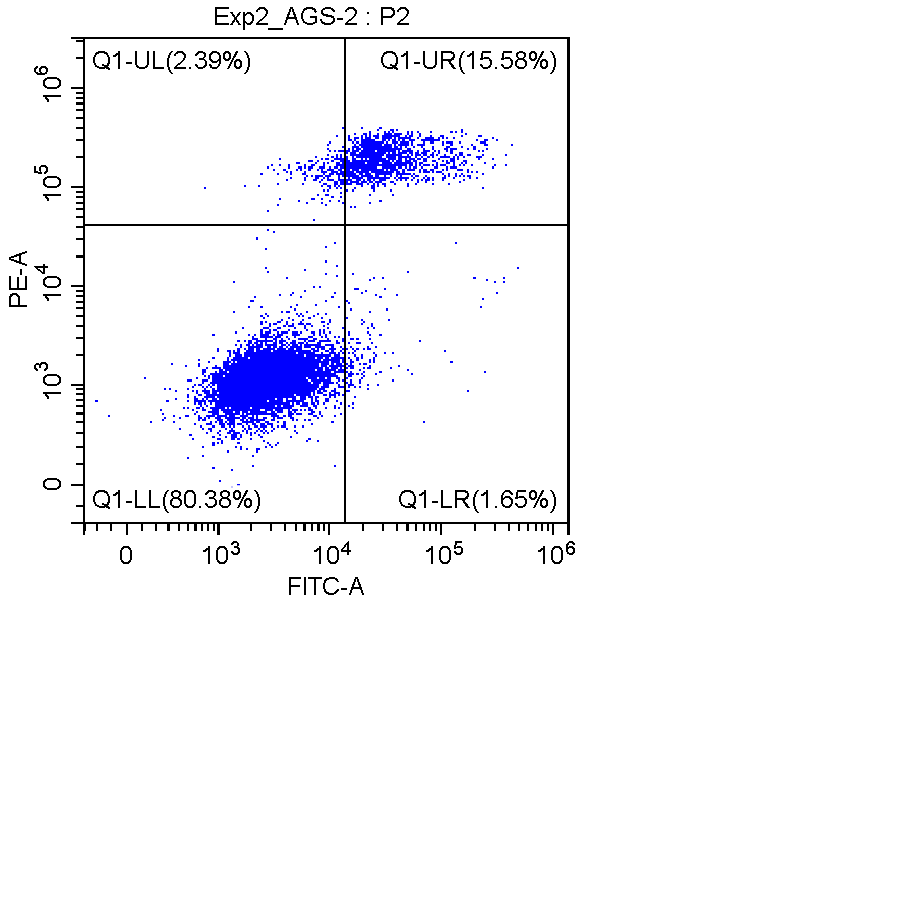

Supplement: Supplemental Information 1 [file peerj-11-15441-s001.zip › Raw data submitted/cell apoptosis/Fig. 8/AGS/Exp2_AGS-2_Plot3.bmp]

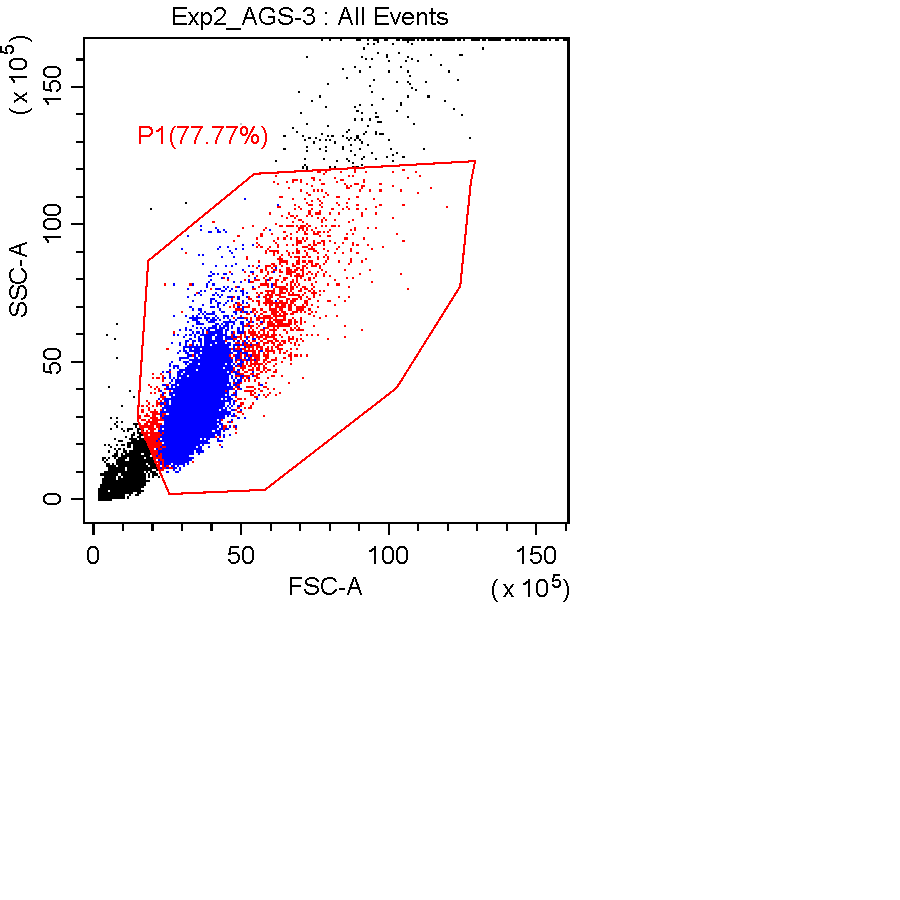

Supplement: Supplemental Information 1 [file peerj-11-15441-s001.zip › Raw data submitted/cell apoptosis/Fig. 8/AGS/Exp2_AGS-3_Plot1.bmp]

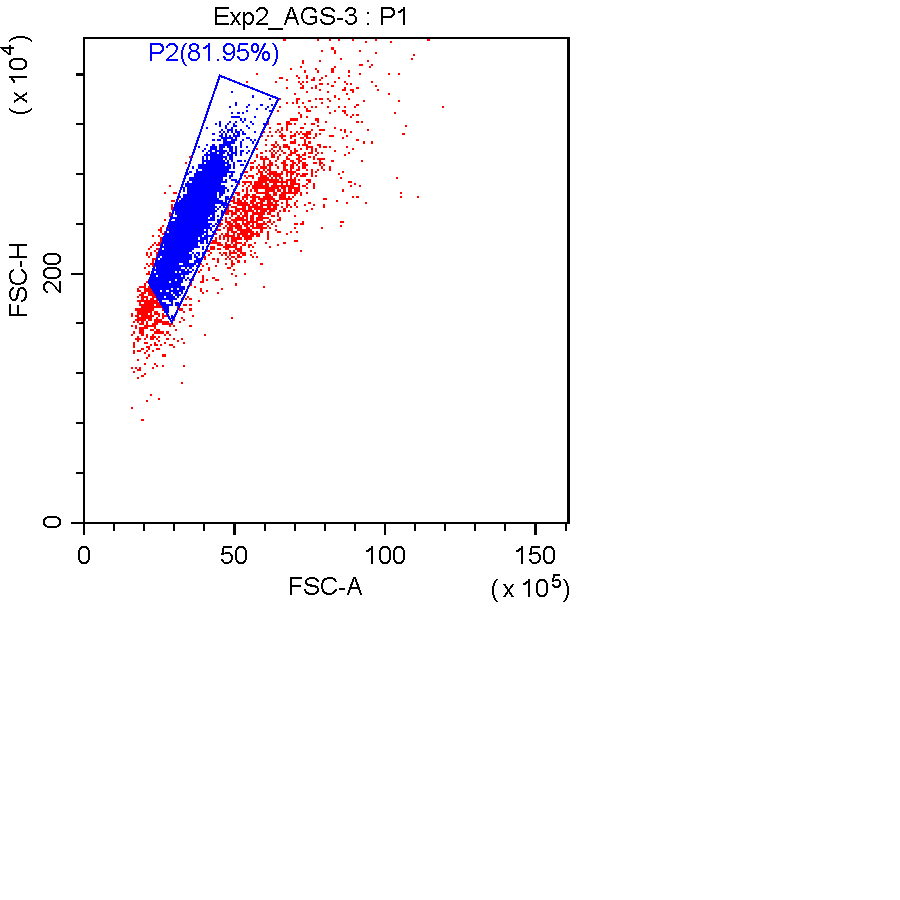

Supplement: Supplemental Information 1 [file peerj-11-15441-s001.zip › Raw data submitted/cell apoptosis/Fig. 8/AGS/Exp2_AGS-3_Plot2.bmp]

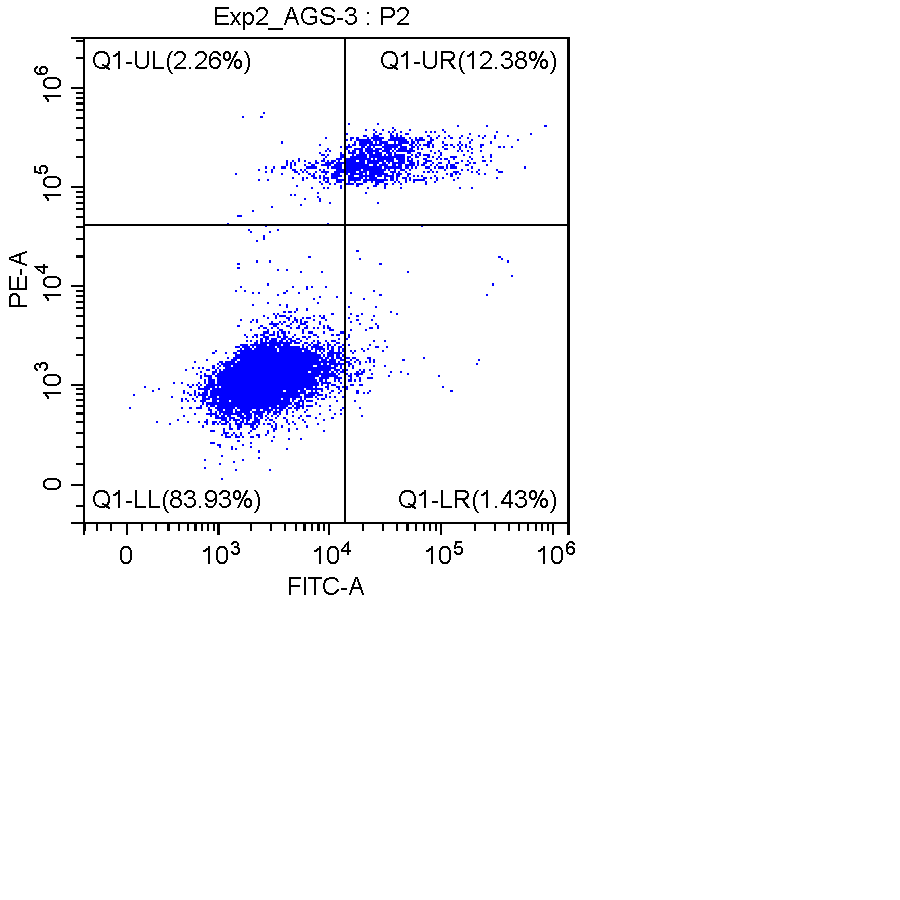

Supplement: Supplemental Information 1 [file peerj-11-15441-s001.zip › Raw data submitted/cell apoptosis/Fig. 8/AGS/Exp2_AGS-3_Plot3.bmp]

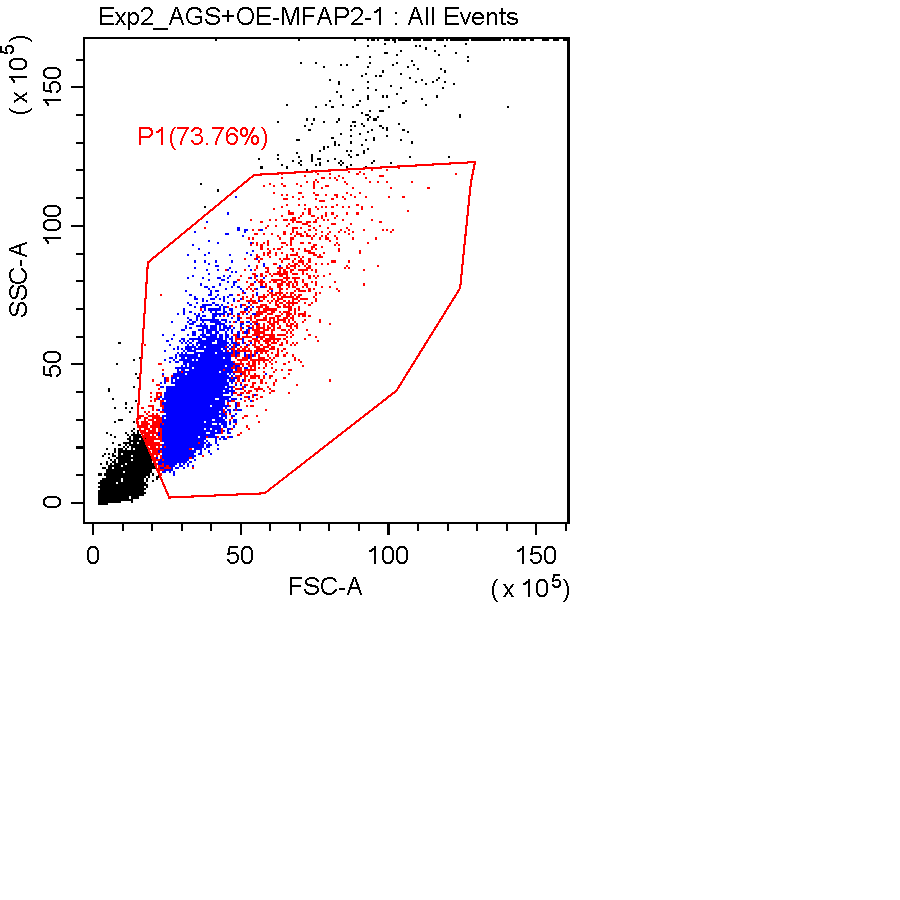

Supplement: Supplemental Information 1 [file peerj-11-15441-s001.zip › Raw data submitted/cell apoptosis/Fig. 8/AGS+OE-MFAP2/Exp2_AGS+OE-MFAP2-1_Plot1.bmp]

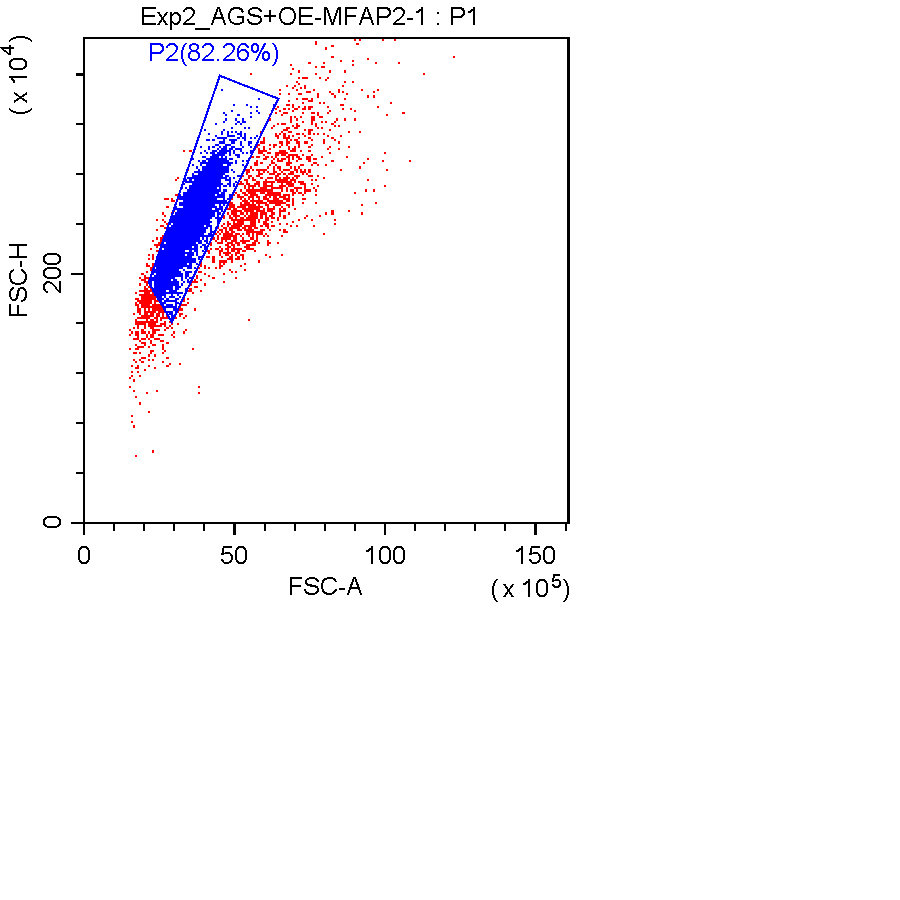

Supplement: Supplemental Information 1 [file peerj-11-15441-s001.zip › Raw data submitted/cell apoptosis/Fig. 8/AGS+OE-MFAP2/Exp2_AGS+OE-MFAP2-1_Plot2.bmp]

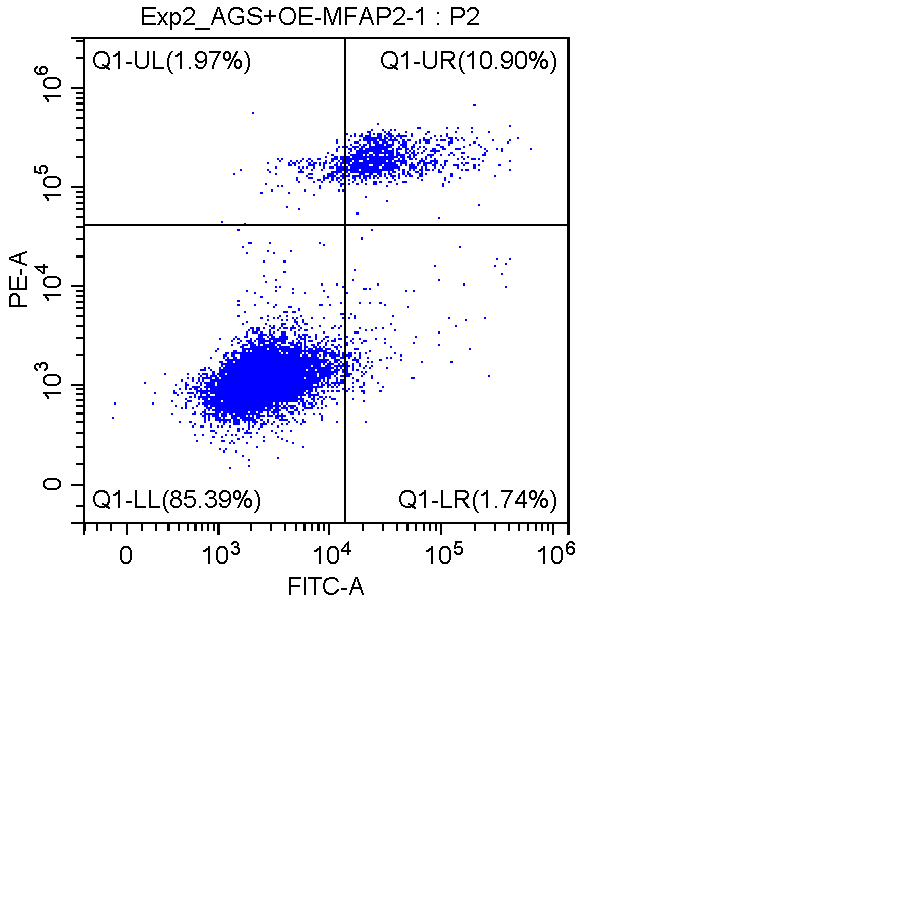

Supplement: Supplemental Information 1 [file peerj-11-15441-s001.zip › Raw data submitted/cell apoptosis/Fig. 8/AGS+OE-MFAP2/Exp2_AGS+OE-MFAP2-1_Plot3.bmp]

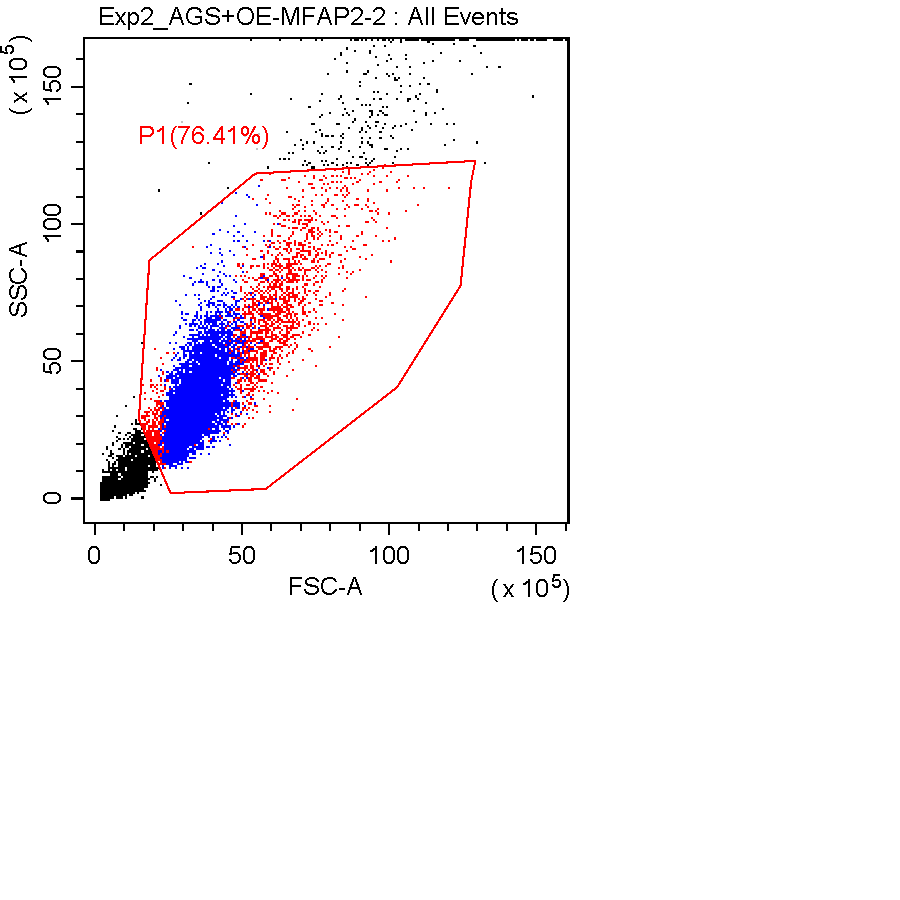

Supplement: Supplemental Information 1 [file peerj-11-15441-s001.zip › Raw data submitted/cell apoptosis/Fig. 8/AGS+OE-MFAP2/Exp2_AGS+OE-MFAP2-2_Plot1.bmp]

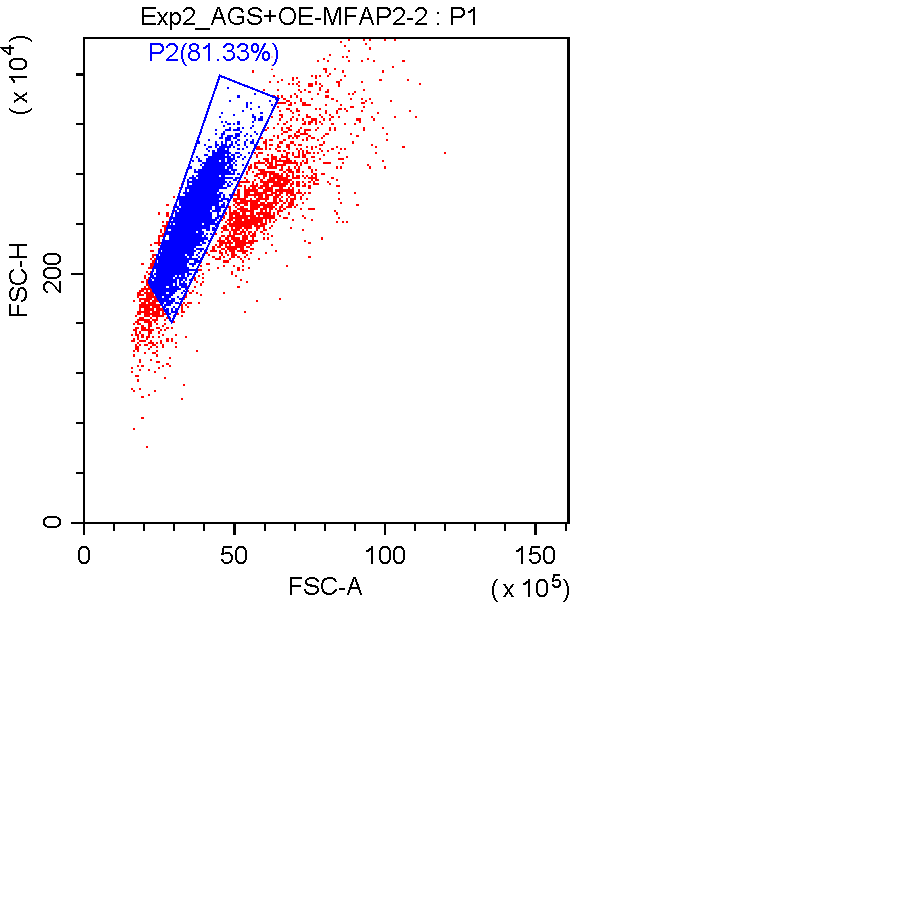

Supplement: Supplemental Information 1 [file peerj-11-15441-s001.zip › Raw data submitted/cell apoptosis/Fig. 8/AGS+OE-MFAP2/Exp2_AGS+OE-MFAP2-2_Plot2.bmp]

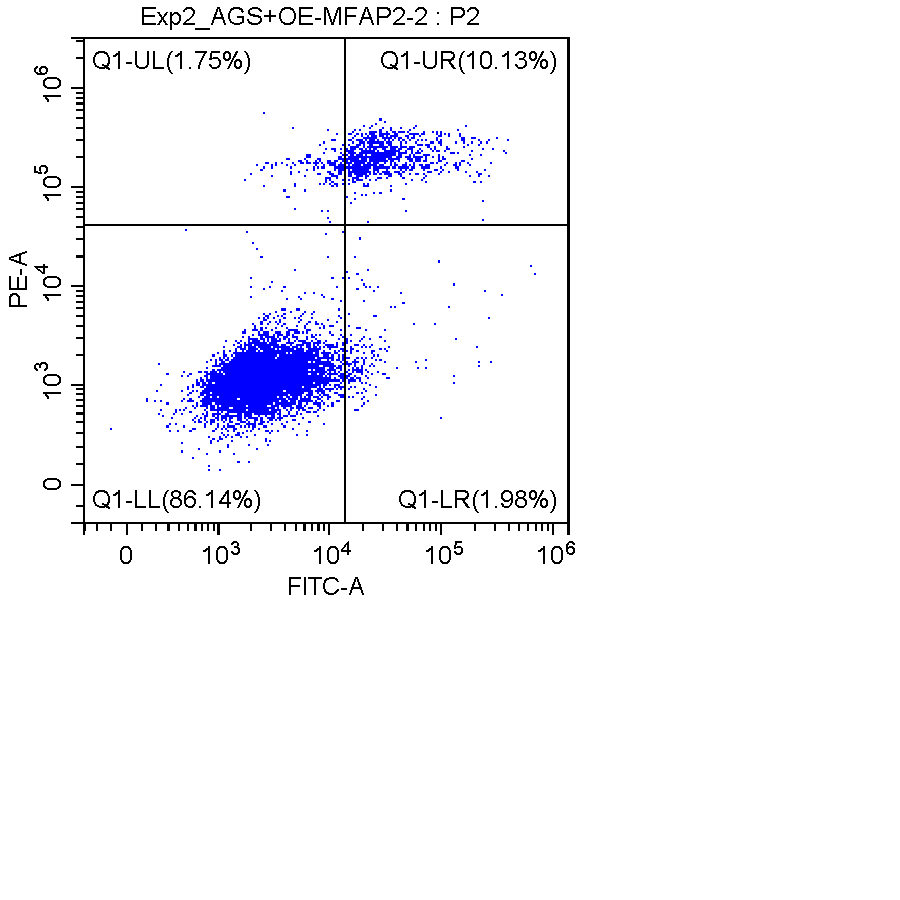

Supplement: Supplemental Information 1 [file peerj-11-15441-s001.zip › Raw data submitted/cell apoptosis/Fig. 8/AGS+OE-MFAP2/Exp2_AGS+OE-MFAP2-2_Plot3.bmp]

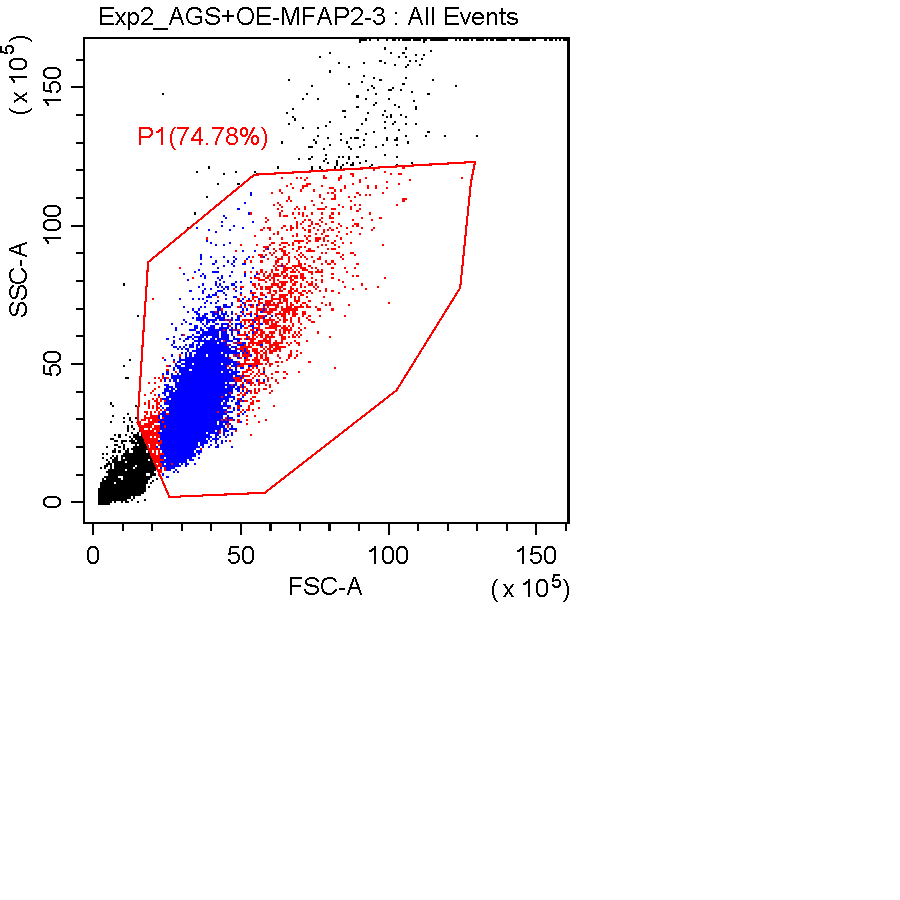

Supplement: Supplemental Information 1 [file peerj-11-15441-s001.zip › Raw data submitted/cell apoptosis/Fig. 8/AGS+OE-MFAP2/Exp2_AGS+OE-MFAP2-3_Plot1.bmp]

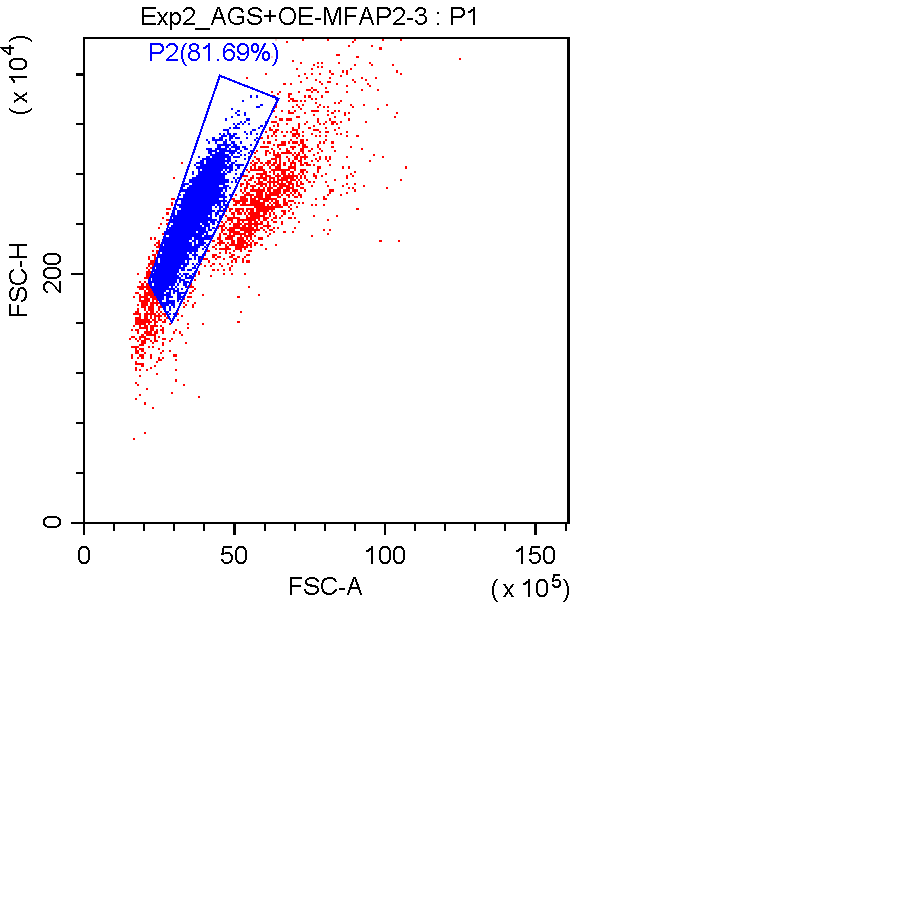

Supplement: Supplemental Information 1 [file peerj-11-15441-s001.zip › Raw data submitted/cell apoptosis/Fig. 8/AGS+OE-MFAP2/Exp2_AGS+OE-MFAP2-3_Plot2.bmp]

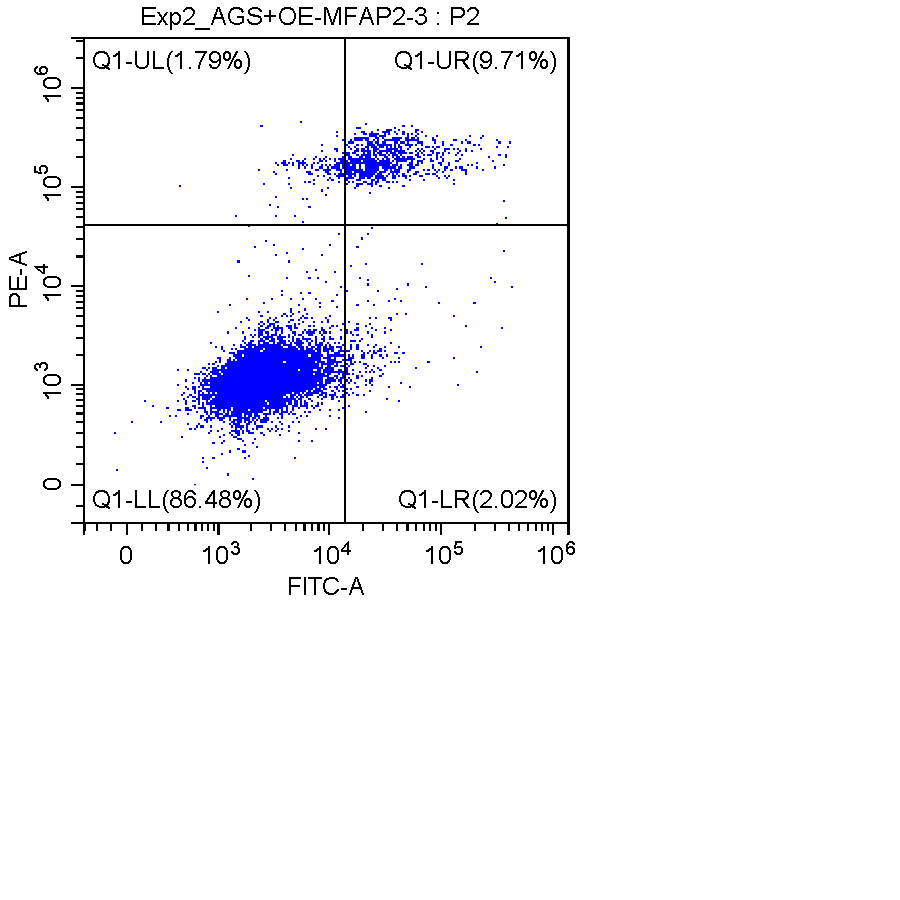

Supplement: Supplemental Information 1 [file peerj-11-15441-s001.zip › Raw data submitted/cell apoptosis/Fig. 8/AGS+OE-MFAP2/Exp2_AGS+OE-MFAP2-3_Plot3.bmp]

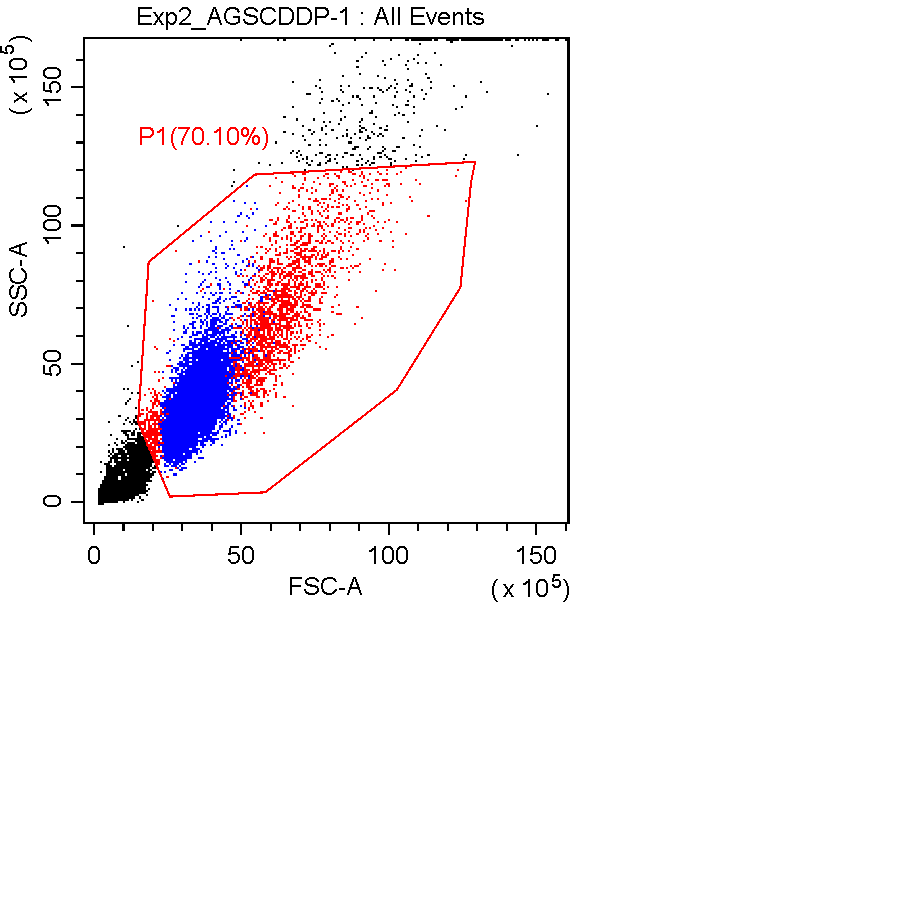

Supplement: Supplemental Information 1 [file peerj-11-15441-s001.zip › Raw data submitted/cell apoptosis/Fig. 8/AGSCDDP/Exp2_AGSCDDP-1_Plot1.bmp]

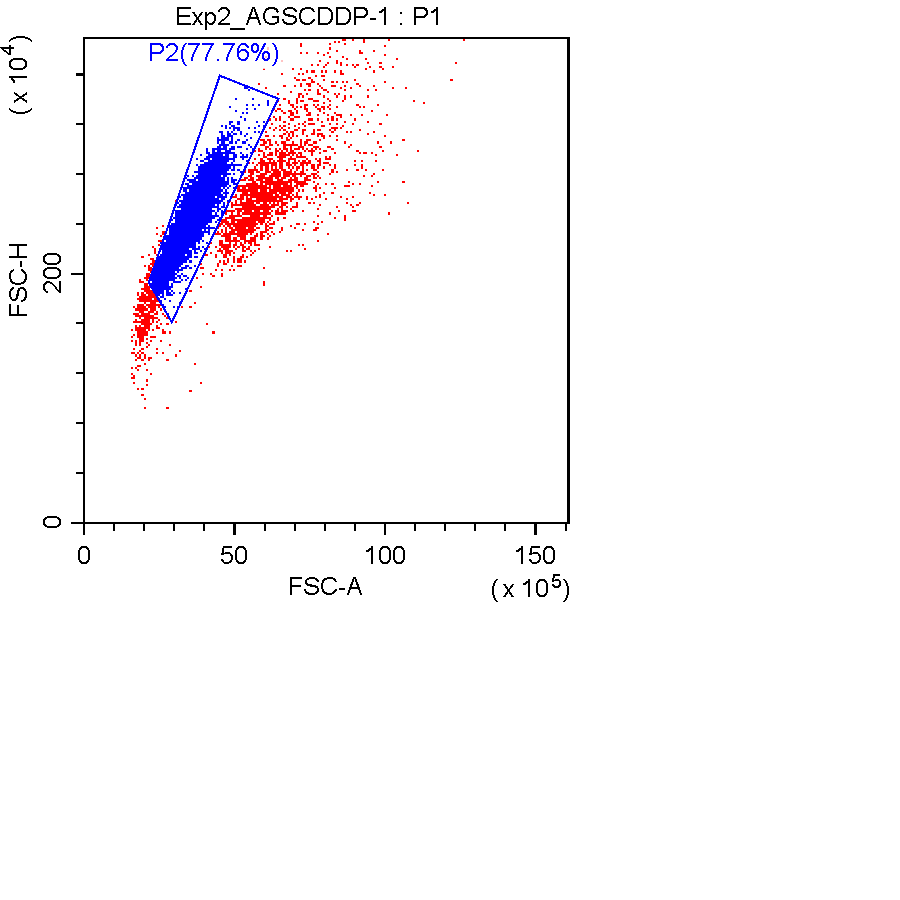

Supplement: Supplemental Information 1 [file peerj-11-15441-s001.zip › Raw data submitted/cell apoptosis/Fig. 8/AGSCDDP/Exp2_AGSCDDP-1_Plot2.bmp]

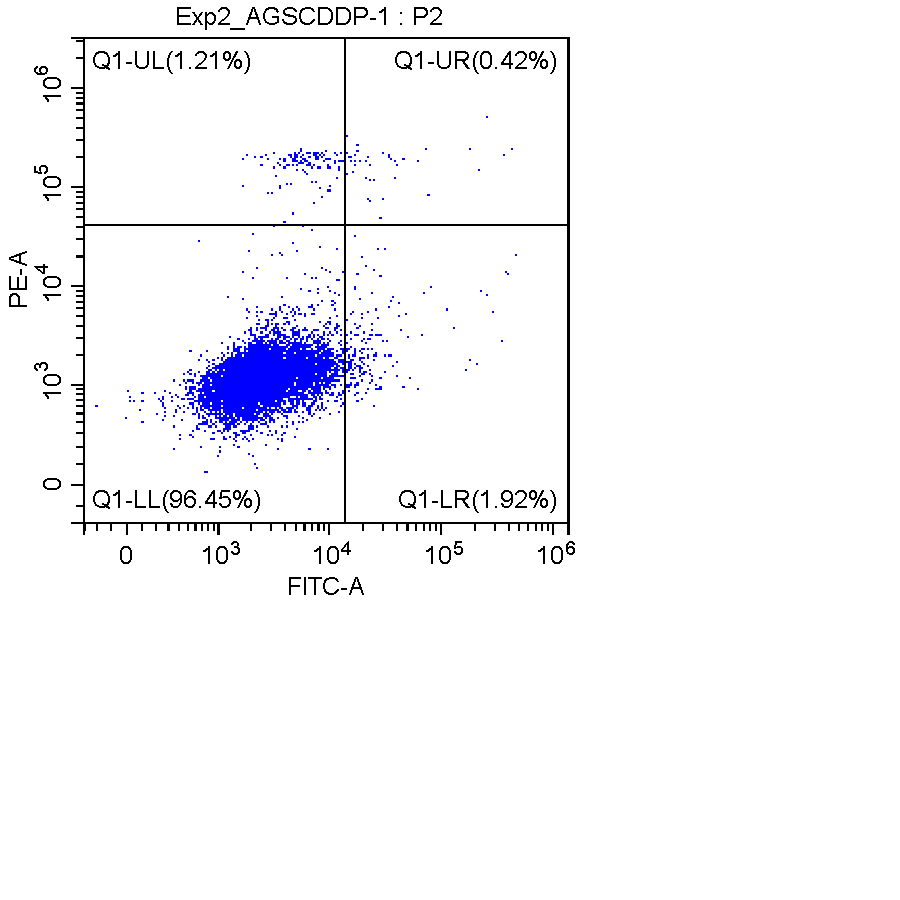

Supplement: Supplemental Information 1 [file peerj-11-15441-s001.zip › Raw data submitted/cell apoptosis/Fig. 8/AGSCDDP/Exp2_AGSCDDP-1_Plot3.bmp]

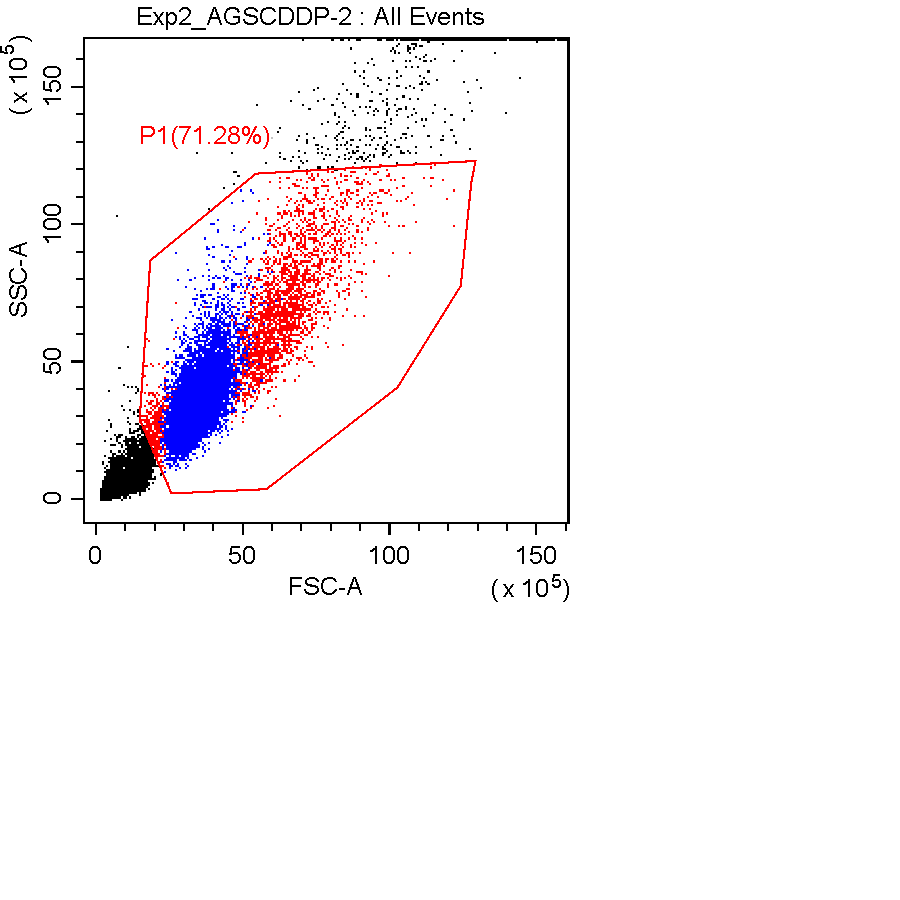

Supplement: Supplemental Information 1 [file peerj-11-15441-s001.zip › Raw data submitted/cell apoptosis/Fig. 8/AGSCDDP/Exp2_AGSCDDP-2_Plot1.bmp]

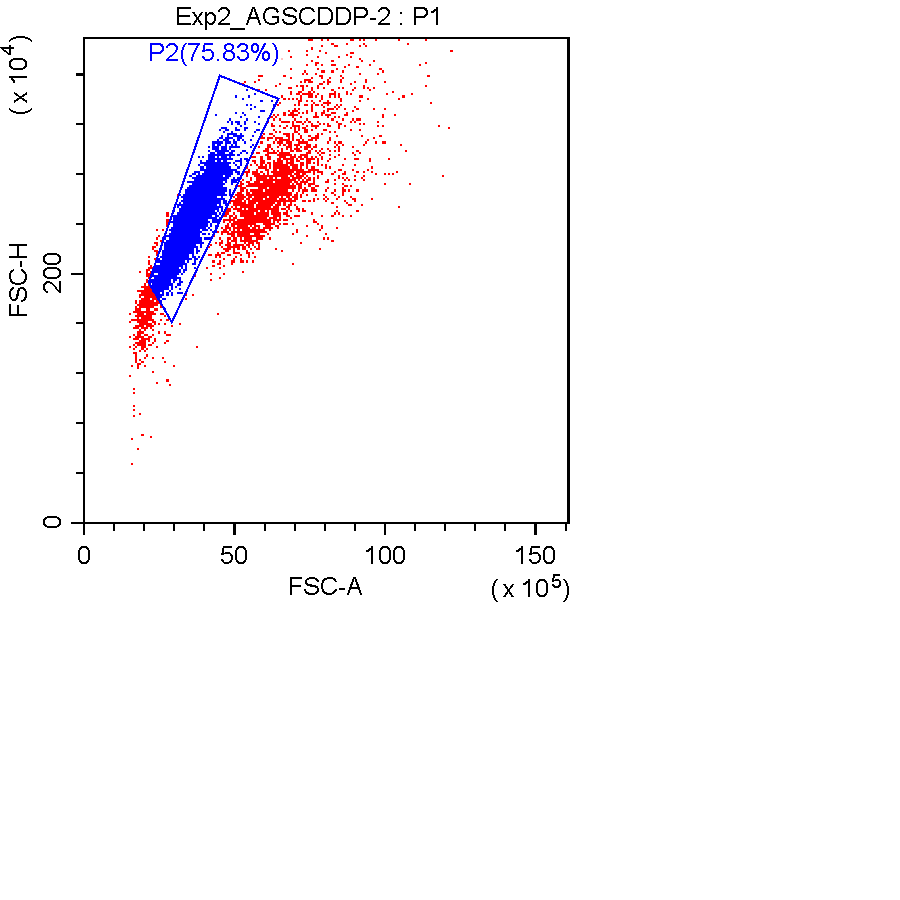

Supplement: Supplemental Information 1 [file peerj-11-15441-s001.zip › Raw data submitted/cell apoptosis/Fig. 8/AGSCDDP/Exp2_AGSCDDP-2_Plot2.bmp]

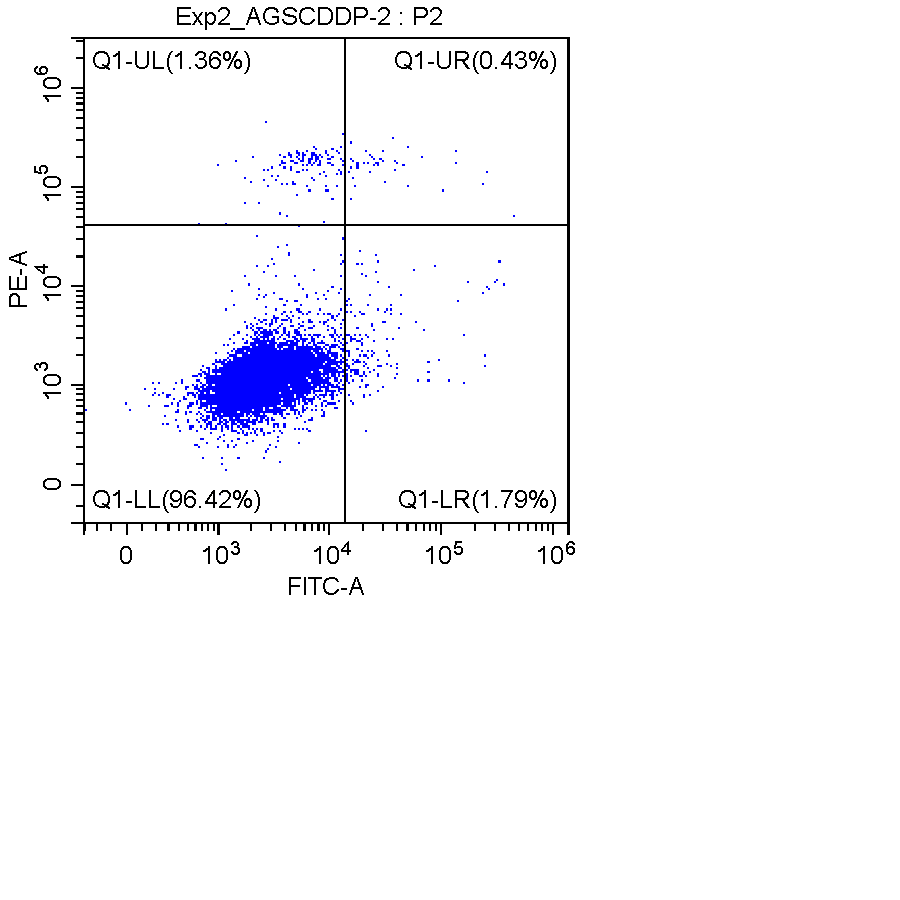

Supplement: Supplemental Information 1 [file peerj-11-15441-s001.zip › Raw data submitted/cell apoptosis/Fig. 8/AGSCDDP/Exp2_AGSCDDP-2_Plot3.bmp]

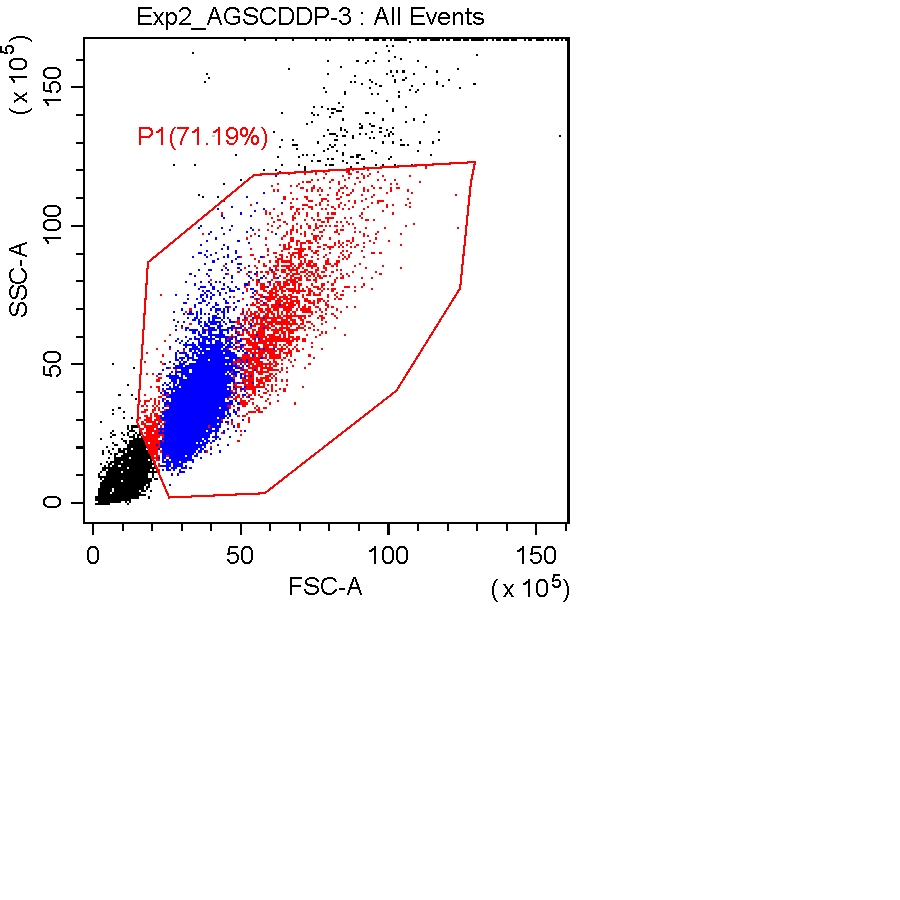

Supplement: Supplemental Information 1 [file peerj-11-15441-s001.zip › Raw data submitted/cell apoptosis/Fig. 8/AGSCDDP/Exp2_AGSCDDP-3_Plot1.bmp]

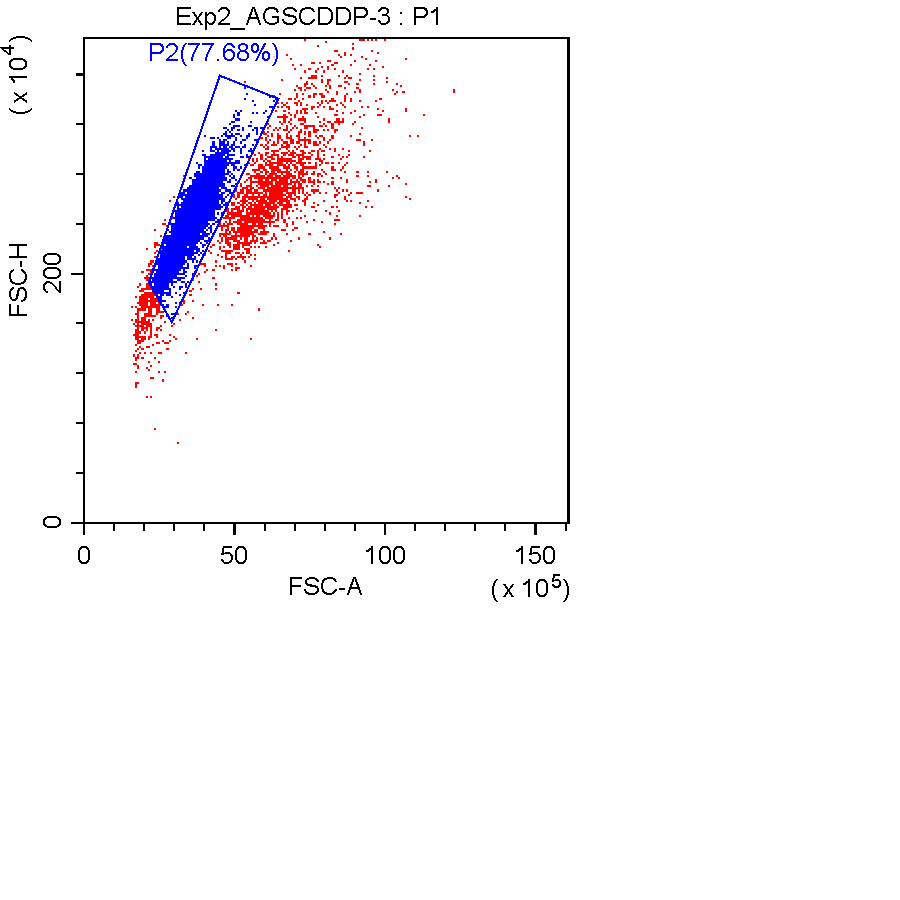

Supplement: Supplemental Information 1 [file peerj-11-15441-s001.zip › Raw data submitted/cell apoptosis/Fig. 8/AGSCDDP/Exp2_AGSCDDP-3_Plot2.bmp]

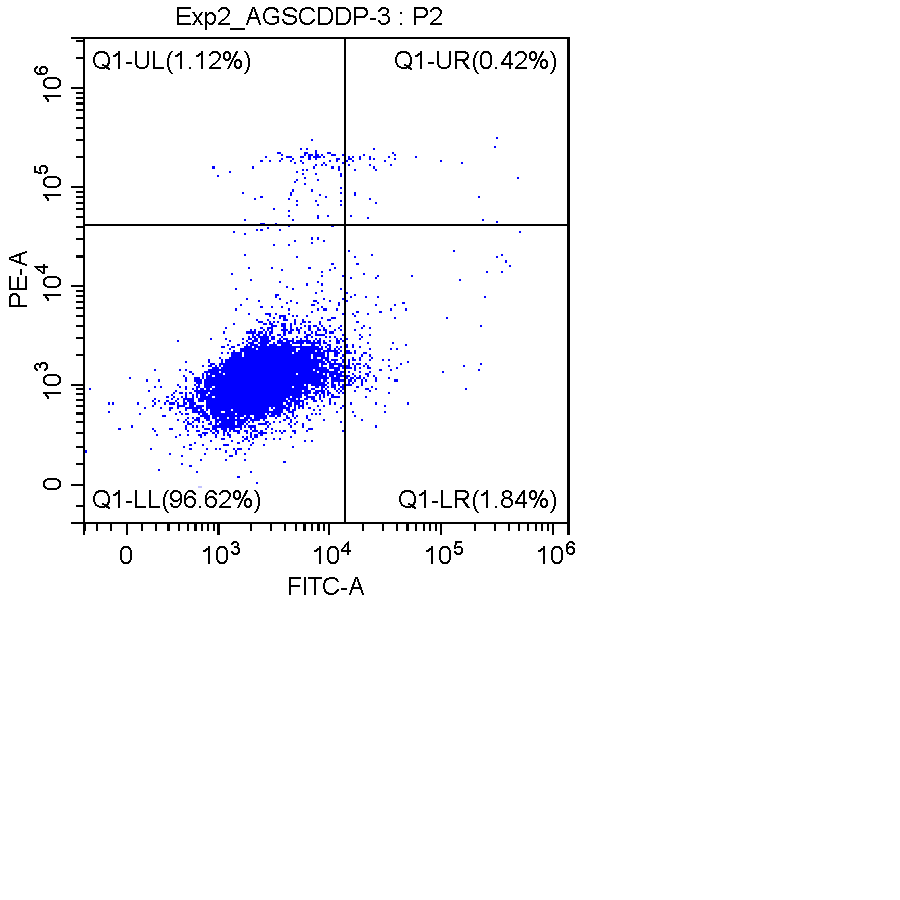

Supplement: Supplemental Information 1 [file peerj-11-15441-s001.zip › Raw data submitted/cell apoptosis/Fig. 8/AGSCDDP/Exp2_AGSCDDP-3_Plot3.bmp]

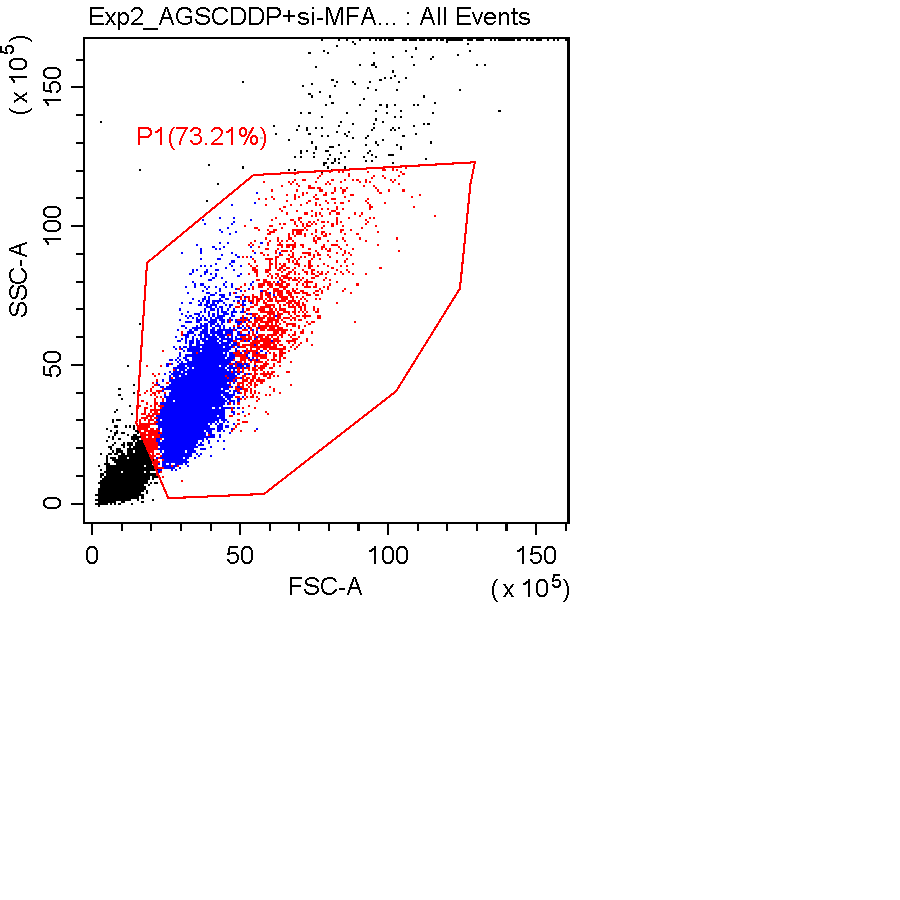

Supplement: Supplemental Information 1 [file peerj-11-15441-s001.zip › Raw data submitted/cell apoptosis/Fig. 8/AGSCDDP+si-MFAP2/Exp2_AGSCDDP+si-MFAP2-1_Plot1.bmp]

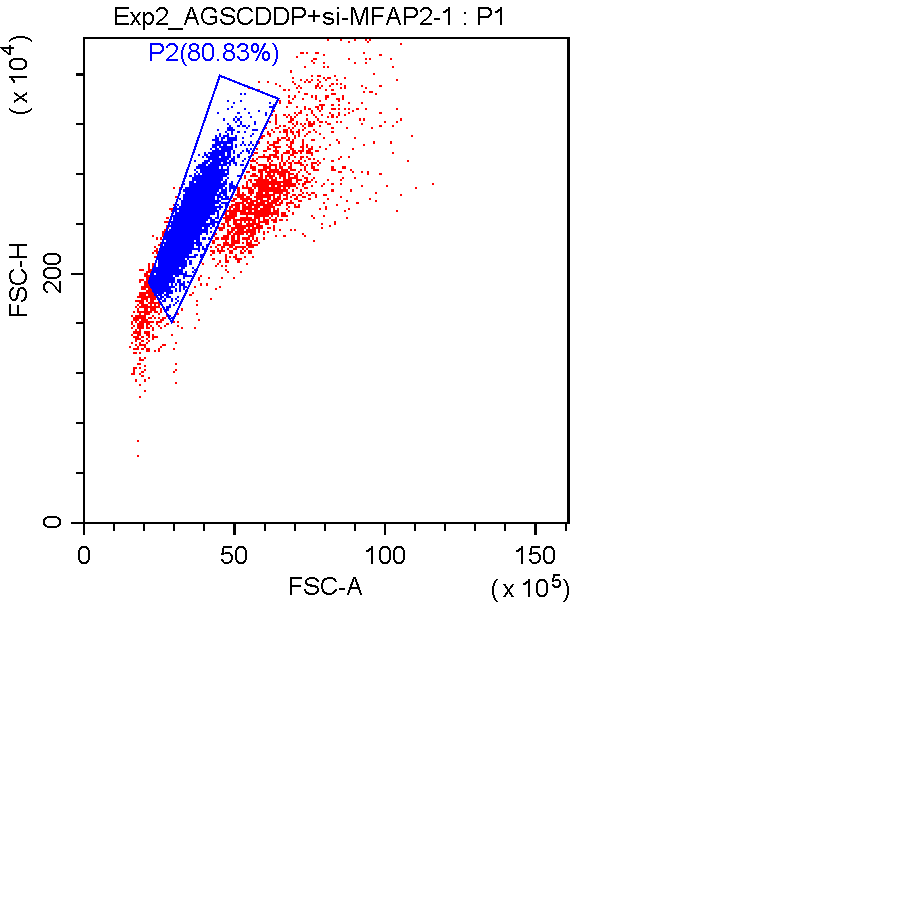

Supplement: Supplemental Information 1 [file peerj-11-15441-s001.zip › Raw data submitted/cell apoptosis/Fig. 8/AGSCDDP+si-MFAP2/Exp2_AGSCDDP+si-MFAP2-1_Plot2.bmp]

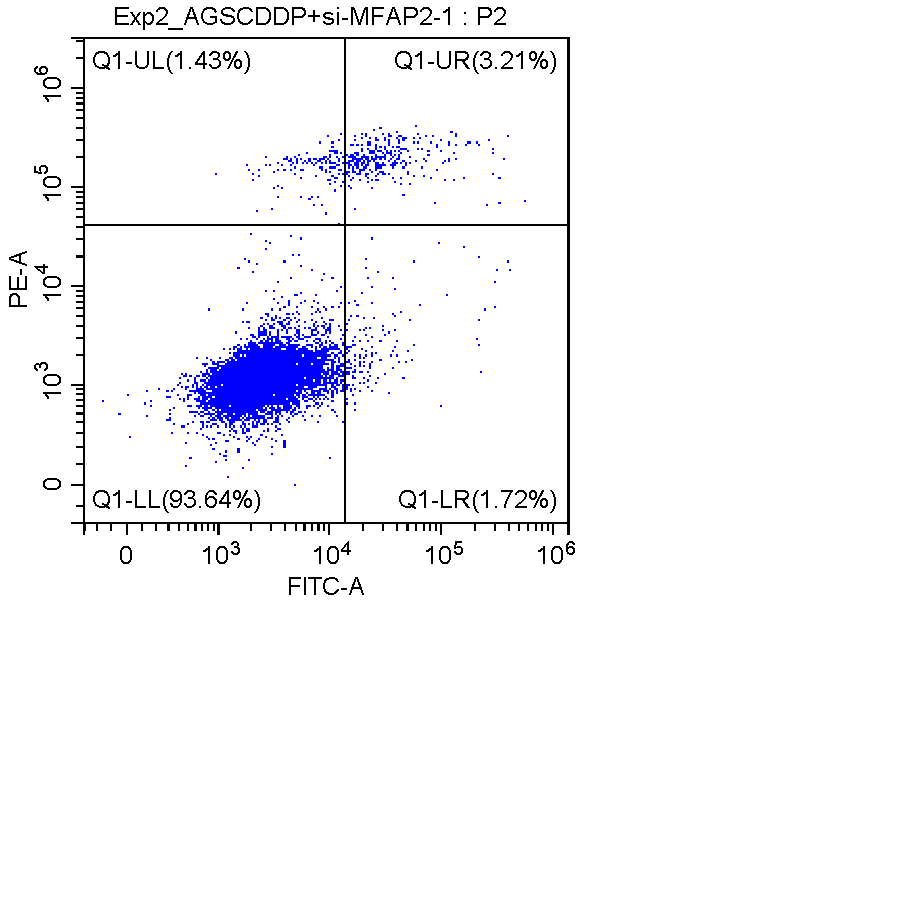

Supplement: Supplemental Information 1 [file peerj-11-15441-s001.zip › Raw data submitted/cell apoptosis/Fig. 8/AGSCDDP+si-MFAP2/Exp2_AGSCDDP+si-MFAP2-1_Plot3.bmp]

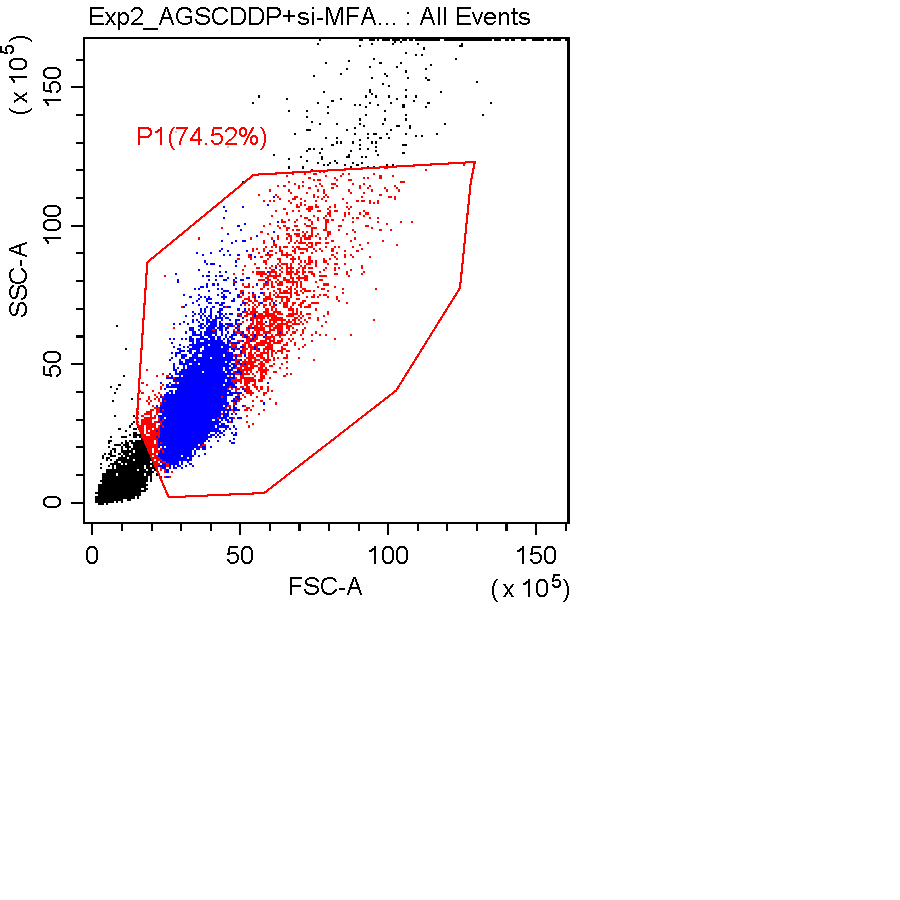

Supplement: Supplemental Information 1 [file peerj-11-15441-s001.zip › Raw data submitted/cell apoptosis/Fig. 8/AGSCDDP+si-MFAP2/Exp2_AGSCDDP+si-MFAP2-2_Plot1.bmp]

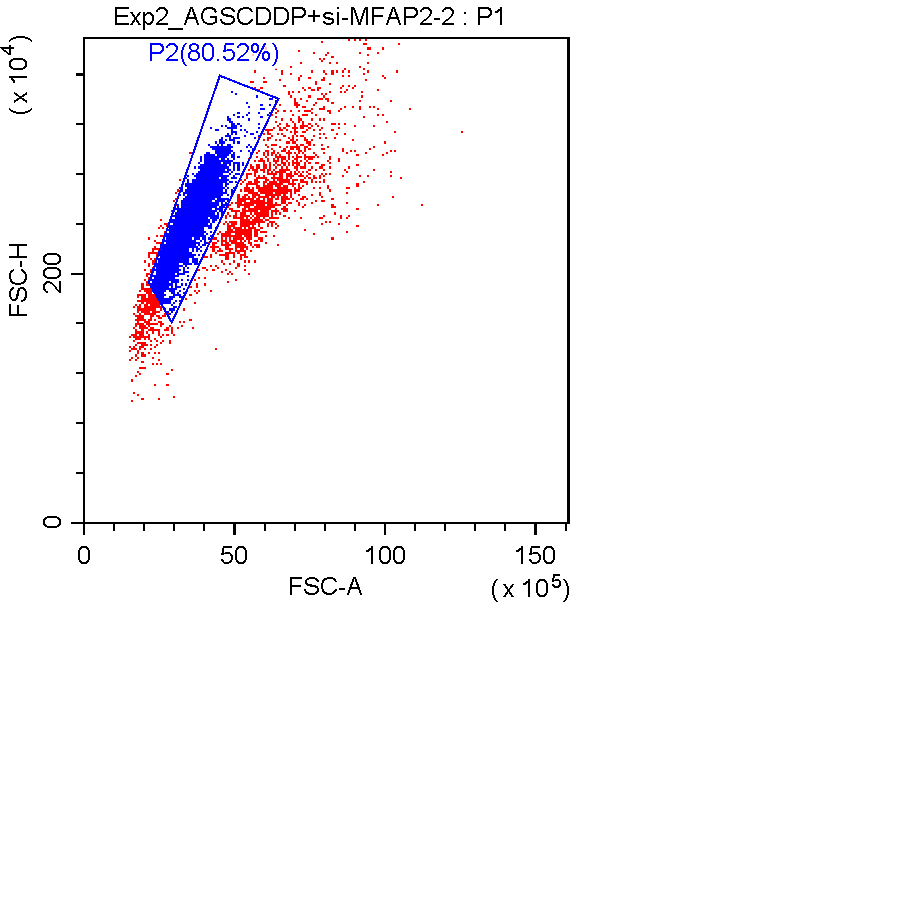

Supplement: Supplemental Information 1 [file peerj-11-15441-s001.zip › Raw data submitted/cell apoptosis/Fig. 8/AGSCDDP+si-MFAP2/Exp2_AGSCDDP+si-MFAP2-2_Plot2.bmp]

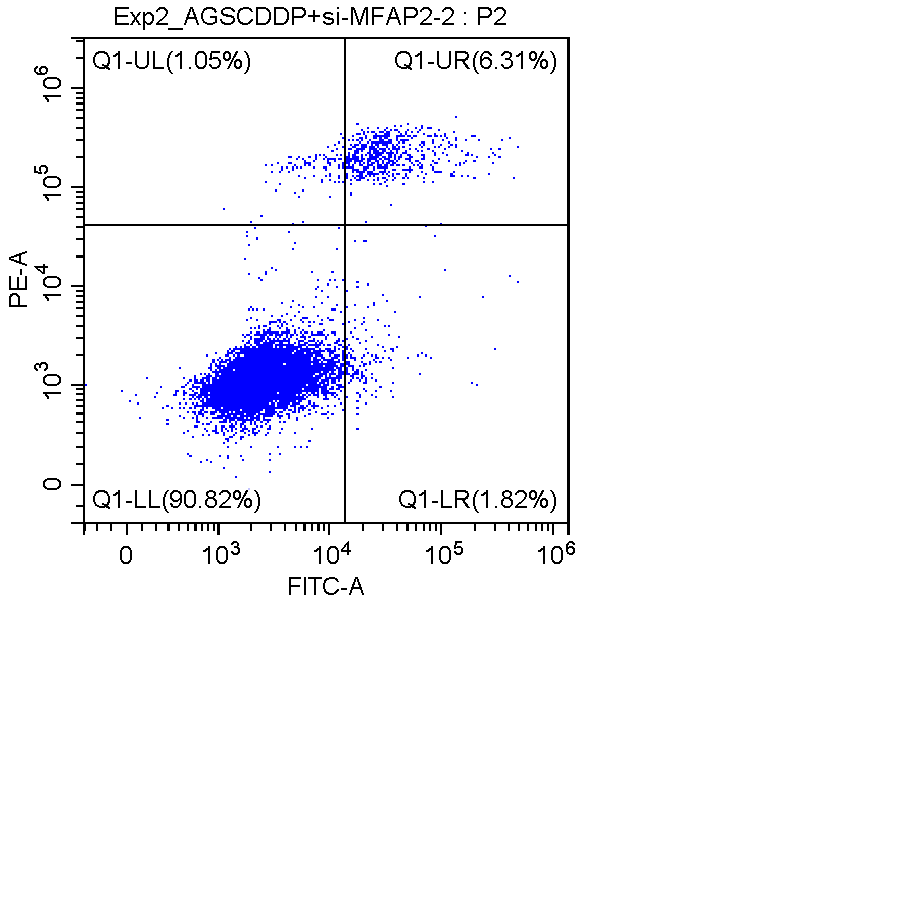

Supplement: Supplemental Information 1 [file peerj-11-15441-s001.zip › Raw data submitted/cell apoptosis/Fig. 8/AGSCDDP+si-MFAP2/Exp2_AGSCDDP+si-MFAP2-2_Plot3.bmp]

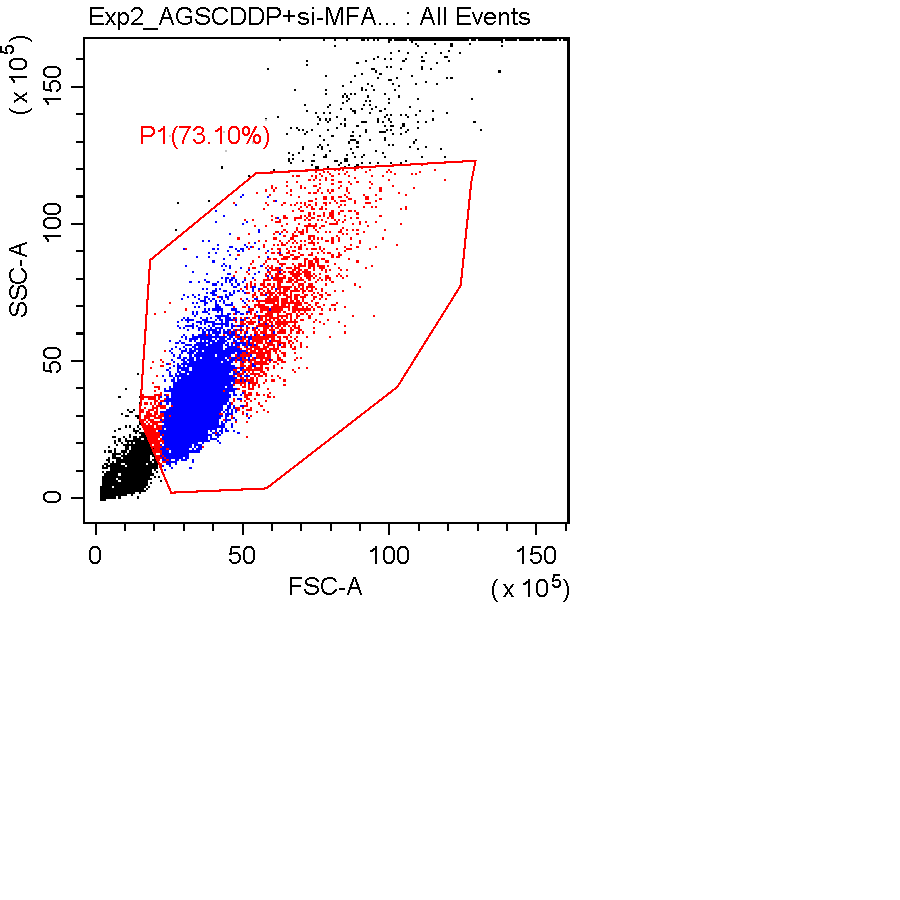

Supplement: Supplemental Information 1 [file peerj-11-15441-s001.zip › Raw data submitted/cell apoptosis/Fig. 8/AGSCDDP+si-MFAP2/Exp2_AGSCDDP+si-MFAP2-3_Plot1.bmp]

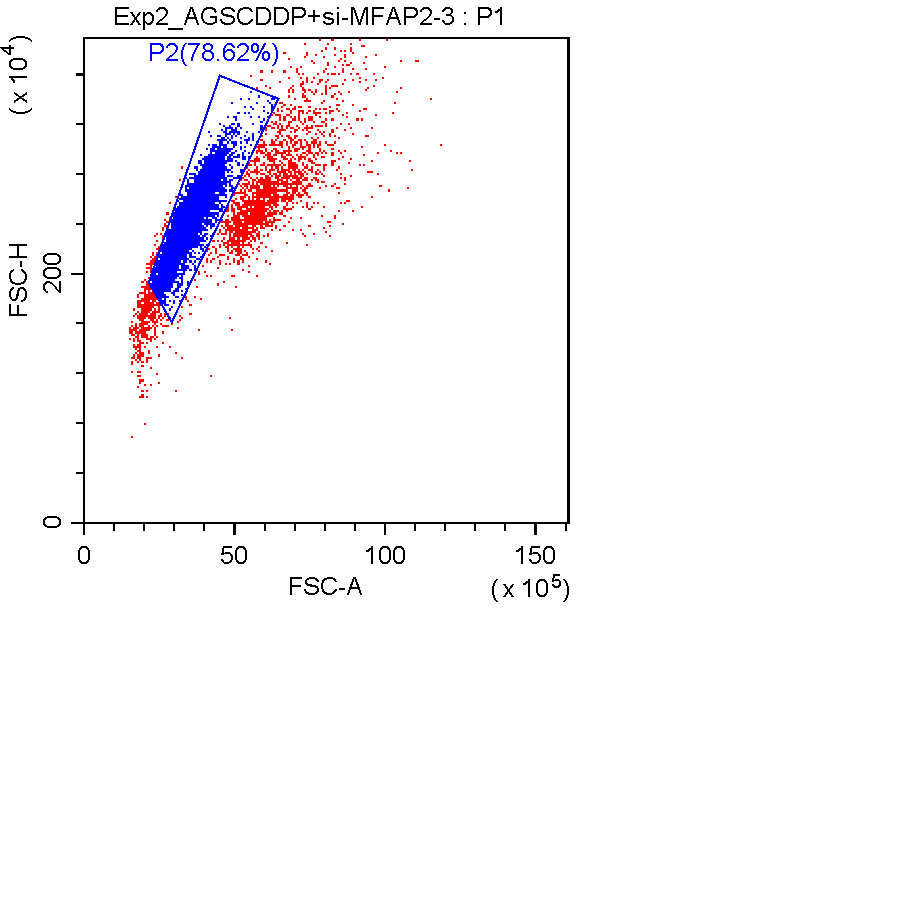

Supplement: Supplemental Information 1 [file peerj-11-15441-s001.zip › Raw data submitted/cell apoptosis/Fig. 8/AGSCDDP+si-MFAP2/Exp2_AGSCDDP+si-MFAP2-3_Plot2.bmp]

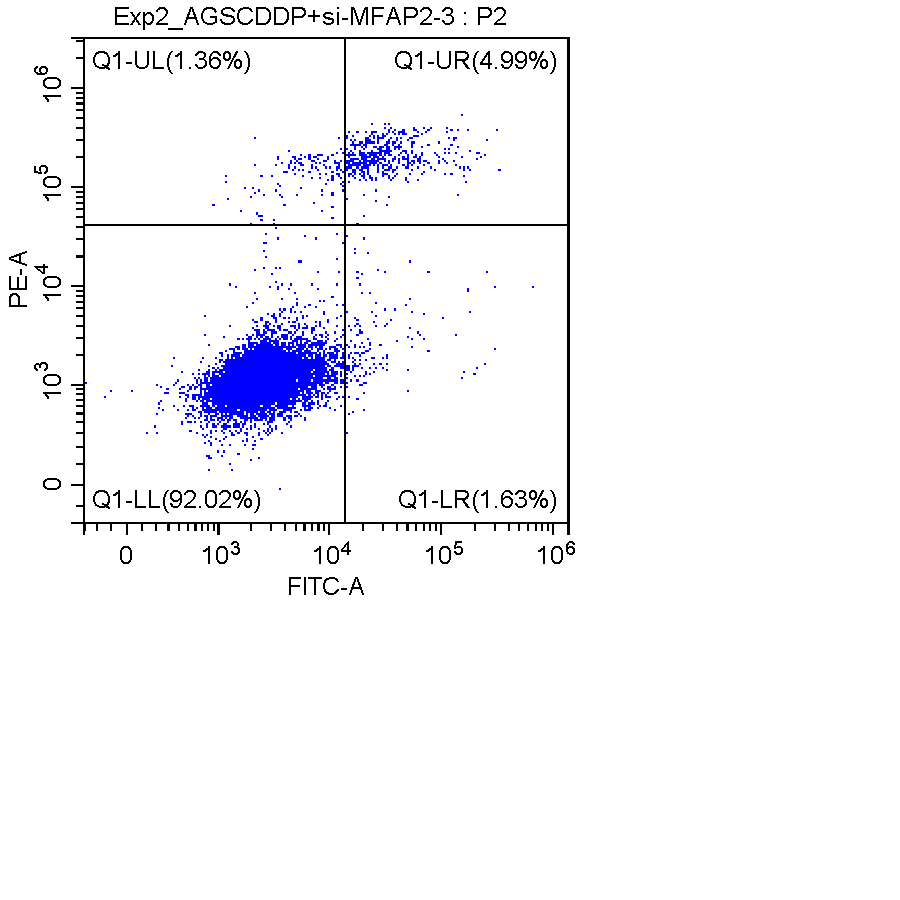

Supplement: Supplemental Information 1 [file peerj-11-15441-s001.zip › Raw data submitted/cell apoptosis/Fig. 8/AGSCDDP+si-MFAP2/Exp2_AGSCDDP+si-MFAP2-3_Plot3.bmp]

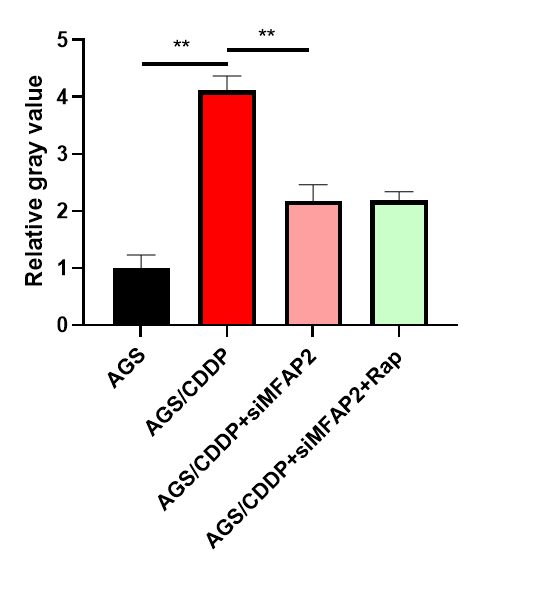

Supplement: Supplemental Information 1 [file peerj-11-15441-s001.zip › Raw data submitted/statistical analysis/Fig. 10A.png]

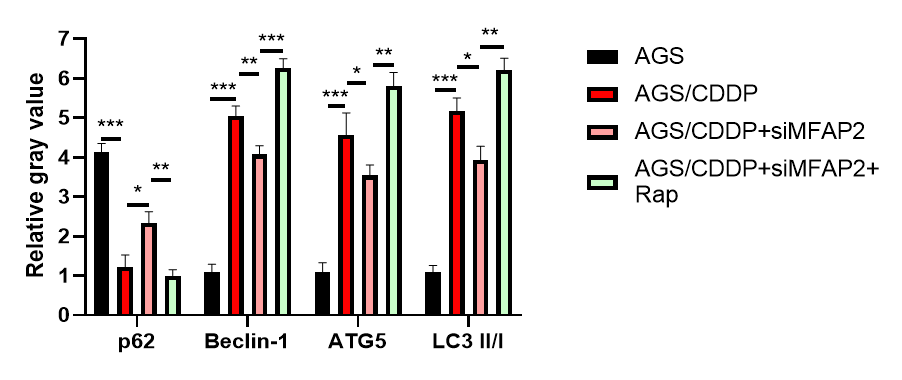

Supplement: Supplemental Information 1 [file peerj-11-15441-s001.zip › Raw data submitted/statistical analysis/Fig. 10B.png]

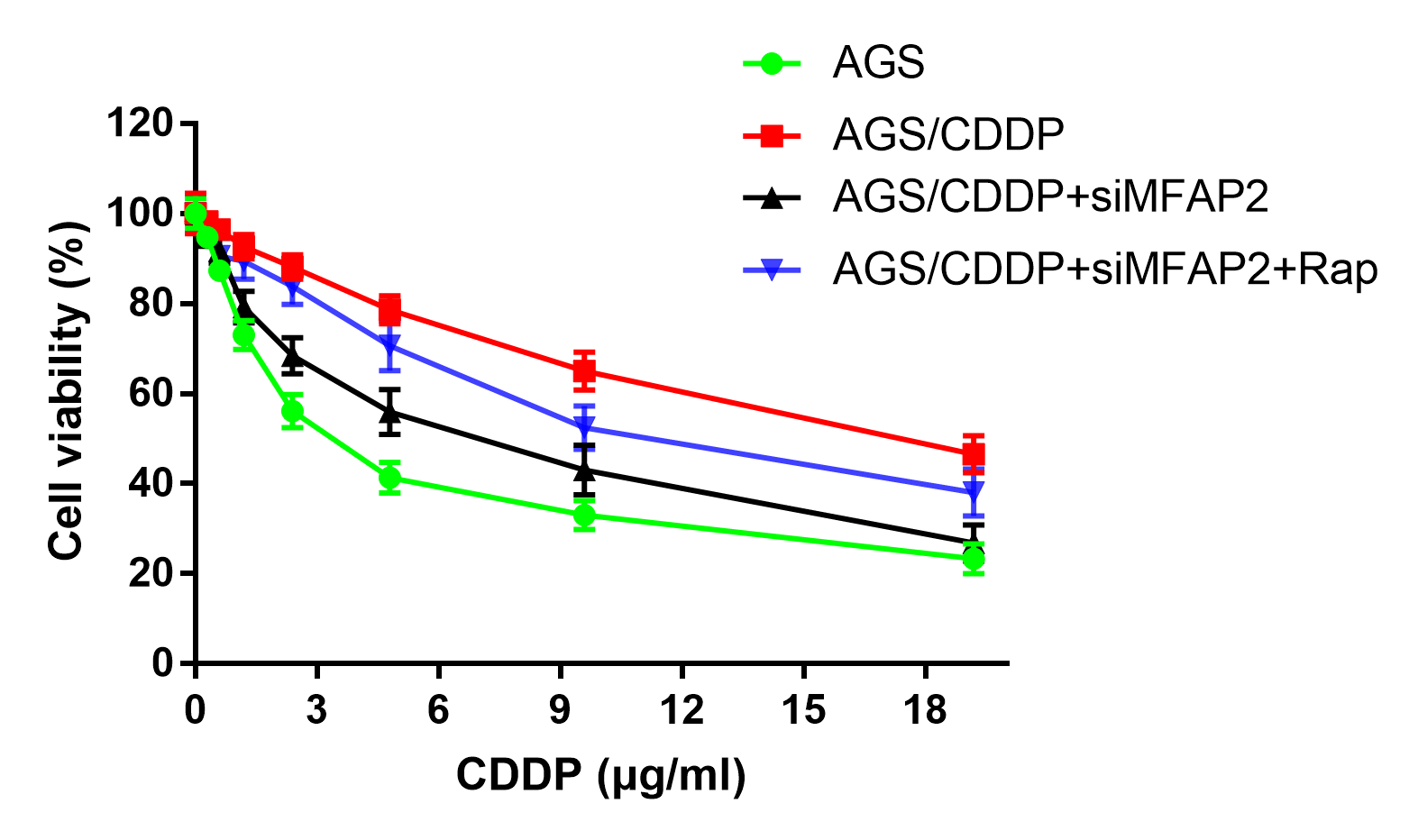

Supplement: Supplemental Information 1 [file peerj-11-15441-s001.zip › Raw data submitted/statistical analysis/Fig. 10C.png]

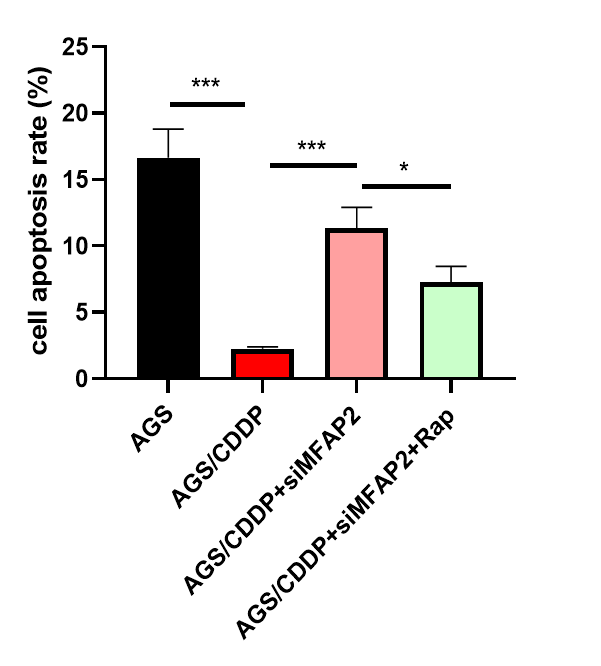

Supplement: Supplemental Information 1 [file peerj-11-15441-s001.zip › Raw data submitted/statistical analysis/Fig. 10D.png]

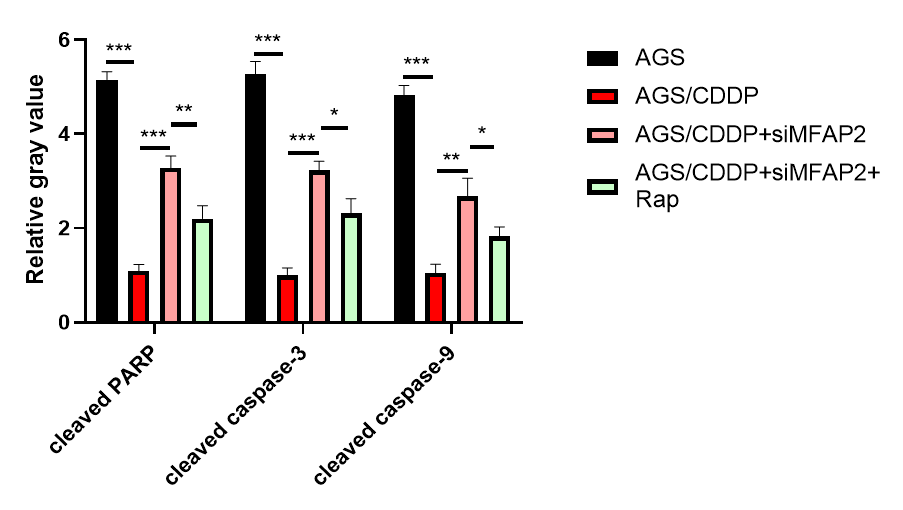

Supplement: Supplemental Information 1 [file peerj-11-15441-s001.zip › Raw data submitted/statistical analysis/Fig. 10E.png]

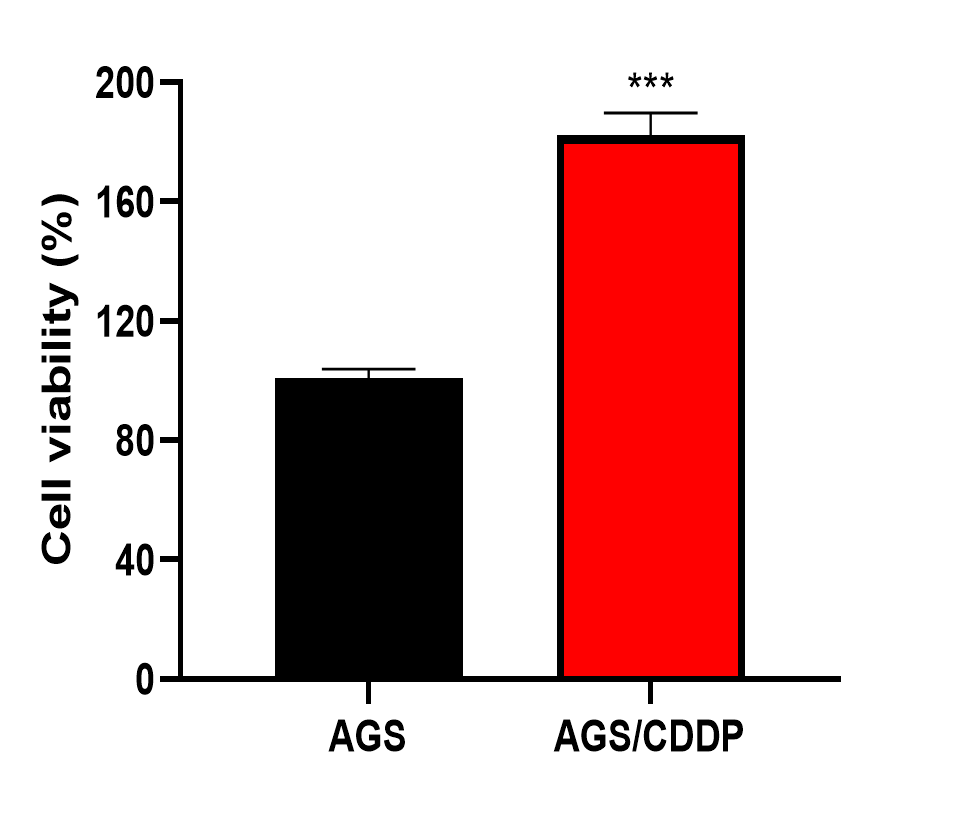

Supplement: Supplemental Information 1 [file peerj-11-15441-s001.zip › Raw data submitted/statistical analysis/Fig. 7A.png]

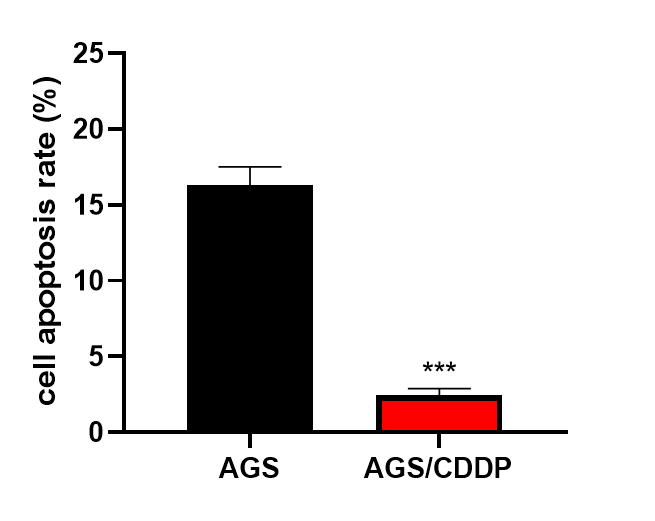

Supplement: Supplemental Information 1 [file peerj-11-15441-s001.zip › Raw data submitted/statistical analysis/Fig. 7B.png]

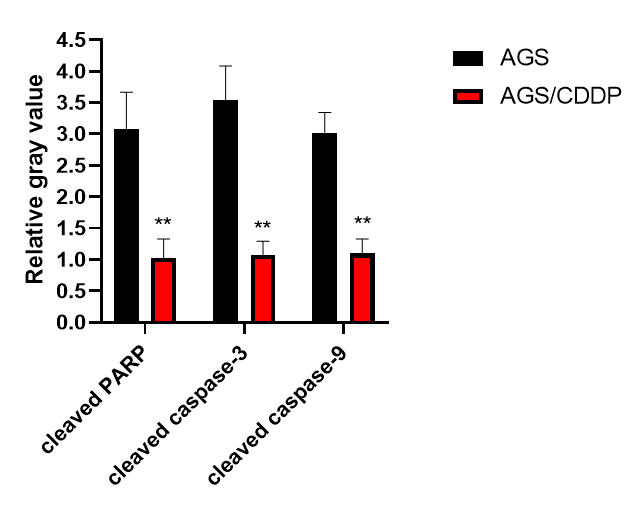

Supplement: Supplemental Information 1 [file peerj-11-15441-s001.zip › Raw data submitted/statistical analysis/Fig. 7C.png]

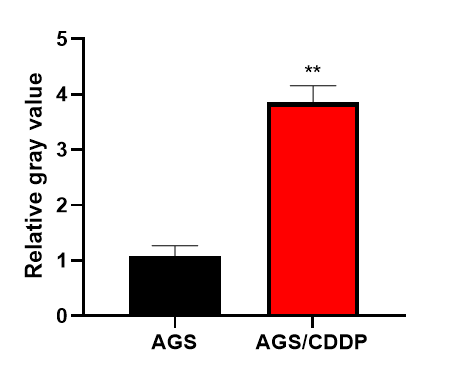

Supplement: Supplemental Information 1 [file peerj-11-15441-s001.zip › Raw data submitted/statistical analysis/Fig. 7D.png]

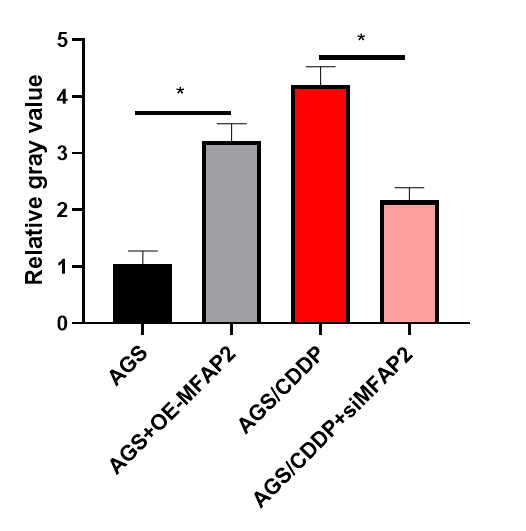

Supplement: Supplemental Information 1 [file peerj-11-15441-s001.zip › Raw data submitted/statistical analysis/Fig. 8A.png]
